# A Bibliometric Analysis of Cyclophosphamide, Methotrexate, and Fluorouracil Breast Cancer Treatments: Implication for the Role of Inflammation in Cognitive Dysfunction

**DOI:** 10.3389/fmolb.2021.683389

**Published:** 2021-08-20

**Authors:** Christa Corley, Antiño R. Allen

**Affiliations:** ^1^ Division of Radiation Health, University of Arkansas for Medical Sciences, Little Rock, AR, United States; ^2^ Department of Pharmaceutical Sciences, The University of Arkansas for Medical Sciences, Little Rock, AR, United States; ^3^ Neurobiology & Developmental Sciences, The University of Arkansas for Medical Sciences, Little Rock, AR, United States

**Keywords:** cyclophosphamide, methotrexate, fluorouracil, brain, inflammation

## Abstract

Inflammation is considered one of the possible mechanisms behind long-term cognitive dysfunction persistent after chemotherapy treatment. The chemotherapy combination of cyclophosphamide, methotrexate, and fluorouracil (CMF) was one of the older methods of treating breast cancer patients. Decades later, these patients still report experiencing cognitive side effects. In this present bibliometric review, we applied the VOSviewer tool to describe the existing landscape on literature concerning inflammation as it relates to CMF and cognitive dysfunctions. As time progressed, we saw an increase in interest in the topic. By the mid-2010s there were approximately 1,000 publications per year. Terms related to the brain and CNS did not appear until the later years, and terms related to inflammation and breast cancer were very prevalent throughout the three decades. Also, in more recent years, inflammatory markers and plant-derived compounds used to alleviate side effects of the inflammatory response appeared in the search results. The USA remained the most prolific producer of CMF-, inflammation-, and cognitive dysfunction-related papers throughout the three decades followed by Asia and Europe. As research of cognitive dysfunction caused by inflammation due to chemotherapy treatment progresses, more opportunities emerge for therapeutic methods to improve the quality of life for long-term survivors.

## Introduction

Like many medicines, chemotherapy began as derivatives of numerous natural sources and was created for various applications, but coincidently became means for treating diseases such as cancer. One of the earliest known findings of these drugs showing chemotherapeutic effect, which could be used to treat a type of cancer, was a serendipitous discovery during World War I when soldiers were exposed to nitrogen mustard. The idea of combining two or more chemotherapeutic agents was inspired using antibiotic combination therapy to reduce the risk of resistance (Morrison, 2010). Combination therapy was then adopted and became a very effective treatment of cancers. The combination of these drugs also provided several benefits, such as decreasing drug resistance, increasing the number of targets, addressing tumor heterogeneity, having the ability to lower the dosage of one or more of the drugs in the cocktail, and having synergistic effects (Mokhtari et al., 2017).

After seeing a reduction in leukocytes in soldiers exposed to mustard gases in World War II, nitrogen mustard derivatives were investigated as a chemotherapeutic agent. Clinical trials using nitrogen mustard showed that after a series of x-ray therapy sessions, large tumor masses dissolved with continual injections in various terminal stage carcinomas (Gilman and Philips, 1946). These nitrogen mustard derivatives developed into cyclophosphamide. Studies on fluorinated pyrimidines, such as 5-fluorouracil showed tumor inhibitory actions (Heidelberger et al., 1957a; Heidelberger et al., 1957b). The first use of the combination chemotherapy treatment of cyclophosphamide, methotrexate, and fluorouracil (CMF) for breast cancer in clinical trials was performed in the late 70 s (Bonadonna et al., 1976). These clinical trials resulted in a decrease in recurrence of breast cancer within the first 2–3 years following a mastectomy (Bonadonna et al., 1976). Although CMF was used for 30 years to treat early-stage breast cancer, clinical trial studies were limited in participation numbers, often the population was less than 200 patients and did not represent individuals with various cancer characteristics such as triple-negative breast cancer. Long-term effects were also not considered when treating these patients.

Advances in diagnostics and treatment of breast cancer have greatly improved survival. With an increased number of survivors, there has been an increased number of reports of cognitive decline. Chemotherapy-induced cognitive impairment (CICI), or also known as chemobrain, is a poorly understood occurrence. The National Health and Nutrition Examination Survey reported a 40% increase in cancer survivors reporting issues of cognitive decline in 2014 (Moore, 2014). In a study with patients that completed treatments with the CMF therapy were found to have cognitive defects assessed beside a control group. Common symptoms of CICI include acute and delayed deficits in learning and memory, concentration, executive function, and processing speed. Chemobrain symptoms can persist for years post-chemotherapy, and some patients never regain their previous quality of life (Correa and Ahles, 2008; Christie et al., 2012; Wefel et al., 2015; Deprez et al., 2018).

Within the past decade, interest in how neuroinflammation caused by chemotherapy affects brain function has increased especially in the newer forms of chemotherapy regimens. Since CMF was only used for a short period before the 21st century, research on its cognitive effects is limited. Although CMF is not currently used patients who received the treatment are still alive thus understanding the implications of CMF-induced inflammation can have on their quality of life is extremely relevant as well as essential to our understanding of how other breast cancer drug treatments may impact patient care long term. A review published by McLeary et al. (2019) does a good job of summarizing the current literature published on the neuroinflammatory effects of chemotherapy (McLeary et al., 2019). From this article, we know that innate immune cells such as microglia and astrocytes are majorly affected by the individual chemotherapy drugs (McLeary et al., 2019). The immune response negatively impacts the functionality of these cells leading to a cascade of alterations in central nervous system function and neurodegeneration. We intend to examine how the topics of CMF and inflammation have increased in interest and how research involving the brain comes into play by using a bibliometric approach to analyze published literature.

**FIGURE 1:**
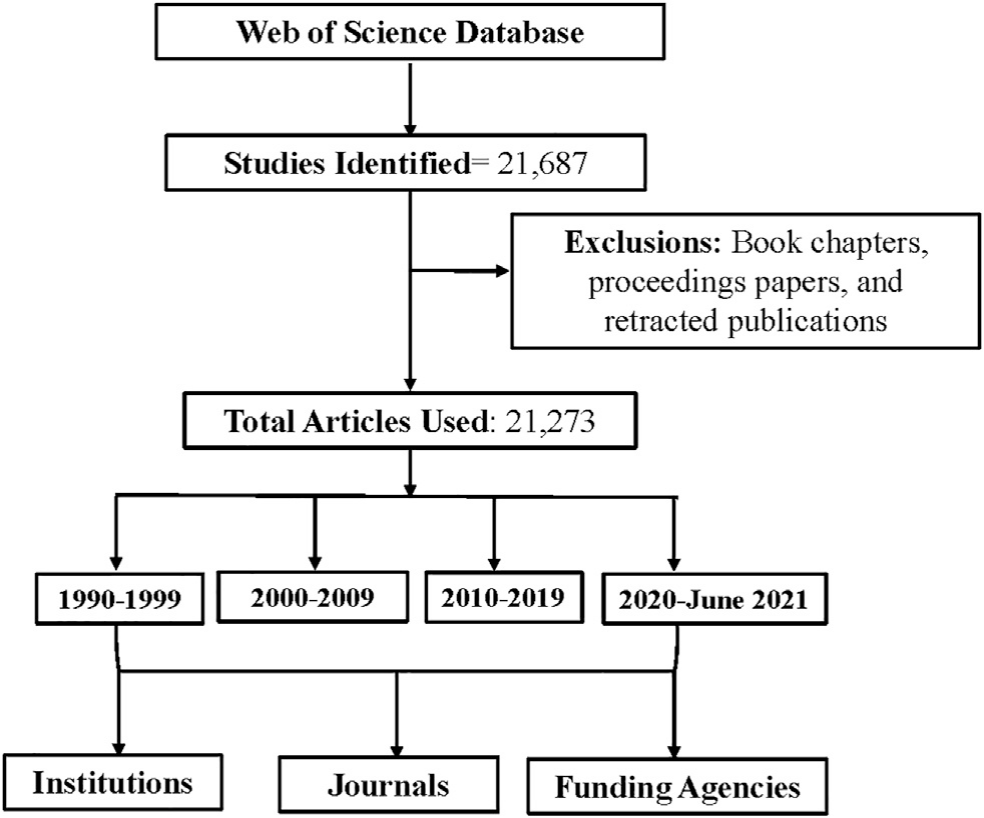
This flowchart show methods and parameters used to design this study.

The term bibliometric was coined in 1969 by Alan Pritchard. The analysis provides several different benefits for researchers such as, the impact of research outputs and identifying developments of a topic taken from a large database. A bibliometric analysis is a quantitative method used to analyze publications based on variables such as authors, countries, institutions, etc. (Ellegaard and Wallin, 2015). Within the last decade, it has been established that chemotherapy is involved in brain dysfunction; we aim to use the bibliometric analysis approach to evaluate how research on these terms has shifted from basic science to more clinical related. We will be evaluating the key terms generated from VOSveiwer and journals and institutions retrieved from Web of Science. Although CMF is no longer used as a regimen to treat breast cancer, our purpose is to depict the relevance of CMF and inflammation and discuss how cognition comes into play with these terms. Methods for this review can be found in supplementary materials. Figure 1 gives a simple schematic for the flow of the methods.

## Results

### Output of Research on Cyclophosphamide, Methotrexate, and Fluorouracil and Inflammation in Publication Form has Increased From 1990 to Mid-2021

**FIGURE 2:**
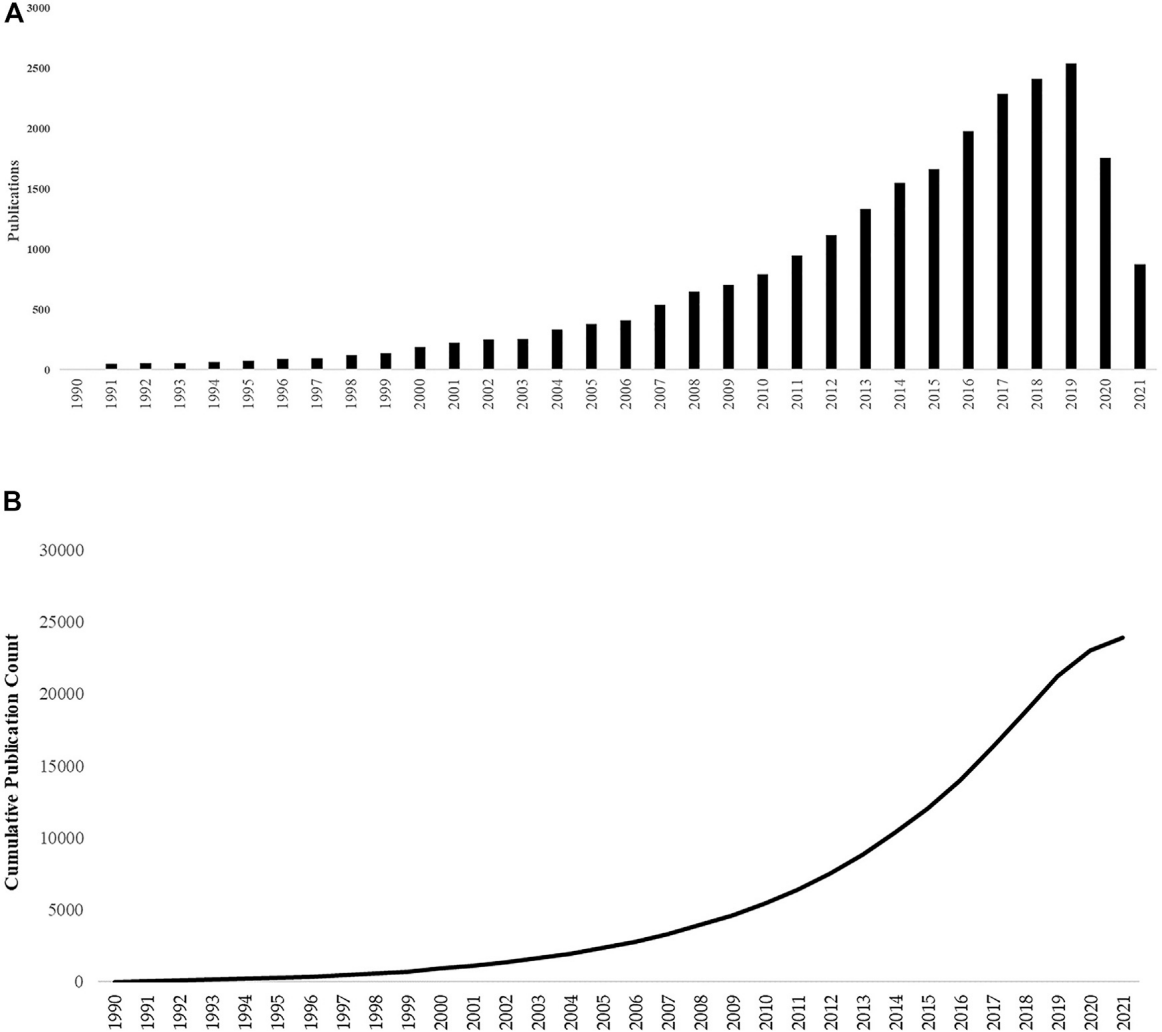
**(A)** The graph depicts the total number of publications over the years. **(B)**: The graph depicts the accumulation of publications throughout the 3 decades.

The Web of Science search generated 21,273 publications. Separated into time points, the 1990s had 727 publications, the 2000s had 3,915 publications, and the 2010s had 16,617 publications. The 1990s began with only three publications. There was a steady increase in the release of publications as time progressed. Publication count did not exceed over 1,000 until the 2010s. This trend is continuing into 2020. We can assume that as the year 2021 continues, more publications will be released on these topics. An exponential increase was not seen until the latter decade. Both graphs show an incline in publication counts. **(** Figures 2A,B **).** Figure 2A gives the total number of publications for each and shows each year there is an increase. Figure 2B gives an accumulation of the publication and how these articles have incorporated themselves each year. The total number of publications and accumulation of publications both show a steady upward trend.

### Terminology Related to Cyclophosphamide, Methotrexate, and Fluorouracil and Inflammation Expand and Evolve

**FIGURE 3:**
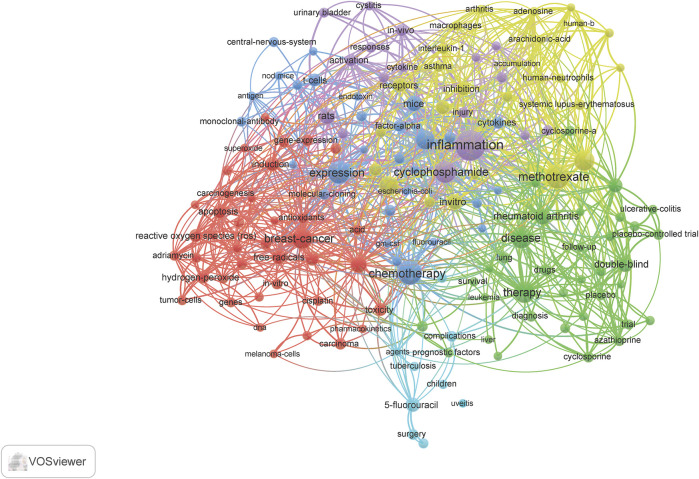
Term map showing the visualization of 154 terms that occurred at least 5 times in documents between 1990 and 1999. Refer to Supplementary Table S2 for a complete list of terms.

**FIGURE 4:**
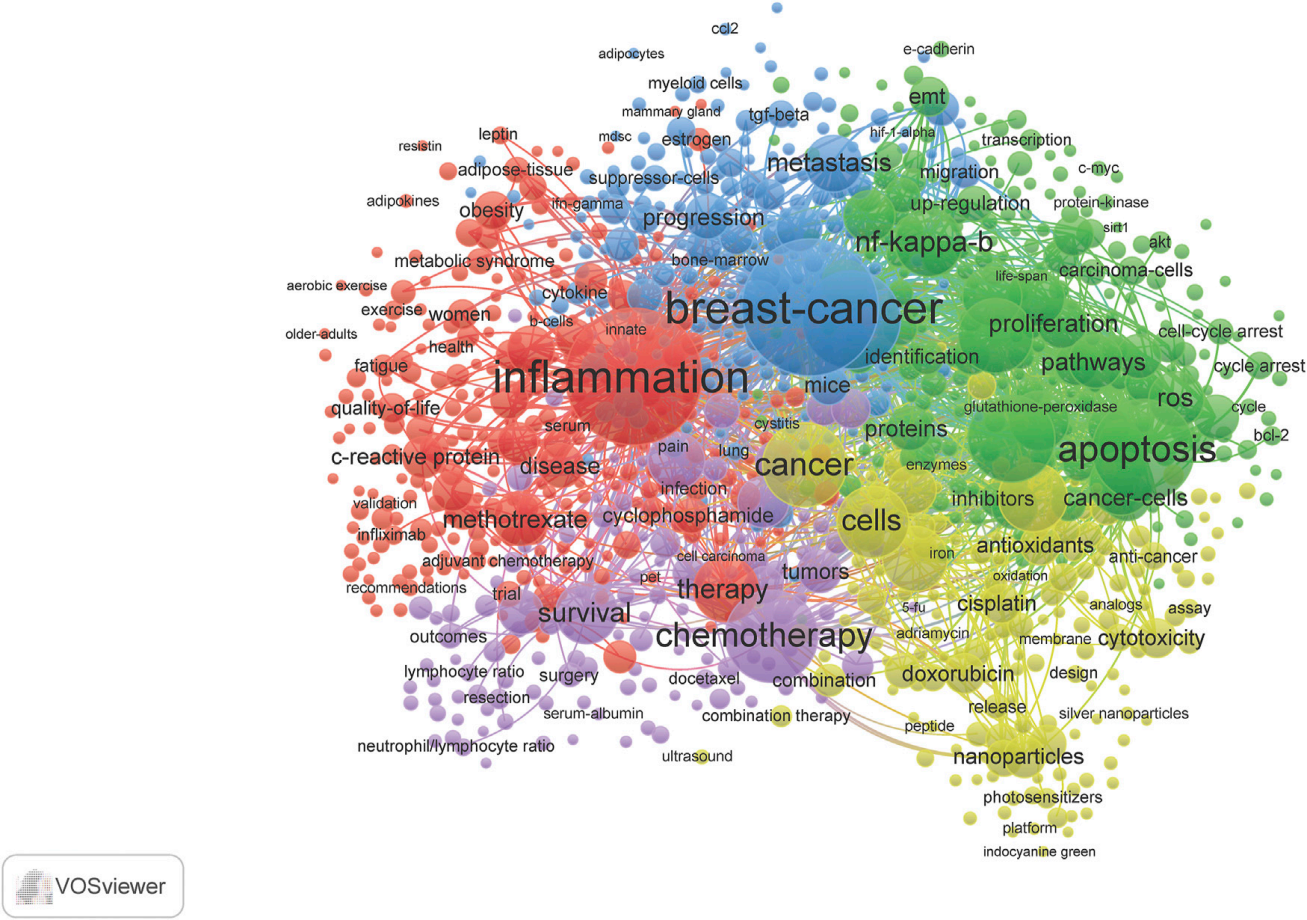
Term map showing the visualization of 669 terms that occurred at least 10 times in documents between 2000 and 2009. Refer to Supplementary Table S3 for complete list of terms.

**FIGURE 5:**
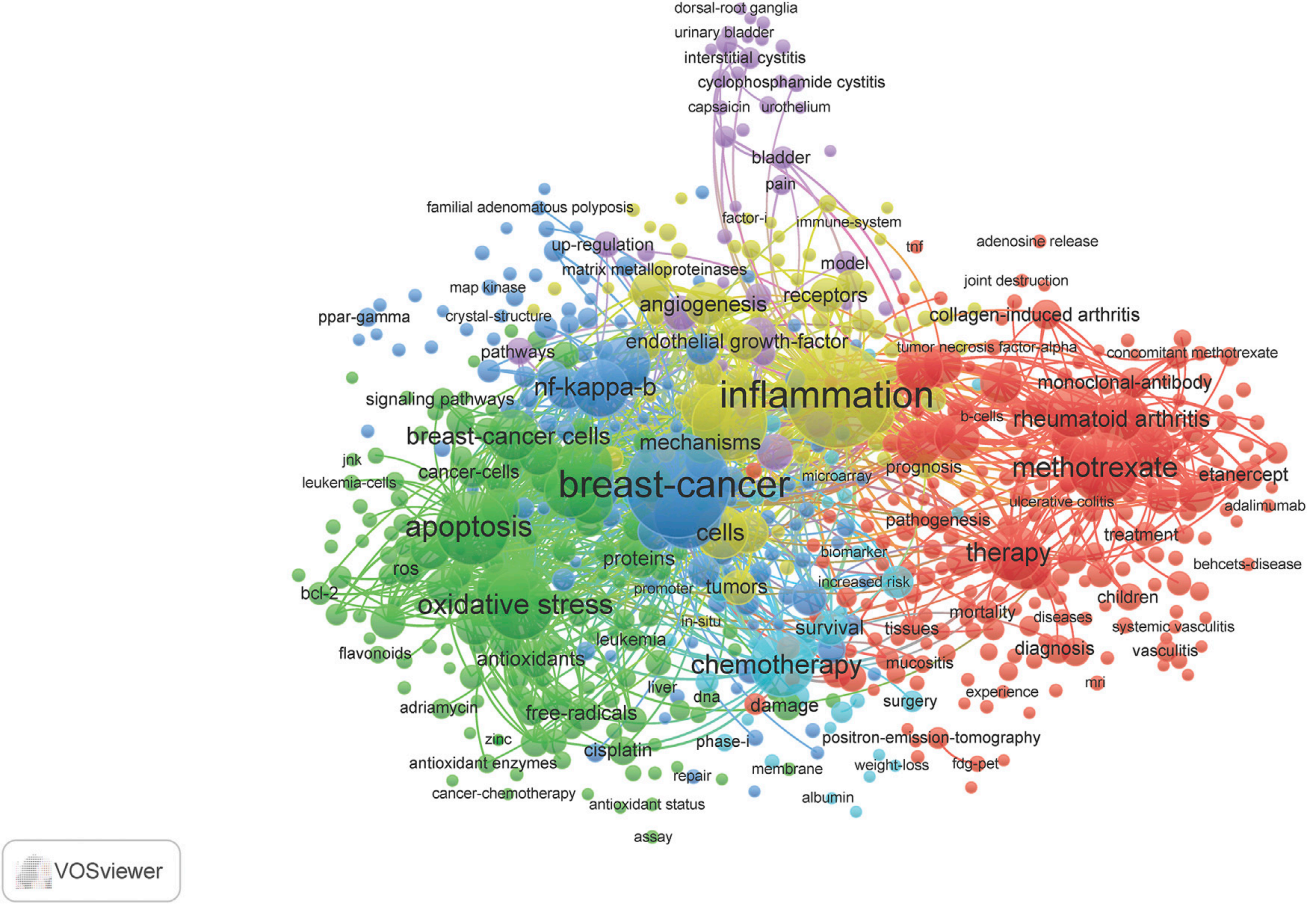
Term map showing the visualization of 983 terms that occurred at least 25 times in documents between 2010 and 2019. Refer to Supplementary Table S4 for a complete list of terms.

**FIGURE 6:**
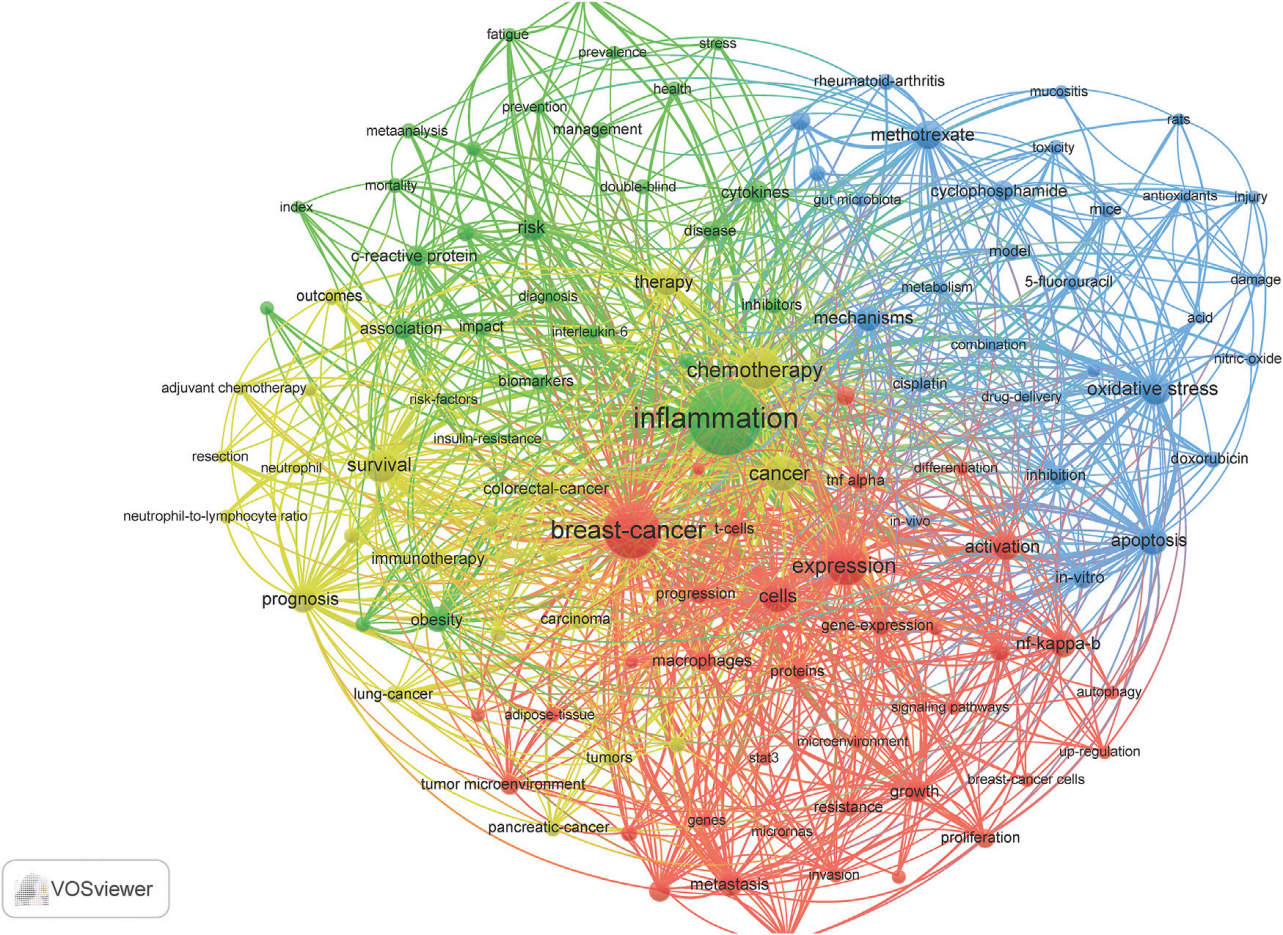
Term map shows a visualization of 118 terms that occurred at least 20 times in the most current document from 2020 to mid-2021. Refer to Supplementary Table S5 for complete list of terms.

Term maps were generated for each time **(** Figures 3–6 **).** Term maps were developed to depict relationships and occurrences of terms related to the search topics. The size of circles increases with an increase in occurrences. Colors group each term into clusters with other terms that are closely linked to each other. Figure 3 and Supplementary Table S3 depict terms from 1900–1999 for a total of 154 terms, 6 clusters, 1,931 links, and a total link strength of 2,846. Within these terms, cyclophosphamide, methotrexate, and fluorouracil are found in low occurrences (ranging from 15–50) with fewer linkages (ranging from 10–80). Terms related to inflammation were also limited to terms such as inflammation, itself, cytokines, IL-6, IL-1, macrophages, neutrophils, and t-cells. Based on these key terms and their occurrences, articles involving clinical/human trials occurred 38 times. This can be suggested by terms such as children, placebo-controlled trials, and controlled clinical trials. Rat and mouse occurred 43 times and *in vivo* occurred 21 times. When comparing this to terms such as cells, which occurred 28 times, and *in vitro*, which occurred only 21 times, we can postulate that there is approximately a balanced amount of *in vitro* to *in vivo* work done during this time.

Figure 4 and Supplementary Table S4 depict terms from 2000–2009 for a total of 669 terms, 6 clusters, 33,510 links, and total links strength of 78, 305. This decade showed a significant increase in occurrences of cyclophosphamide, methotrexate, and inflammation. It is in this decade combination therapy and adjuvant therapy appear. Terms that are related to inflammation included chronic inflammation, T cells, macrophages, immune response, infection, neutrophils, NF-kappa B, IL-6, B-cells, Cox-2, IFN-gamma, immunomodulation, IL-10, IL-8, lymphocytes, NF-kappa-B, neutrophil, cytokines, and chemokines. Terms related to the brain include dorsal root ganglia and the blood-brain barrier. Within the top ten most occurring terms in this decade, inflammation ranked number two. Also, methotrexate was found in the top ten. Various diseases related to inflammation appeared in this search such as arthritis and inflammatory bowel disease. When investigating the types of studies at this time point, clinical trials (40), controlled trials (82), phase I (33), phase II (37), placebo-controlled trial (63) are all related to human studies. Occurrences are found in parentheses. Mice and rats occurred 231 times and cells 206 times. *In-vitro* occurred 52 times more than *in vivo*.

Figure 5 and Supplementary Table S5 visualizes the terminology analysis within the years 2010–2019. The 2010s had a total of 983 terms, 5 clusters, 112,405 links, and a total link strength of 449,497. The terms blood-brain barrier and cognitive impairments appear in this collection of related terms. Inflammation appears as the second most occurring term in this search. Some additional terms that appeared in this decade compared to others include anti-inflammation, anti-inflammatory, cancer-related inflammation, IL-17, and proinflammatory cytokines. Through the decades, there is approximately a 2-fold increase in the occurrences and linkages of these terms. There is an increase in occurrences of clinical trial-related research. Phase I (63), phase II (172), and phase III (108) clinical trials all appear in this decade’s term map. This correlates with an increase in types of inflammatory drugs and plant-derived compounds with anti-inflammatory properties which have appeared. The term clinical practice guidelines suggested more education material became available. Surprisingly, *in vitro* occurs twice as much as *in vivo*, and t cell appears 395 times more than rat and mouse.

Figure 6 and Supplementary Table S6 conclude the terminology analysis between 2020-June 2021 to evaluate the most current publications available for the topics in discussion. This year and a half had a total of 118 terms, 4 clusters, 3,732 links, and a total length strength of 14,572. The terms in the 4 clusters carry somewhat of a theme. Cluster 1 has terms relevant to molecular studies with terms linking more to breast cancer and expression. Terms in cluster two suggest a term involving risk factors with terms linking to inflammation, women, and association the most. Cluster 3 may suggest *in vivo*/vitro studies on chemotherapy combinations. Lastly, terms such as survival, resection, chemotherapy being the highest linked terms, it could be suggested that cluster four involves survival research for cancer.

There were a few terms found common in the 3 decades and the most current years, which include inflammation, breast cancer, expression, and chemotherapy. Terms relating to inflammation were particularly prevalent in this search. This is confirmed by the contingency of the key terms such as inflammation, inflammatory response, immune response, chemokines, infection, and cytokine. Terms such as cells, mouse and rat, clinical trial, and phase I and phase II imply the importance of *in vitro*, *in vivo*, and clinical trial studies in this search, also term survival suggests studies on cancer survivors.

### Productive of Journals During Each Time Frame

From 1990–1999, the journal with the most publications on said topics was the Journal of Rheumatology containing 2.5% of the articles related and the most cited articles. (Table 1). The trend in the interest of publications in this decade was heavily rheumatology related. These journals covered a range of different article types such as clinical research, meta-analysis, editorials, and other educational material. Clinical interest in inflammation-related studies can be found in such journals as Free Radical Biology and Medicine and the Journal of Immunology (Table 1). Some cancer-related research can be identified in publications in Cancer Research (Table 1).

**TABLE 1:** Most productive journals from 1990–1999.

Journal	Number of articles, %	Citing articles	Citation per publication^a^	Impact factor^b^
Journal of Rheumatology	18 (2.48)	262	14.56	2.88
Ophthalmology	12 (1.65)	90	7.50	2.73
Arthritis and Rheumatism	11 (1.51)	225	20.45	7.05
Cancer Research	8 (1.10)	225	28.13	8.16
British Journal of Rheumatology	7 (0.96)	33	4.71	2.85
Free Radical Biology and Medicine	7 (0.96)	147	21	4.08
Journal of The American Academy of Dermatology	7 (0.96)	97	13.86	1.01
Biochemical Pharmacology	6 (0.83)	99	16.50	2.76
Journal of Immunology	6 (0.83)	47	7.83	7.15

a Equals citing articles divided by the number of publications by the same journal.

b Retrieved from: Selected Scientific Journal- http://www.genebee.msu.su/journals/if99b.html

In the following decade, the 2000s, there was a significant increase in publication output from the top journal with articles related to the topic at hand. Here begins the shift in interest from rheumatology to more cancer-related topics. As shown, Cancer Research outputs the most articles approximately 2% of the articles for the decade (Table 2), also the second highest number of cited articles behind Journal of Biological Chemistry. These articles remained highly clinical related and educational material as suggested by publications in Arthritis and Rheumatism, Clinical Cancer Research, and Free Radical Biology and Medicine.

**TABLE 2:** Most productive journals from 2000–2009.

Journal	Number of articles	Citing articles	Citation per publication*	Impact factor**
Cancer Research	83 (2.12)	1,422	17.13	7.543
Arthritis and Rheumatism	59 (1.50)	778	13.19	7.332
International Journal of Cancer	52 (1.33)	1,042	20.04	4.722
Journal of Biological Chemistry	46 (1.17)	1,579	34.33	5.328
Clinical Cancer Research	40 (1.02)	860	21.50	6.747
Journal of Rheumatology	39 (0.99)	878	22.51	3.854
Free Radical Biology and Medicine	38 (0.97)	844	22.21	6.081
Annals of the Rheumatic Diseases	37 (0.94)	1,116	30.16	8.111
International Journal of Antimicrobial Agents	36 (0.92)	34	0.94	3.032
Cancer Letters	34 (0.87)	874	25.71	3.741

As we exam journals with the most output in the 2010s, it is apparent that there had been a complete shift from rheumatology as an area of interest. The top contributor of articles and citations for this decade is PloS One, approximately 3% of publications and over 5,000 citations (Table 3). As stated from their site, this journal publishes multidisciplinary natural and clinical studies. Oncotarget, which is second in ranking for articles and citation, carries a focus on multidisciplinary research on molecules, pathways, cellular functions, cell types, and tissues used as means to target cancer whether for treatment or diagnosis. Scientific Reports like PloS One is also multidisciplinary in natural and clinical studies. This journal has been deemed to facilitate innovation and progression in various fields of interest. This decade provides us areas of interest related to clinical studies, basic science, innovation, and education.

**TABLE 3:** Most productive journals from 2010–2019.

Journal	Number of articles	Citing articles	Citation per publication	Impact factor^b^
PloS One	488 (2.94)	5,021	10.29	2.776
Oncotarget	371 (2.23)	4,526	12.20	3.710
Scientific Reports	233 (1.40)	3,952	16.96	3.998
International Journal of Molecular Sciences	186 (1.12)	3,527	18.96	4.210
Cancer Research	155 (0.93)	1,335	8.61	9.130
Biomedicine Pharmacotherapy	146 (0.88)	1,685	11.54	4.545
BMC Cancer	143 (0.86)	1,209	8.45	3.288
Oncology Reports	138 (0.83)	1,231	8.92	3.417
Oncology Letters	123 (0.74)	1,453	11.81	1.554
Cancer Letters	111 (0.67)	1,231	11.09	7.360

a Equals citing articles divided by the number of publications by the same journal.

b Retrieved from webpages of the journals.

In the most recent year, between 2020 and mid-2021, we see the journals Cancer, International Journal of Molecular Sciences, Frontiers in Oncology and Pharmacology, and Scientific Reports housing publications related to these topics. Cancer having the most published article (Table 5) focuses on clinical research findings involved in cancer research, risk reduction, treatment, and patient care. With the second most article, the International Journal of Molecular Sciences focuses on biochemistry, molecular and cell biology, molecular biophysics, molecular medicine in chemistry. These years seem to focus on clinical studies and molecular research.

## Discussion

The present study gives a descriptive analysis of the relevance of CMF and inflammation over 3 decades. This analysis demonstrated how over time a particular research area can continue to expand as the present need for further investigation grows. For example, the field of chemotherapy and inflammation research is continuing to expand, as reflected in Figure 2. Both the total number of publications and the accumulation of these articles have increased. Publications reached over 1,000 in the 2010s and increased thereafter, and there is an increasing variety of research areas involved. These publications were not limited to basic research, such as cell biology, pharmacology, toxicology, biochemistry, and/or molecular biology. Most articles represented clinical topics such as immunology, rheumatology, and oncology. Based on previous research, CMF was not used as a combined treatment until the 70 s. However, interest in the subject became more popular in the 2000s. From Figures 3–6 and their supplementary tables, there is a large increase in the occurrence of terms related to inflammation. In the 2000s and 2010s, we see the inclusion of brain related terms with cognitive impairments appearing in the latter decade. This indicates an increased interest in chemotherapy induced cognitive impairment. These terms also indicate the relevance of *in vitro*, *in vivo*, and clinical trials studies.

### Institutional Trends in Cyclophosphamide, Methotrexate, and Fluorouracil and Inflammation Research

The US was a leading producer of publications and led in citing articles employing institutional contributions (Tables 4). Over the 3 decades, the University of California System, University of Texas System, and Harvard University remained leaders in published and citing work. The second most productive institutions were in Europe; INSERM and Assistance Publique Hopitaux in Paris, France, and the University of London in London, England. Lastly, the Chinese Academy of Science only made a large contribution in representing Asian countries. Even in the most recent years, the US has led in the contributions of publications. Havard University, the University of California System, and the University of Texas Systems have been the highest producers from 2020 to mid-2021 (Table 5).

**TABLE 4:** Most productive institutions from 1990–2019.

Institutions	Number of articles	Number of citing articles	Citation per publication
University of California System	586	11,958	20.41
University of Texas System	574	10,619	18.50
Harvard University	520	7,728	14.86
National Institutes of Health NIH USA	412	7,037	17.08
MD Anderson Cancer Center	301	5,883	19.54
Institut National de la Sante Et De La Recherche Medicale Inserm	379	6,890	18.18
Chinese Academy of Sciences	256	7,071	27.62
University of London	73	6,604	90.47
Assistance Publique Hopitaux Paris (APHP)	77	2,689	34.92
Johns Hopkins University	70	4,865	69.50

a Equals citing articles divided by the number of publications by the same institution.

**TABLE 5:** Most current data on journal and institutional productivity from 2020 to mid-2021.

Journal	Number of articles	Citing articles	Citation per publication^a^	Impact factor^b^
Cancers	114 (4.33)	270	2.37	6.639
International Journal Of Molecular Sciences	66 (2.5)	321	4.86	4.556
Frontiers In Oncology	54 (2.05)	151	2.80	4.848
Frontiers In Pharmacology	33 (1.25)	83	2.52	5.33
Scientific Reports	33 (1.25	53	1.60	4.379
**Institutions**	**Number of articles**	**Number of citing articles**	**Citation per publication** ^c^	**—**
Harvard University	71	115	1.62	**—**
University Of California System	59	91	1.54	**—**
University Of Texas System	59	104	1.76	**—**
Harvard Medical School	48	115	2.40	**—**
Institut National De La Sante Et De La Recherche Medicale Inserm	45	132	2.93	**—**

a Equals citing articles divided by the number of publications by the same journal.

b Retrieved from: Journal website, most current impact factor for 2020.

c Equals citing articles divided by the number of publications by the same institution.

### Top Agencies Involved in Funding Research on Cyclophosphamide, Methotrexate, and Fluorouracil and Inflammation From 1990 to Mid-2021

Table 6 gives information on the funding agencies that funded the research on the topics being discussed**.** The US Department of Health and Human Services ranks first for funding close to 3,000 related articles. The National Institute of Health comes in second with 2,988 articles. The National Institutes of Health have been very active in funding research related to CMF and inflammation.

**TABLE 6:** Top 10 active funding agencies for published research related to CMF and inflammation for 1990–2021.

Funding agencies	Articles per funding agency (%)
United States Department Of Health Human Services	2,997 (17.63)
National Institutes Of Health NIH USA	2,988 (17.57)
NIH National Cancer Institute (NCI)	1708 (10.05)
National Natural Science Foundation Of China (NSFC)	1,343 (7.90)
European Commission	670 (3.94)
NIH National Heart Lung Blood Institute (HLBI)	371 (2.18)
Ministry Of Education Culture Sports Science And Technology Japan (MEXT)	364 (2.14)
NIH National Institute Of Diabetes Digestive Kidney Diseases (NIDDK)	352 (2.07)
Japan Society For The Promotion Of Science	273 (1.61)
NIH National Institute Of General Medical Sciences (NIGMS)	254 (1.49)

### Cyclophosphamide, Methotrexate, and Fluorouracil and Inflammation Research Publishes With Multidisciplinary Interests in Both Basic Sciences and Clinical Studies

The list of journals that have published at least 20 papers on CMF and inflammation from 1990 to 2019 began mainly focused on arthritis and transitioned to more cancer focused articles over the decades (Tables 1-3). Articles with interest in chemotherapy and inflammation appearing in the Journal of Rheumatology, Arthritis and Rheumatism, and Journal of Immunology during the 90 s may have been more focused on the use of methotrexate as a treatment for rheumatoid arthritis and viruses (including “old world alphaviruses”) (Bedoui et al., 2019). Although methotrexate was originally developed as an anticancer agent, it proved to be effective in the treatment of other diseases (Bannwarth et al., 1994; Aletaha et al., 2010; Marks and Marks, 2016). Cyclophosphamide was also investigated as means to treat arthritis. The 2000s represent heavy cancer related area of interest for the topics of focus in this review. Cancer focused journals such as Cancer Research and International Journal of Cancer provided most publications over rheumatology focused journals as compared to the previous decade. From the 2000s to the 2010s, there was a shift from cancer related research to more innovative, preclinical, and basic research. PloS One and Scientific Reports indicate during this period there was a multidisciplinary approach to innovation in science. For example, the Journal of Biological Chemistry mainly published basic studies or preclinical studies with mouse or rat models. When reevaluating the terms in this search (Figure 4), we see innovation in an increase in the occurrence of the three phases of clinical trials and an increase of natural compounds and xenobiotics with inflammatory properties.

### The Connection Between Cyclophosphamide, Methotrexate, and Fluorouracil, Inflammation, and Cognitive Dysfunction

**FIGURE 7:**
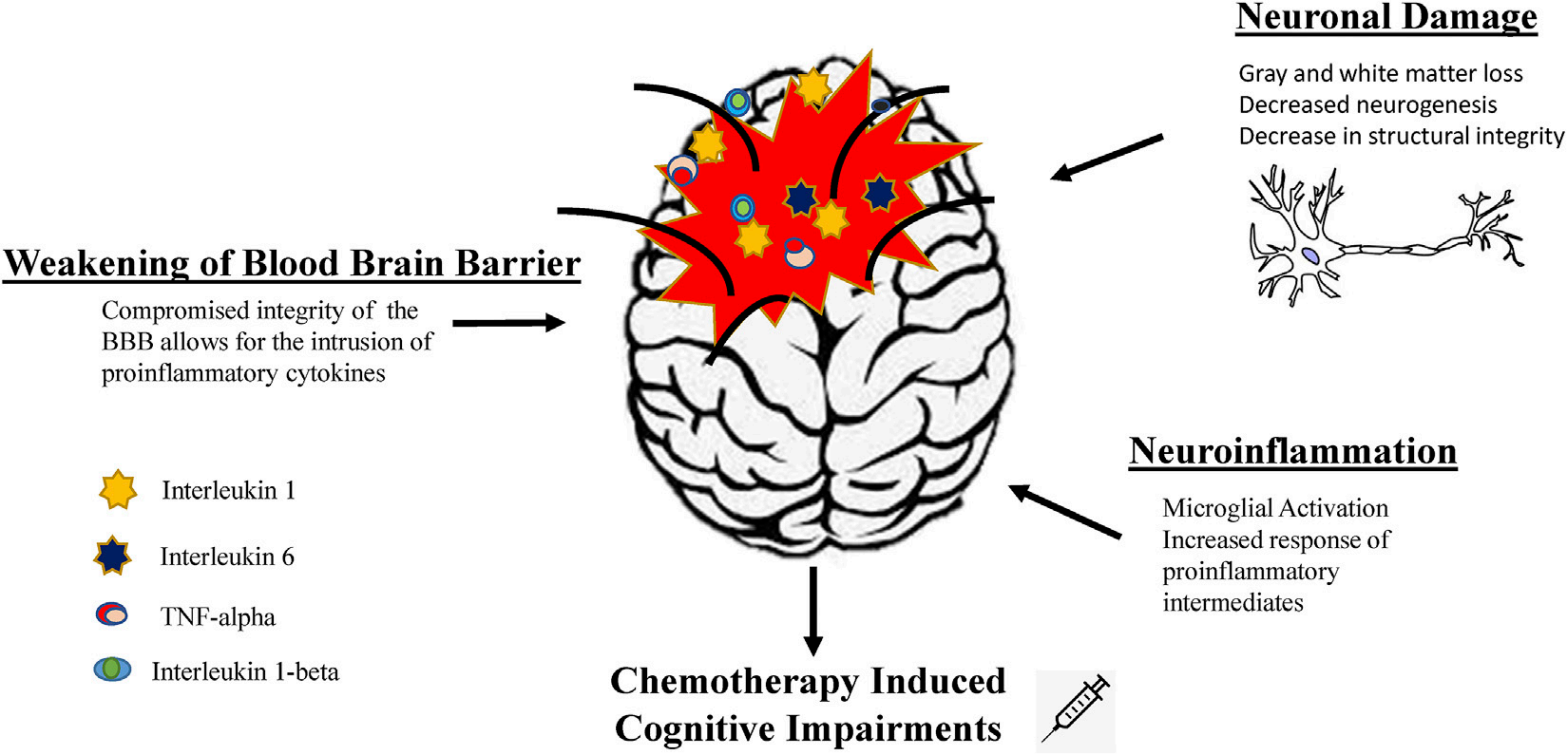
Simplified schematic for mechanisms of chemotherapy induced cognitive impairments. This schematic serves as an overview of factor associated with long-term cognitive dysfunctions via chemotherapy usage. These factors include neuroinflammation, neuronal dysfunction, and compromise in blood brain barrier integrity, which can directly and indirectly induce cognitive impairments.

The connection between inflammation and cancer is a well-established concept, and now it is one of the hallmarks of cancer. Extensive evidence has linked cancer treatment, such as chemotherapy, to cognitive dysfunction as a result of a significant increase in the inflammatory response. Immune cells in the inflammatory response can be beneficial to the recovery of the brain after any type of injury, whether mechanical or cytotoxic. Also, the release of these immune cells is required for the brain to function normally. A situation of uncontrolled inflammatory response leads to increased damage to the brain. In the mouse model of chemobrain, upregulation of proinflammatory cytokines such as tumor necrosis factor-α (TNF-α), interleukin-6 (IL-6), and anti-inflammatory cytokines IL-4 and IL-10 has been found (Mostofa et al., 2017; Briones and Woods, 2014). As a result of the increase in the inflammatory response, the brain experiences a decrease in the ability to regenerate (neurodegeneration), leakiness of the blood brain barrier, and aberrations in structural integrity of brain tissues (McLeary et al., 2019; Briones and Woods, 2014). The tight junction of the blood brain barrier allows for the protection of the brain, acting as a barrier and preventing the entrance of unwanted molecules and toxins. In the circumstances of chemotherapy treatments, proinflammatory cytokines IL-1, IL-1β, IL-6, and TNF-α have been found to cross the blood brain barrier via the peripheral nervous system (Lomeli et al., 2021). Figure 7 gives a simplified schematic to depict a summary of factors that induce long-term cognitive dysfunction in breast cancer patients. Cyclophosphamide and 5-fluorouracil are agents that pass across the blood brain barrier (BBB) and damage oligodendrocytes and precursor cells (Shi et al., 2019). Methotrexate does not pass the BBB and has been used as a treatment of rheumatoid arthritis, but studies have shown that it indirectly affects the brain in aspects such as immune modulation, alterations in brain structure, decreased memory retention, and executive function (Weiss, 2008; Seigers et al., 2009; Zhang et al., 2009; Janelsins et al., 2010; Wen et al., 2018). Koppelman et al. examined the long-term consequences of CMF treatment and found that patients not only experienced cognitive impairments immediately after treatment but also 20 years later (Koppelmans et al., 2012a). Studies in murine models suggested that increases in inflammation across the different cognitive domains contribute to these long-term impairments (Zhang et al., 2009). Recently a cohort human study found increases in cytokines and chemokines 20 years after chemotherapy treatments, including patients treated with CMF treatments (McAfoose and Baune, 2009; van der Willik et al., 2018; Williams et al., 2018).

### Brain Imaging and Structural Abnormalities in Former Cyclophosphamide, Methotrexate, and Fluorouracil Treated Breast Cancer Patients

With an increase in research on how chemotherapy affects the brain, brain imaging studies play an important role in evaluating the physiological changes in the brain. A hypothesis was developed defining chemotherapy as a cognition disruptor because of its mechanisms of action such as DNA and RNA synthesis interference, microtubule destabilization, and immunosuppression. Some chemotherapeutics can cross the blood brain barrier or indirectly affect the brain by causing inflammation on the peripheral nervous system. *In-vitro* studies on rat neuronal cells show that exposure to cyclophosphamide shrinks the somata and disrupts dendritic and axonal networks (Rzeski et al., 2004). *In vivo* studies in rats given doses of methotrexate, there was a significant decrease in neurons and neuroglial in the CA3 and CA4 regions in the hippocampus which could facilitate memory and learning impairments (Madhyastha et al., 2002). In a study done by Kesler et al. (2009), patients who received types of cancer treatments such as chemotherapy, radiation, and endocrine therapy were evaluated with fMRI. When comparing the chemotherapy treated to control, treated patients had lower prefrontal cortical activation than controls and CMF treated patients had lower activation than TAC treated patients 3 years after treatment (Kesler et al., 2009; Pomykala et al., 2013). A later study done by Kesler et al. (2013) measures the hippocampal volume and cytokine expression. In patients who received cyclophosphamide, fluorouracil, and/or methotrexate therapies had lower left hippocampal volume which can be associated with the expression of IL-6 (Kesler et al., 2013). Methotrexate has been shown to have some anti-inflammatory properties and has been shown to modulate IL-6 and TNFα secretion. One limitation to this study does not specify differences in brain volume and cytokine expression between the different chemotherapy regimens. Studies on how chemotherapy changes matter in the brain are limited and usually made up of small cohorts. The reduction in white matter is a common association of dysfunctions of working memory over 12 months (Ahles and Saykin, 2007). Koppelman and others performed two large cohort studies on individuals who received the CMF regimen roughly 21 years before the study. This study recognizes that with the extended lifespan of survivors of breast cancer, many of the elderly population are at greater risk for experiencing long-term side effects and structural changes in the brain from these treatments. In the current study, participants were in the age range of 50–80 years old. Results of this study showed a significant decrease in TBV and gray matter in the brains of chemotherapy treated patients vs their healthy controls (Koppelmans et al., 2012b). Although the role of gray matter in the mechanism of cognitive dysfunction is unknown, it is known that there is limited long-term recovery. These alterations in brain structure and function post-chemotherapy are surprisingly very similar to changes associated with trauma and neurodegenerative diseases (Pomykala et al., 2013).

### Advantages and Limitations

Using a bibliometric approach is an effective way to mine and depict what information is currently available on a topic of interest. It is versatile, as it can be used with different databases based on the goal to accomplish. However, this methodology does come with some limitations. For example, this method is strictly quantitative and not qualitative and is descriptive and not inferential. Also, only one database can be used at one time. As such, these characteristics should be considered when interpreting data. Although there are limitations, the data presented is impartial and open doors to investigate untouched areas of this top.

Although CMF is one of the oldest therapeutic methods for treating breast cancer and has been replaced by newer and more effective drugs, it is still deemed relevant in science as there are still some unknowns of its long-term effect. This review gives a depiction of continuous developments in CMF and inflammation with a role in cognitive dysfunction. This is achieved by gauging the terminology used in the publications and areas of interest based on journal output. With many advances in immunomodulation in the central nervous system, reevaluations of CMF can open doors to useful information for research of long-term effects of many other drugs, especially chemotherapy that is currently used today.

## References

[B1] Ahles T. A. Saykin A. J. (2007). Candidate Mechanisms for Chemotherapy-Induced Cognitive Changes. Nat. Rev. Cancer. 7, 192–201. 10.1038/nrc2073 17318212 PMC3329763

[B2] Aletaha D. Neogi T. Silman A. J. Funovits J. Felson D. T. Bingham C. O. (2010). 2010 Rheumatoid Arthritis Classification Criteria: an American College of Rheumatology/European League against Rheumatism Collaborative Initiative. Arthritis Rheum. 62, 2569–2581. 10.1002/art.27584 20872595

[B3] Bannwarth B. Labat L. Moride Y. Schaeverbeke T. (1994). Methotrexate in Rheumatoid Arthritis. Drugs. 47, 25–50. 10.2165/00003495-199447010-00003 7510620

[B4] Bedoui Y. Guillot X. Sélambarom J. Guiraud P. Giry C. Jaffar-Bandjee M. C. (2019). Methotrexate an Old Drug With New Tricks. Int. J. Mol Sci. 20, 5023. 10.3390/ijms20205023 PMC6834162 31658782

[B18] Bonadonna G. Brusamolino E. Valagussa P. Rossi A. Brugnatelli L. Brambilla C. (1976). Combination Chemotherapy as an Adjuvant Treatment in Operable Breast Cancer. N. Engl. J. Med. 294 (8), 405–410. 10.1056/NEJM197602192940801 1246307

[B5] Briones T. L. Woods J. (2014). Dysregulation in Myelination Mediated by Persistent Neuroinflammation: Possible Mechanisms in Chemotherapy-Related Cognitive Impairment. Brain Behav. Immun. 35, 23–32. 10.1016/j.bbi.2013.07.175 23916895 PMC3858476

[B6] Christie L-A. Acharya M. M. Parihar V. K. Nguyen V. K. Martirosian V. K. Limoli V. K. (2012). Impaired Cognitive Function and Hippocampal Neurogenesis Following Cancer Chemotherapy. Clin. Cancer Res. 18, 1954–1965. 10.3389/fonc.2020.00147 22338017

[B7] Correa D. D. Ahles T. A. (2008). Neurocognitive Changes in Cancer Survivors. Cancer J. 14, 396–400. 10.1097/ppo.0b013e31818d8769 19060604

[B8] Deprez S. Kesler S. R. Saykin A. J. Silverman D. H. S. de Ruiter M. B. McDonald B. C. (2018). International Cognition and Cancer Task Force Recommendations for Neuroimaging Methods in the Study of Cognitive Impairment in Non-CNS Cancer Patients. J. Natl. Cancer Inst. 110, 223–231. 10.1093/jnci/djx285 29365201 PMC6658857

[B9] Ellegaard O. Wallin J. A. (2015). The Bibliometric Analysis of Scholarly Production: How Great Is the Impact? Scientometrics. 105, 1809–1831. 10.1007/s11192-015-1645-z 26594073 PMC4643120

[B10] Gilman A. Philips F. S. (1946). The Biological Actions and Therapeutic Applications of the B-Chloroethyl Amines and Sulfides. Science. 103, 409–436. 10.1126/science.103.2675.409 17751251

[B11] Heidelberger C. Chaudhuri N. K. Danneberg P. Mooren D. Griesbach L. Duschinsky R. (1957a). Fluorinated Pyrimidines, a New Class of Tumour-Inhibitory Compounds. Nature. 179, 663–666. 10.1038/179663a0 13418758

[B12] Heidelberger C. Leibman K. C. Harbers E. Bhargava P. M. (1957b). The Comparative Utilization of Uracil-2-C14 by Liver, Intestinal Mucosa, and Flexner-Jobling Carcinoma in the Rat. Cancer Res. 17 (5), 399–404. 13437303

[B13] Janelsins M. C. Roscoe J. A. Berg M. J. Thompson B. D. Gallagher M. J. Morrow G. R. (2010). IGF-1 Partially Restores Chemotherapy-Induced Reductions in Neural Cell Proliferation in Adult C57BL/6 Mice. Cancer Invest. 28, 544–553. 10.3109/07357900903405942 20014946 PMC3024545

[B14] Kesler S. Janelsins M. Koovakkattu D. Palesh O. Mustian K. Morrow G. (2013). Reduced Hippocampal Volume and Verbal Memory Performance Associated With Interleukin-6 and Tumor Necrosis Factor-Alpha Levels in Chemotherapy-Treated Breast Cancer Survivors. Brain Behav. Immun. 30, S109–S116. 10.1016/j.bbi.2012.05.017 22698992 PMC3665606

[B15] Kesler S. R. Bennett F. C. Mahaffey M. L. Spiegel D. (2009). Regional Brain Activation during Verbal Declarative Memory in Metastatic Breast Cancer. Clin. Cancer Res. 15, 6665–6673. 10.1158/1078-0432.ccr-09-1227 19843664 PMC2859687

[B16] Koppelmans V. Breteler M. M. B. Boogerd W. Seynaeve C. Gundy C. Schagen S. B. (2012). Neuropsychological Performance in Survivors of Breast Cancer More Than 20 Years After Adjuvant Chemotherapy. J. Clin Oncol. 30, 1080–1086. 10.1200/jco.2011.37.0189 22370315

[B17] Koppelmans V. de Ruiter M. B. van der Lijn F. Boogerd W. Seynaeve C. van der Lugt A. (2012). Global and Focal Brain Volume in Long-Term Breast Cancer Survivors Exposed to Adjuvant Chemotherapy. Breast Cancer Res. Treat. 132, 1099–1106. 10.1007/s10549-011-1888-1 22205140

[B19] Lomeli N. Lepe J. Gupta K. Bota D. A. (2021). Cognitive Complications of Cancer and Cancer-Related Treatments - Novel Paradigms. Neurosci. Lett. 749, 135720. 10.1016/j.neulet.2021.135720 33582187 PMC8423125

[B20] Madhyastha S. Somayaji S. N. Rao M. S. Nalini K. Bairy K. L. (2002). Hippocampal Brain Amines in Methotrexate-Induced Learning and Memory Deficit. Can. J. Physiol. Pharmacol. 80, 1076–1084. 10.1139/y02-135 12489927

[B21] Marks M. Marks J. L. (2016). Viral Arthritis. Clin. Med. 16, 129–134. 10.7861/clinmedicine.16-2-129 PMC4868140 27037381

[B22] McAfoose J. Baune B. T. (2009). Evidence for a Cytokine Model of Cognitive Function. Neurosci. Biobehavioral Rev. 33, 355–366. 10.1016/j.neubiorev.2008.10.005 18996146

[B23] McLeary F. Davis A. Rudrawar S. Perkins A. Anoopkumar-Dukie S. (2019). Mechanisms Underlying Select Chemotherapeutic-Agent-Induced Neuroinflammation and Subsequent Neurodegeneration. Eur. J. Pharmacol. 842, 49–56. 10.1016/j.ejphar.2018.09.034 30287154

[B24] Mokhtari R. B. Homayouni T. S. Baluch N. Morgatskaya E. Kumar S. Das B. (2017). Combination Therapy in Combating Cancer. Oncotarget. 8, 38022–38043. 10.18632/oncotarget.16723 28410237 PMC5514969

[B25] Moore H. C. (2014). An Overview of Chemotherapy-Related Cognitive Dysfunction, or ‘Chemobrain'. Oncology (Williston Park). 28 (9), 797–804. 25224480

[B26] Morrison W. B. (2010). Cancer Chemotherapy: an Annotated History. J. Vet. Intern. Med. 24, 1249–1262. 10.1111/j.1939-1676.2010.0590.x 20840315

[B27] Mostofa A. G. M. Punganuru S. R. Madala H. R. Al-Obaide M. Srivenugopal K. S. (2017). The Process and Regulatory Components of Inflammation in Brain Oncogenesis. Biomolecules. 7, 34. 10.3390/biom7020034 PMC5485723 28346397

[B28] Pomykala K. L. de Ruiter M. B. Deprez S. McDonald B. C. Silverman D. H. S. (2013). Integrating Imaging Findings in Evaluating the Post-Chemotherapy Brain. Brain Imaging Behav. 7, 436–452. 10.1007/s11682-013-9239-y 23828813

[B29] Rzeski W. Pruskil S. Macke A. Felderhoff-Mueser U. Reiher A. K. Hoerster F. (2004). Anticancer Agents Are Potent Neurotoxins *In Vitro* and *In Vivo* . Ann. Neurol. 56, 351–360. 10.1002/ana.20185 15349862

[B30] Seigers R. Schagen S. B. Coppens C. M. van der Most P. J. van Dam F. S. A. M. Koolhaas J. M. (2009). Methotrexate Decreases Hippocampal Cell Proliferation and Induces Memory Deficits in Rats. Behav. Brain Res. 201, 279–284. 10.1016/j.bbr.2009.02.025 19428645

[B31] Shi D.-D. Huang Y.-H. Lai C. S. W. Dong C. M. Ho L. C. Li X.-Y. (2019). Ginsenoside Rg1 Prevents Chemotherapy-Induced Cognitive Impairment: Associations With Microglia-Mediated Cytokines, Neuroinflammation, and Neuroplasticity. Mol. Neurobiol. 56, 5626–5642. 10.1007/s12035-019-1474-9 30659419

[B32] van der Willik K. D. Koppelmans V. Hauptmann M. Compter A. Ikram M. A. Schagen S. B. (2018). Inflammation Markers and Cognitive Performance in Breast Cancer Survivors 20 Years after Completion of Chemotherapy: a Cohort Study. Breast Cancer Res. 20, 135. 10.1186/s13058-018-1062-3 30442190 PMC6238315

[B33] Wefel J. S. Kesler S. R. Noll K. R. Schagen S. B. (2015). Clinical Characteristics, Pathophysiology, and Management of Noncentral Nervous System Cancer-Related Cognitive Impairment in Adults. CA: A Cancer J. Clinicians. 65, 123–138. 10.3322/caac.21258 PMC4355212 25483452

[B34] Weiss B. (2008). Chemobrain: a Translational Challenge for Neurotoxicology. Neurotoxicology. 29, 891–898. 10.1016/j.neuro.2008.03.009 18479752 PMC2583256

[B35] Wen J. Maxwell R. R. Wolf A. J. Spira M. Gulinello M. E. Cole P. D. (2018). Methotrexate Causes Persistent Deficits in Memory and Executive Function in a Juvenile Animal Model. Neuropharmacology. 139, 76–84. 10.1016/j.neuropharm.2018.07.007 29990472 PMC6089371

[B36] Williams A. M. Shah R. Shayne M. Huston A. J. Krebs M. Murray N. (2018). Associations Between Inflammatory Markers and Cognitive Function in Breast Cancer Patients Receiving Chemotherapy. J. Neuroimmunology. 314, 17. 10.1016/j.jneuroim.2017.10.005 29128118 PMC5768199

[B37] Zhang Z. Zhao P. Li A. Lv X. Gao Y. Sun H. (2009). Effects of Methotrexate on Plasma Cytokines and Cardiac Remodeling and Function in Postmyocarditis Rats. Mediators Inflamm. 2009, 389720. 10.1155/2009/389720 19884981 PMC2768010

