# Supplementary material for: A Bibliometric Analysis of Cyclophosphamide, Methotrexate, and Fluorouracil Breast Cancer Treatments: Implication for the Role of Inflammation in Cognitive Dysfunction
Source: Front Mol Biosci. 2021 Aug 20;8:683389. doi: 10.3389/fmolb.2021.683389 (PMC8417522; doi:10.3389/fmolb.2021.683389)
Supplement: Supplementary file 1 [file DataSheet2.PDF]

label	replace by
and	
of	
a	
in	
to	
have	
to	
it	
I	
that	
for	
you	
he	
with	
on	
do	
say	
this	
they	
at	
but	
we	
his	
from	
that	
not	
n't	
by	
she	
or	
as	
what	
go	
their	
can	
who	
get	
if	
would	
her	
all	
my	
make	
about	
know	
will	

as
up
one
time
there
year
so
when
which
them
some
me
people
take
out
into
just
see
him
your
come
could
now
than
like
other
how
then
its
our
two
more
these
want
way
look
first
also
new
because
day
more
use
no
man
find
here

thing
give
many
well
only
those
tell
one
very
her
even
back
any
good
through
us
life
child
there
work
down
may
after
should
call
world
over
school
still
try
in
as
last
ask
need
too
feel
three
when
state
never
become
between
high
really
something
most

another
much
family
own
out
leave
put
old
while
mean
on
keep
student
why
let
great
same
big
group
begin
seem
country
help
talk
where
turn
every
start
hand
might
American
show
part
about
against
place
over
such
again
few
case
most
week
company
where
system
each

right
program
hear
so
question
during
work
play
government
run
small
number
off
always
move
like
night
live
Mr
point
believe
hold
today
bring
happen
next
without
before
large
all
million
must
home
under
water
room
write
mother
area
national
money
story
young
fact
month
different
lot

right
study
book
eye
job
word
though
business
issue
side
kind
four
far
black
long
both
little
house
yes
after
since
long
provide
service
around
friend
important
father
sit
away
until
power
hour
game
often
yet
line
political
end
among
ever
stand
bad
lose
however
member
pay

law
meet
car
city
almost
include
continue
set
later
community
much
name
five
once
white
least
president
real
change
team
minute
best
several
idea
kid
body
information
nothing
ago
right
lead
social
understand
whether
back
watch
together
follow
around
parent
only
stop
face
anything
create
public
already

speak
others
read
level
allow
add
office
spend
door
health
person
art
sure
such
war
history
party
within
grow
result
open
change
morning
walk
reason
low
win
research
girl
guy
early
food
before
moment
himself
air
teacher
force
offer
enough
both
across
although
foot
second
boy
maybe

toward
able
age
off
policy
everything
love
process
music
including
consider
appear
actually
buy
probably
human
wait
serve
market
die
send
expect
home
sense
build
stay
fall
oh
nation
plan
cut
college
interest
death
course
someone
experience
behind
reach
local
kill
six
remain
effect
use
yeah
suggest

class
control
raise
care
perhaps
little
late
hard
field
else
pass
former
sell
major
sometimes
require
along
development
themselves
report
role
better
economic
effort
up
decide
rate
strong
possible
show
leader
light
voice
wife
whole
police
finally
pull
return
free
military
price
report
less
according
decision
explain

son
hope
even
develop
view
relationship
carry
town
road
drive
arm
true
federal
break
better
difference
thank
receive
value
international
building
action
full
join
season
society
because
tax
director
early
position
player
agree
especially
record
pick
wear
paper
special
space
ground
form
support
event
official
whose
matter

everyone
center
couple
site
end
project
hit
base
activity
star
table
need
court
produce
eat
American
oil
half
situation
easy
cost
industry
figure
face
street
image
itself
phone
either
data
cover
quite
picture
clear
practice
piece
land
recent
describe
product
doctor
wall
patient
worker
news
test
movie

certain
north
love
personal
open
support
simply
third
technology
catch
step
baby
computer
type
draw
film
Republican
tree
source
red
nearly
organization
choose
cause
hair
look
point
century
evidence
window
difficult
listen
soon
culture
billion
chance
brother
energy
period
course
summer
less
realize
hundred
available
plant
likely

opportunity
term
short
letter
condition
choice
place
single
rule
daughter
administration
south
husband
Congress
floor
campaign
material
population
well
call
economy
medical
hospital
church
close
thousand
risk
current
fire
future
wrong
involve
defense
anyone
increase
security
bank
myself
certainly
west
sport
board
seek
per
subject
officer
private

rest
deal
fight
throw
top
quickly
past
goal
second
bed
order
author
fill
represent
foreign
drop
plan
upon
agency
push
nature
color
no
recently
store
reduce
sound
note
fine
before
near
movement
page
enter
share
than
common
poor
other
race
concern
series
significant
similar
hot
language
each

usually
response
dead
rise
factor
decade
article
shoot
east
save
seven
artist
away
scene
stock
career
despite
central
eight
thus
treatment
beyond
happy
exactly
approach
lie
size
dog
fund
serious
occur
media
ready
sign
thought
list
individual
simple
quality
pressure
accept
answer
hard
resource
identify
left
meeting

determine
prepare
whatever
success
argue
cup
particularly
amount
ability
staff
recognize
indicate
character
growth
loss
degree
wonder
attack
herself
region
television
box
TV
training
pretty
trade
deal
election
everybody
physical
lay
general
feeling
standard
bill
message
fail
outside
arrive
analysis
benefit
name
sex
forward
lawyer
present
section

environmental
glass
answer
skill
sister
PM
professor
operation
financial
crime
stage
ok
compare
authority
miss
design
sort
one
act
ten
knowledge
gun
station
blue
state
strategy
little
clearly
discuss
indeed
force
truth
song
example
democratic
check
environment
leg
dark
public
various
rather
laugh
guess
executive
set
study

prove
hang
entire
rock
design
enough
forget
since
claim
note
remove
manager
help
close
sound
enjoy
network
legal
religious
cold
form
final
main
science
green
memory
card
above
seat
establish
nice
trial
expert
that
spring
firm
Democrat
radio
visit
management
care
avoid
imagine
tonight
huge
ball
no

close
finish
yourself
talk
theory
impact
respond
statement
maintain
charge
popular
traditional
onto
reveal
direction
weapon
employee
cultural
contain
peace
head
control
base
apply
play
measure
wide
shake
fly
interview
manage
chair
fish
particular
camera
structure
politics
perform
bit
weight
suddenly
discover
candidate
top
production
treat
trip

evening
affect
inside
conference
unit
best
style
adult
worry
range
mention
rather
far
deep
past
edge
individual
specific
writer
trouble
necessary
throughout
challenge
fear
shoulder
institution
middle
sea
dream
bar
beautiful
property
instead
improve
stuff
detail
method
sign
somebody
magazine
hotel
soldier
reflect
heavy
sexual
cause
bag

heat
fall
marriage
tough
sing
surface
purpose
exist
pattern
whom
agent
owner
machine
gas
down
ahead
generation
commercial
address
test
item
reality
coach
step
Mrs
yard
beat
violence
total
tend
investment
discussion
finger
garden
notice
collection
modern
task
partner
positive
civil
kitchen
consumer
shot
budget
wish
painting

scientist
safe
agreement
capital
mouth
nor
victim
newspaper
instead
threat
responsibility
smile
attorney
score
account
interesting
break
audience
rich
dinner
figure
vote
western
relate
travel
debate
prevent
citizen
majority
none
front
born
admit
senior
assume
wind
key
professional
mission
fast
alone
customer
suffer
speech
successful
option
participant

southern
fresh
eventually
no
forest
video
global
Senate
reform
access
restaurant
judge
publish
cost
relation
like
release
own
bird
opinion
credit
critical
corner
concerned
recall
version
stare
safety
effective
neighborhood
original
act
troop
income
directly
hurt
species
immediately
track
basic
strike
hope
sky
freedom
absolutely
plane
nobody

achieve
object
attitude
labor
refer
concept
client
powerful
perfect
nine
therefore
conduct
announce
conversation
examine
touch
please
attend
completely
vote
variety
sleep
turn
involved
investigation
nuclear
researcher
press
conflict
spirit
experience
replace
British
encourage
argument
by
once
camp
feature
afternoon
AM
weekend
dozen
possibility
along
insurance
department

battle
beginning
date
generally
African
very
sorry
crisis
complete
fan
stick
define
easily
through
hole
element
vision
status
normal
Chinese
ship
solution
stone
slowly
scale
bit
university
introduce
driver
attempt
park
spot
lack
ice
boat
drink
sun
front
distance
wood
handle
truck
return
mountain
survey
supposed
tradition

winter
village
Soviet
refuse
sales
roll
communication
run
screen
gain
resident
hide
gold
club
future
farm
potential
increase
middle
European
presence
independent
district
shape
reader
Ms
contract
crowd
Christian
express
apartment
willing
strength
previous
band
obviously
horse
interested
target
prison
ride
guard
terms
demand
reporter
deliver
text

share
tool
wild
vehicle
observe
flight
inside
facility
understanding
average
emerge
advantage
quick
light
leadership
earn
pound
basis
bright
operate
guest
sample
contribute
tiny
block
protection
settle
feed
collect
additional
while
highly
identity
title
mostly
lesson
faith
river
promote
living
present
count
unless
marry
tomorrow
technique
path

ear
shop
folk
order
principle
survive
lift
border
competition
jump
gather
limit
fit
claim
cry
equipment
worth
associate
critic
warm
aspect
result
insist
failure
annual
French
Christmas
comment
responsible
affair
approach
until
procedure
regular
spread
chairman
baseball
soft
ignore
egg
measure
belief
demonstrate
anybody
murder
gift
religion

review
editor
past
engage
coffee
document
speed
cross
influence
anyway
threaten
commit
youth
wave
move
afraid
quarter
background
native
broad
wonderful
deny
apparently
slightly
reaction
twice
suit
perspective
growing
blow
construction
kind
destroy
cook
connection
charge
burn
shoe
view
grade
context
committee
hey
mistake
focus
smile
location

clothes
Indian
quiet
dress
promise
aware
neighbor
complete
drive
active
extend
chief
average
combine
wine
below
cool
voter
mean
demand
bus
hell
dangerous
remind
moral
United
category
relatively
victory
key
academic
visit
Internet
fire
negative
following
historical
medicine
tour
depend
photo
finding
grab
direct
classroom
contact
justice

participate
daily
fair
pair
famous
flower
tape
hire
familiar
appropriate
supply
fully
cut
will
actor
birth
search
tie
democracy
eastern
primary
yesterday
circle
device
progress
next
front
bottom
island
exchange
clean
studio
train
lady
colleague
application
lean
damage
plastic
tall
plate
hate
otherwise
writing
press
start
alive

football
intend
attack
chicken
army
abuse
theater
shut
map
extra
session
danger
welcome
domestic
lots
literature
rain
desire
assessment
injury
respect
northern
nod
paint
fuel
leaf
direct
dry
Russian
instruction
fight
pool
climb
sweet
lead
engine
fourth
salt
expand
importance
metal
fat
ticket
software
disappear
corporate
strange

lip
reading
urban
increasingly
lunch
educational
somewhere
farmer
above
sugar
planet
favorite
explore
obtain
enemy
greatest
complex
surround
athlete
invite
repeat
carefully
soul
scientific
impossible
panel
meaning
mom
married
alone
instrument
predict
weather
presidential
emotional
commitment
Supreme
bear
pocket
thin
temperature
surprise
poll
proposal
consequence
half
breath

sight
cover
balance
adopt
minority
straight
attempt
connect
works
belong
aid
advice
okay
photograph
empty
regional
trail
novel
code
somehow
organize
jury
breast
Iraqi
acknowledge
theme
storm
union
record
desk
fear
thanks
fruit
under
expensive
yellow
conclusion
prime
shadow
struggle
conclude
analyst
dance
limit
like
being
last

ring
largely
shift
revenue
mark
locate
county
appearance
package
difficulty
bridge
recommend
obvious
train
basically
e-mail
generate
anymore
propose
possibly
trend
visitor
loan
currently
comfortable
investor
but
profit
angry
crew
deep
accident
meal
hearing
traffic
muscle
notion
capture
prefer
truly
earth
Japanese
chest
search
thick
cash
museum

beauty
emergency
unique
feature
internal
ethnic
link
content
select
root
nose
declare
outside
appreciate
actual
bottle
hardly
setting
launch
dress
file
sick
outcome
ad
defend
matter
judge
duty
sheet
ought
ensure
Catholic
extremely
extent
component
mix
slow
contrast
zone
wake
challenge
airport
chief
brown
standard
shirt
pilot

warn
ultimately
cat
contribution
capacity
ourselves
estate
guide
circumstance
snow
English
politician
steal
pursue
slip
percentage
meat
funny
neither
soil
influence
correct
Jewish
blame
estimate
due
basketball
late
golf
investigate
crazy
significantly
chain
address
branch
combination
just
frequently
governor
relief
user
dad
kick
part
manner
ancient
silence

rating
golden
motion
German
gender
solve
fee
landscape
used
bowl
equal
long
official
forth
frame
typical
except
conservative
eliminate
host
hall
trust
ocean
score
row
producer
afford
meanwhile
regime
division
confirm
fix
appeal
mirror
tooth
smart
length
entirely
rely
topic
complain
issue
variable
back
range
telephone
perception

attract
confidence
bedroom
secret
debt
rare
his
tank
nurse
coverage
opposition
aside
anywhere
bond
file
pleasure
master
era
requirement
check
stand
fun
expectation
wing
separate
now
clear
struggle
mean
somewhat
pour
stir
judgment
clean
except
beer
English
reference
tear
doubt
grant
seriously
account
minister
totally
hero
industrial

cloud
stretch
winner
volume
travel
seed
surprised
rest
fashion
pepper
separate
busy
intervention
copy
tip
below
cheap
aim
cite
welfare
vegetable
gray
dish
beach
improvement
everywhere
opening
overall
divide
initial
terrible
oppose
contemporary
route
multiple
essential
question
league
criminal
careful
core
upper
rush
necessarily
specifically
tired
rise

tie
employ
holiday
dance
vast
resolution
household
fewer
abortion
apart
witness
match
barely
sector
representative
lack
beneath
beside
black
incident
limited
proud
flow
faculty
increased
waste
merely
mass
emphasize
experiment
definitely
bomb
enormous
tone
liberal
massive
engineer
wheel
invest
promise
cable
towards
expose
rural
AIDS
Jew
narrow

cream
secretary
gate
solid
hill
typically
noise
grass
unfortunately
hat
legislation
succeed
either
celebrate
achievement
fishing
drink
accuse
hand
useful
land
secret
reject
talent
taste
characteristic
milk
escape
cast
sentence
unusual
closely
convince
height
physician
assess
sleep
plenty
ride
virtually
first
addition
sharp
creative
lower
behind
approve

explanation
outside
gay
campus
proper
live
guilty
living
acquire
compete
technical
plus
mind
potential
immigrant
weak
illegal
hi
alternative
interaction
column
personality
signal
curriculum
list
honor
passenger
assistance
forever
fun
regard
Israeli
association
twenty
knock
review
wrap
lab
offer
display
criticism
asset
spiritual
musical
journalist
prayer
suspect

scholar
warning
climate
cheese
observation
childhood
payment
sir
permit
cigarette
definition
priority
bread
creation
graduate
request
emotion
scream
dramatic
universe
gap
excellent
deeply
prosecutor
mark
green
lucky
drag
airline
library
agenda
recover
factory
selection
primarily
roof
unable
expense
initiative
diet
arrest
funding
therapy
wash
schedule
sad
brief

housing
post
purchase
existing
dark
steel
regarding
shout
remaining
visual
fairly
chip
violent
silent
suppose
self
bike
tea
perceive
comparison
settlement
layer
planning
far
description
later
slow
slide
widely
wedding
inform
portion
territory
immediate
opponent
abandon
link
mass
lake
transform
tension
display
leading
bother
consist
alcohol
enable

bend
saving
gain
desert
shall
error
release
cop
Arab
double
walk
sand
Spanish
rule
hit
print
passage
formal
transition
existence
album
participation
arrange
atmosphere
joint
reply
cycle
opposite
lock
whole
deserve
consistent
resistance
discovery
tear
exposure
pose
stream
sale
trust
benefit
pot
grand
mine
hello
coalition
tale

knife
resolve
racial
phase
present
joke
coat
Mexican
symptom
contact
manufacturer
philosophy
potato
interview
foundation
quote
online
pass
negotiation
good
urge
occasion
dust
breathe
elect
investigator
jacket
glad
ordinary
reduction
rarely
shift
pack
suicide
numerous
touch
substance
discipline
elsewhere
iron
practical
moreover
passion
volunteer
implement
essentially
enforcement

vs
sauce
independence
marketing
priest
amazing
intense
advance
employer
shock
inspire
adjust
retire
sure
visible
kiss
illness
cap
habit
competitive
juice
congressional
involvement
dominate
previously
whenever
transfer
analyze
another
attach
for
Indian
disaster
parking
prospect
boss
complaint
championship
coach
exercise
fundamental
severe
enhance
mystery
impose
poverty
other

entry
fat
spending
king
evaluate
symbol
still
trade
maker
mood
accomplish
emphasis
illustrate
boot
monitor
Asian
entertainment
bean
evaluation
creature
commander
digital
arrangement
concentrate
total
usual
anger
psychological
heavily
peak
approximately
increasing
disorder
missile
equally
vary
wire
round
distribution
transportation
holy
ring
twin
command
commission
interpretation
breakfast

stop
strongly
engineering
luck
so-called
constant
race
clinic
veteran
smell
tablespoon
capable
tourist
light
toss
crucial
bury
pray
tomato
exception
butter
deficit
bathroom
objective
block
electronic
ally
journey
reputation
mixture
surely
tower
smoke
confront
pure
glance
dimension
toy
prisoner
fellow
smooth
nearby
peer
designer
personnel
shape
educator

relative
immigration
belt
teaspoon
birthday
implication
perfectly
coast
supporter
accompany
silver
teenager
recognition
retirement
flag
recovery
whisper
watch
gentleman
corn
moon
inner
junior
rather
throat
salary
swing
observer
due
straight
publication
pretty
crop
dig
strike
permanent
plant
phenomenon
anxiety
unlike
wet
literally
resist
convention
embrace
supply
assist

exhibition
construct
viewer
pan
consultant
soon
line
administrator
date
occasionally
mayor
consideration
CEO
secure
pink
smoke
estimate
buck
historic
poem
grandmother
bind
fifth
constantly
enterprise
favor
testing
stomach
apparent
weigh
install
sensitive
suggestion
mail
recipe
reasonable
preparation
wooden
elementary
concert
aggressive
false
intention
channel
extreme
tube
drawing

quit
absence
roll
Latin
rapidly
jail
comment
diversity
honest
Palestinian
pace
employment
speaker
impression
essay
respondent
giant
cake
historian
negotiate
restore
substantial
pop
particular
specialist
origin
approval
mine
quietly
advise
conventional
drop
count
depth
wealth
disability
shell
general
criticize
fast
professional
effectively
biological
pack
onion
deputy
flat

brand
assure
mad
award
criteria
dealer
via
alternative
utility
precisely
arise
armed
nevertheless
highway
clinical
routine
schedule
wage
normally
phrase
ingredient
stake
Muslim
dream
fiber
activist
Islamic
snap
terrorism
refugee
incorporate
hip
ultimate
switch
corporation
valuable
assumption
gear
graduate
minor
provision
killer
assign
gang
developing
classic
chemical

wave
label
teen
index
vacation
advocate
draft
extraordinary
heaven
rough
yell
pregnant
distant
drama
satellite
personally
wonder
clock
chocolate
Italian
Canadian
ceiling
sweep
advertising
universal
spin
house
button
bell
rank
darkness
ahead
clothing
super
yield
fence
portrait
paint
survival
roughly
lawsuit
bottom
testimony
bunch
beat
wind
found

burden
react
chamber
furniture
cooperation
string
ceremony
communicate
taste
cheek
lost
profile
disagree
like
penalty
match
ie
advance
resort
destruction
bear
unlikely
tissue
constitutional
pant
stranger
infection
cabinet
broken
apple
electric
proceed
track
bet
literary
virus
stupid
dispute
fortune
strategic
assistant
overcome
remarkable
occupy
statistics
shopping
cousin

encounter
wipe
initially
blind
white
port
honor
electricity
adviser
pay
spokesman
retain
latter
incentive
slave
chemical
translate
accurate
whereas
terror
though
expansion
elite
Olympic
dirt
odd
rice
bullet
tight
Bible
chart
solar
conservative
process
square
stick
concentration
complicated
gently
champion
scenario
telescope
reflection
revolution
strip
interpret
friendly

tournament
fiction
detect
balance
likely
tremendous
lifetime
recommendation
flow
senator
market
hunting
salad
guarantee
innocent
boundary
pause
remote
satisfaction
journal
bench
lover
raw
awareness
surprising
withdraw
general
deck
similarly
newly
pole
testify
mode
dialogue
imply
naturally
mutual
founder
top
advanced
pride
dismiss
aircraft
delivery
mainly
bake
freeze

platform
finance
sink
attractive
respect
diverse
relevant
ideal
joy
worth
regularly
working
singer
evolve
shooting
partly
unknown
assistant
offense
counter
smell
potentially
transfer
thirty
justify
protest
crash
craft
treaty
terrorist
insight
possess
politically
tap
lie
extensive
episode
double
swim
tire
fault
loose
free
shortly
originally
considerable
prior

intellectual
mix
assault
relax
stair
adventure
external
proof
confident
headquarters
sudden
dirty
violation
tongue
license
hold
shelter
rub
controversy
entrance
favorite
practice
properly
fade
defensive
tragedy
net
characterize
funeral
profession
alter
spot
constitute
establishment
squeeze
imagination
target
mask
convert
comprehensive
prominent
presentation
regardless
easy
load
stable
introduction

appeal
pretend
not
elderly
representation
deer
split
violate
partnership
pollution
emission
steady
vital
neither
fate
earnings
oven
distinction
segment
nowhere
poet
mere
exciting
variation
comfort
adapt
Irish
honey
correspondent
pale
musician
significance
load
round
storage
flee
mm-hmm
leather
distribute
evolution
ill
tribe
shelf
can
grandfather
lawn
buyer

dining
wisdom
council
vulnerable
instance
garlic
capability
poetry
celebrity
gradually
stability
doubt
fantasy
scared
guide
plot
framework
gesture
depending
ongoing
psychology
since
counselor
witness
chapter
fellow
divorce
owe
pipe
athletic
slight
math
shade
tail
sustain
mount
obligation
angle
palm
differ
custom
store
economist
fifteen
soup
celebration
efficient

damage
composition
satisfy
pile
briefly
carbon
closer
consume
scheme
crack
frequency
tobacco
survivor
besides
in
psychologist
wealthy
galaxy
given
fund
ski
limitation
OK
trace
appointment
preference
meter
explosion
arrest
publicly
incredible
fighter
rapid
admission
hunter
educate
painful
friendship
aide
infant
calculate
fifty
rid
porch
tendency
uniform
formation

scholarship
reservation
efficiency
waste
qualify
mall
derive
scandal
PC
helpful
impress
heel
resemble
privacy
fabric
surprise
contest
proportion
guideline
rifle
maintenance
conviction
trick
organic
tent
examination
publisher
strengthen
French
proposed
myth
sophisticated
cow
etc
standing
asleep
tennis
barrel
bombing
membership
ratio
menu
purchase
controversial
desperate
rate
lifestyle

humor
loud
glove
suspect
sufficient
narrative
photographer
helicopter
Catholic
modest
provider
delay
agricultural
explode
stroke
scope
punishment
handful
badly
horizon
curious
downtown
girlfriend
prompt
request
cholesterol
absorb
adjustment
taxpayer
eager
principal
detailed
motivation
assignment
restriction
across
Palestinian
laboratory
workshop
differently
auto
romantic
cotton
motor
sue
flavor
overlook

float
undergo
sequence
demonstration
jet
orange
consumption
assert
blade
temporary
medication
print
cabin
bite
relative
edition
valley
yours
pitch
pine
brilliant
versus
manufacturing
risk
Christian
complex
absolute
chef
discrimination
offensive
German
suit
boom
register
appoint
heritage
God
terrorist
dominant
successfully
shit
lemon
hungry
sense
dry
wander
submit

economics
naked
anticipate
nut
legacy
extension
shrug
fly
battery
arrival
legitimate
orientation
inflation
cope
flame
cluster
host
wound
dependent
shower
institutional
depict
operating
flesh
garage
operator
instructor
collapse
borrow
furthermore
comedy
mortgage
sanction
civilian
twelve
weekly
habitat
grain
brush
consciousness
devote
crack
measurement
province
ease
seize
ethics

nomination
permission
wise
actress
summit
acid
odds
gifted
frustration
medium
function
physically
grant
distinguish
shore
repeatedly
firm
running
correct
distinct
artistic
discourse
basket
ah
fighting
impressive
competitor
ugly
worried
portray
powder
ghost
persuade
moderate
subsequent
continued
cookie
carrier
cooking
frequent
ban
swing
orange
awful
admire
pet
miracle

exceed
rhythm
widespread
killing
lovely
sin
charity
script
tactic
identification
transformation
everyday
headline
crash
venture
invasion
military
nonetheless
adequate
piano
grocery
intensity
exhibit
high
blanket
margin
principal
quarterback
rope
concrete
prescription
African-American
chase
document
brick
recruit
patch
consensus
horror
recording
changing
painter
colonial
pie
sake
gaze
courage

pregnancy
swear
defeat
clue
reinforce
win
confusion
slice
occupation
dear
coal
sacred
criminal
formula
collective
exact
uncle
square
captain
sigh
attribute
dare
okay
homeless
cool
gallery
soccer
defendant
tunnel
fitness
lap
grave
toe
container
virtue
abroad
architect
dramatically
makeup
inquiry
rose
surprisingly
highlight
decrease
indication
rail
anniversary

couch
alliance
hypothesis
boyfriend
compose
peer
mess
rank
legend
regulate
adolescent
shine
norm
upset
remark
resign
reward
gentle
related
organ
lightly
concerning
invent
laughter
fit
northwest
counseling
tight
receiver
ritual
insect
interrupt
salmon
favor
trading
concern
magic
superior
combat
stem
surgeon
acceptable
physics
rape
counsel
brush
jeans

hunt
continuous
log
echo
pill
excited
sculpture
compound
integrate
flour
bitter
bare
slope
rent
presidency
serving
subtle
greatly
bishop
drinking
delay
cry
acceptance
collapse
shop
pump
candy
evil
final
finance
pleased
medal
beg
sponsor
ethical
secondary
slam
export
experimental
melt
midnight
net
curve
integrity
entitle
evident
logic

essence
park
exclude
harsh
closet
suburban
greet
favor
interior
corridor
murder
hay
pitcher
march
snake
pitch
excuse
cross
weakness
pig
cold
classical
estimated
T-shirt
online
unemployment
civilization
fold
patient
pop
daily
reverse
missing
correlation
humanity
flash
developer
reliable
excitement
beef
Islam
Roman
stretch
architecture
occasional
administrative
elbow

deadly
Muslim
Hispanic
allegation
tip
confuse
airplane
monthly
duck
dose
Korean
plead
initiate
lecture
van
sixth
bay
mainstream
suburb
sandwich
unlike
trunk
rumor
implementation
swallow
motivate
render
longtime
trap
restrict
cloth
seemingly
legislative
effectiveness
enforce
lens
reach
inspector
lend
plain
fraud
companion
contend
nail
array
strict
assemble

frankly
burst
hallway
cave
inevitable
southwest
monster
speed
protest
unexpected
obstacle
facilitate
encounter
rip
herb
overwhelming
integration
crystal
recession
wish
top
written
motive
label
flood
pen
ownership
nightmare
notice
inspection
supervisor
consult
arena
laugh
possession
forgive
warm
consistently
basement
project
drift
drain
last
prosecution
maximum
announcement
warrior

prediction
bacteria
questionnaire
mud
infrastructure
hurry
privilege
temple
medium
outdoor
suck
and/or
broadcast
re
leap
random
past
wrist
curtain
monitor
pond
domain
guilt
cattle
subject
walking
playoff
minimum
fiscal
skirt
dump
hence
database
uncomfortable
aim
execute
limb
ideology
average
welcome
tune
continuing
harm
railroad
endure
administer
simultaneously

dancer
amendment
guard
pad
transmission
await
retired
trigger
spill
grateful
grace
virtual
response
colony
adoption
slide
indigenous
closed
convict
civilian
towel
modify
horn
chronic
peaceful
innovation
strain
guitar
replacement
award
glance
prize
landing
conduct
blue
boost
bat
alarm
festival
grip
weird
undermine
freshman
sweat
outer
European
drunk

survey
research
separation
traditionally
stuff
govern
southeast
intelligent
wherever
ballot
rhetoric
convinced
driving
enthusiasm
accommodate
praise
injure
wilderness
nearby
endless
mandate
pause
excuse
respectively
uncertainty
chaos
short
mechanical
canvas
forty
matter
lobby
profound
format
trait
currency
turkey
reserve
beam
abuse
astronomer
corruption
contractor
apologize
doctrine
genuine
thumb

unity
compromise
horrible
exclusive
scatter
commonly
convey
rush
twist
complexity
fork
disk
relieve
suspicion
lock
finish
residence
shame
meaningful
sidewalk
Olympics
technological
signature
pleasant
wow
suspend
rebel
frozen
desire
spouse
fluid
pension
resume
theoretical
sodium
blow
promotion
delicate
forehead
rebuild
bounce
electrical
hook
detective
traveler
click
compensation

signal
exit
attraction
dedicate
altogether
pickup
carve
needle
belly
ship
scare
portfolio
shuttle
invisible
timing
engagement
ankle
transaction
rescue
counterpart
historically
firmly
rider
doll
noon
exhibit
amid
identical
precise
anxious
structural
residential
loud
diagnose
carbohydrate
liberty
poster
theology
nonprofit
crawl
handsome
magic
sum
provided
businessman
promising
conscious

determination
donor
hers
pastor
jazz
opera
Japanese
bite
frame
evil
acquisition
pit
hug
wildlife
punish
giant
primary
equity
wrong
doorway
departure
elevator
teenage
guidance
happiness
statue
pursuit
repair
decent
gym
oral
clerk
Israeli
envelope
reporting
destination
fist
endorse
exploration
generous
bath
rescue
thereby
overall
indicator
sunlight
feedback

spectrum
purple
bold
reluctant
starting
expertise
practically
program
picture
tune
eating
age
volunteer
hint
sharply
parade
advocate
realm
ban
strip
cancel
blend
therapist
slice
peel
pizza
recipient
hesitate
flip
accounting
debate
bias
huh
metaphor
candle
handle
worry
judicial
entity
suffering
full-time
feel
lamp
garbage
servant
addition
regulatory

diplomatic
elegant
inside
reception
vanish
automatically
chin
trail
necessity
confess
racism
starter
interior
banking
casual
gravity
enroll
diminish
Arab
value
minimize
chop
performer
intent
isolate
pump
inventory
productive
assembly
civic
silk
magnitude
steep
hostage
collector
popularity
kiss
alien
dynamic
scary
equation
angel
switch
offering
rage
photography
repair

toilet
disappointed
precious
prohibit
representative
content
realistic
Russian
hidden
command
tender
wake
gathering
outstanding
stumble
lonely
automobile
artificial
dawn
abstract
descend
silly
hook
tide
shared
hopefully
readily
cooperate
revolutionary
romance
hardware
pillow
kit
cook
spread
continent
seal
circuit
sink
ruling
shortage
annually
lately
trap
scan
fool
deadline

rear
processing
ranch
coastal
undertake
softly
reserve
burning
verbal
tribal
ridiculous
automatic
diamond
credibility
import
sexually
spring
way
divine
sentiment
cart
oversee
stem
elder
pro
inspiration
Dutch
quantity
trailer
mate
o'clock
Greek
genius
monument
bid
quest
sacrifice
invitation
accuracy
juror
officially
broker
treasure
loyalty
credit
shock
talented

gasoline
stiff
output
nominee
extended
please
diabetes
slap
toxic
alleged
jaw
grief
mysterious
rocket
donate
inmate
tackle
dynamics
bow
ours
senior
dignity
carpet
parental
bubble
heat
buddy
barn
sword
flash
seventh
glory
tightly
tuck
drum
faint
post
queen
dilemma
input
specialize
northeast
shallow
liability
sail
merchant
stadium

improved
bloody
defeat
associated
withdrawal
refrigerator
nest
near
thoroughly
lane
ancestor
condemn
steam
accent
escape
optimistic
unite
cage
equip
shrimp
homeland
exchange
rack
costume
wolf
courtroom
statute
cartoon
besides
productivity
grin
symbolic
seal
bug
bless
aunt
agriculture
rock
hostile
root
conceive
combined
instantly
bankruptcy
vaccine
bonus
collaboration

mixed
opposed
orbit
grasp
patience
spite
tropical
voting
patrol
willingness
position
revelation
rent
calm
jewelry
Cuban
haul
concede
trace
wagon
afterward
spectacular
ruin
sheer
prior
reliability
ass
alongside
bush
exotic
fascinating
secure
clip
thigh
bull
drawer
regard
sheep
discourage
coordinator
ideological
runner
secular
intimate
empire
cab
divorce

exam
documentary
neutral
biology
flexible
progressive
web
conspiracy
catch
casualty
republic
execution
terrific
whale
functional
star
draft
instinct
teammate
aluminum
whoever
ministry
verdict
instruct
skull
self-esteem
ease
cooperative
manipulate
bee
practitioner
loop
edit
whip
puzzle
mushroom
subsidy
boil
tragic
mathematics
mechanic
jar
respect
earthquake
pork
creativity
safely

underlying
dessert
sympathy
fisherman
incredibly
isolation
sock
near
jump
eleven
sexy
entrepreneur
bureau
seat
workplace
ambition
touchdown
utilize
breeze
costly
ambitious
Christianity
presumably
influential
translation
uncertain
dissolve
object
statistical
metropolitan
rolling
aesthetic
spell
insert
booth
helmet
waist
expected
lion
accomplishment
royal
panic
cast
crush
actively
cliff
minimal

cord
fortunately
cocaine
illusion
anonymous
tolerate
appreciation
commissioner
harm
flexibility
instructional
scramble
casino
decorate
sort
charge
pulse
equivalent
fixed
experienced
donation
diary
sibling
irony
spoon
midst
alley
upset
interact
soap
cute
rival
short-term
punch
pin
hockey
passing
persist
supplier
known
momentum
purse
shed
liquid
icon
elephant
consequently

legislature
associate
franchise
correctly
mentally
foster
bicycle
encouraging
cheat
access
heal
fever
filter
rabbit
coin
exploit
accessible
organism
sensation
partially
stay
upstairs
dried
minimum
pro
conservation
shove
backyard
charter
stove
consent
comprise
reminder
alike
placement
dough
grandchild
dam
reportedly
well-known
surrounding
ecological
outfit
unprecedented
columnist
workout
preliminary

patent
shy
quote
trash
disabled
gross
damn
texture
counter
pencil
associate
frontier
spray
bet
disclose
custody
banker
beast
interfere
oak
case
eighth
notebook
outline
gaze
attendance
speculation
uncover
behalf
innovative
shark
reward
mill
installation
stimulate
tag
vertical
swimming
fleet
catalog
outsider
sacrifice
desperately
stance
compel
sensitivity
someday

instant
debut
proclaim
worldwide
hike
required
confrontation
colorful
ideal
constitution
trainer
Thanksgiving
scent
stack
eyebrow
sack
cease
inherit
tray
pioneer
organizational
textbook
uh
nasty
shrink
model
emerging
dot
wheat
fierce
envision
rational
kingdom
aisle
weaken
protocol
exclusively
vocal
marketplace
openly
unfair
terrain
deploy
risky
pasta
genre
distract

merit
planner
chunk
closest
discount
no
ladder
jungle
migration
breathing
invade
hurricane
retailer
classify
wound
coup
aid
ambassador
density
supportive
curiosity
skip
aggression
stimulus
journalism
robot
flood
dip
likewise
informal
Persian
feather
sphere
tighten
boast
pat
perceived
sole
publicity
major
unfold
joke
well-being
validity
ecosystem
strictly
partial

collar
weed
compliance
streak
supposedly
added
builder
glimpse
premise
specialty
deem
artifact
sneak
monkey
mentor
two-thirds
listener
lightning
legally
sleeve
disappointment
disturb
rib
excessive
high-tech
debris
pile
rod
logical
liberal
ash
socially
parish
slavery
blank
commodity
cure
mineral
hunger
dying
developmental
faster
spare
halfway
cure
equality
cemetery

harassment
deliberately
fame
regret
striking
likelihood
carrot
atop
toll
rim
embarrassed
fucking
cling
isolated
blink
suspicious
wheelchair
squad
eligible
processor
plunge
this
sponsor
grin
color
demographic
rain
chill
refuge
steer
legislator
rally
programming
cheer
outlet
intact
vendor
thrive
peanut
chew
elaborate
intellectual
conception
auction
steak
comply
triumph

shareholder
comparable
transport
conscience
calculation
considerably
interval
scratch
awake
jurisdiction
inevitably
feminist
constraint
emotionally
expedition
allegedly
compromise
strain
similarity
butt
lid
dumb
bulk
sprinkle
mortality
philosophical
conversion
patron
municipal
any
liver
harmony
solely
tolerance
instant
goat
arm
blessing
banana
running
palace
formerly
peasant
neat
grandparent
lawmaker
supermarket

cruise
mobile
plain
part
calendar
widow
deposit
beard
brake
downtown
screening
impulse
forbid
fur
brutal
predator
poke
opt
voluntary
trouble
valid
forum
dancing
happily
soar
removal
autonomy
enact
round
thread
light
landmark
unhappy
offender
coming
privately
fraction
distinctive
tourism
threshold
calm
routinely
suite
remark
straw
theological
apart

exhaust
globe
fragile
objection
chemistry
old-fashioned
crowded
circle
blast
prevail
overnight
denial
rental
fantastic
fragment
level
screw
warmth
undergraduate
liquid
headache
policeman
yield
projection
battle
suitable
mention
graduation
drill
cruel
mansion
regard
grape
authorize
cottage
driveway
charm
sexuality
loyal
clay
pound
balloon
invention
ego
fare
homework
disc

sofa
guarantee
availability
radar
frown
regain
leave
permit
sweater
rehabilitation
rubber
retreat
freely
favorable
steadily
veteran
integrated
ha
youngster
broadcast
premium
accountability
overwhelm
one-third
contemplate
update
spark
ironically
beyond
speculate
marker
low
preach
bucket
bomb
blond
confession
provoke
marble
substantially
twist
defender
fish
explicit
transport
disturbing
surveillance

magnetic
technician
mutter
devastating
depart
arrow
trauma
neighboring
soak
ribbon
meantime
transmit
screen
harvest
consecutive
republican
coordinate
worldwide
within
spy
slot
riot
citizenship
severely
sovereignty
ridge
brave
lighting
specify
contributor
frustrate
crowd
articulate
importantly
transit
dense
seminar
electronics
sunny
shorts
swell
accusation
soften
photograph
straighten
terribly
cue

sudden
bride
biography
hazard
compelling
seldom
tile
economically
honestly
troubled
bow
twentieth
balanced
foreigner
launch
convenience
delight
weave
timber
till
accurately
plea
bulb
copy
flying
sustainable
devil
bolt
cargo
seller
skilled
managing
public
marine
dock
organized
diplomat
boring
sometime
summary
missionary
epidemic
fatal
trim
warehouse
accelerate
butterfly

bronze	
drown	
inherent	
praise	
nationwide	
spit	
harvest	
kneel	
vacuum	
selected	
dictate	
stereotype	
sensor	
laundry	
manual	
pistol	
naval	
plaintiff	
kid	
middle-class	
apology	
till	
binding	
alpha	
beta	
conclusion	
method	
result	
anthracycline	anthracyclines
anticancer	anti-cancer
anticancer drug	anticancer drugs
antioxidant enzyme	antioxidant enzymes
antioxidant	antioxidants
antitumor activity	antitumor-activity
breast cancer	breast-cancer
breast cancer cells	breast-cancer cells
cancer stem cells	cancer stem-cells
cancer cells	cancer-cells
carcinoma cells	carcinoma-cells
cardiac myocytes	cardiomyocytes
cardiac toxicity	cardiotoxicity
caspase	caspases
cell	cells
cell cycle	cell-cycle
cell cycle arrest	cell-cycle arrest
cell death	cell-death
cell proliferation	cell-proliferation

cell-line	cell-lines
colon cancer	colon-cancer
colorectal cancer	colorectal-cancer
complex	complexes
dna damage	dna-damage
dna repair	dna-repair
doxorubicin-induced cardiotoxicity	doxorubicin cardiotoxicity
drug	drugs
drug delivery	drug-delivery
drug resistance	drug-resistance
epithelial-mesenchymal transition	emt
endoplasmic reticulum stress	endoplasmic-reticulum stress
estrogen receptor	estrogen-receptor
extract	extracts
free radicals	free-radicals
gastric cancer	gastric-cancer
gene expression	gene-expression
gene	genes
glutathione peroxidase	glutathione-peroxidase
heart failure	heart-failure
hepatocellular carcinoma	hepatocellular-carcinoma
hydrogen peroxide	hydrogen-peroxide
induce apoptosis	induced apoptosis
induced toxicity	induced cytotoxicity
inhibitor	inhibitors
ischemia-reperfusion	ischemia-reperfusion injury
l.	
ligand	ligands
line	lines
lipid peroxidation	lipid-peroxidation
liposome	liposomes
lung cancer	lung-cancer
manganese superoxide dismutase	mnsod
manganese-superoxide-dismutase	mnsod
manganese superoxide-dismutase	mnsod
mapk	map kinase
mcf-7	mcf-7 cells
mda-mb-231	mda-mb-231 cells
mechanism	mechanisms
melanoma	melanoma-cells
microrna	micrornas
mitochondria	mitochondrial
mouse	mouse model
multidrug resistance	multidrug-resistance
mutation	mutations
nanoparticle	nanoparticles
nf-kappa b	nf-kappa-b

nitric oxide	nitric-oxide
ovarian cancer	ovarian-cancer
oxygen species generation	oxygen species production
p38	p38 mapk
pancreatic cancer	pancreatic-cancer
pathway	pathways
permeability transition	permeability transition pore
phase-ii	phase-ii trial
photosensitizer	photosensitizers
polymorphism	polymorphisms
prostate cancer	prostate-cancer
protective role	protects
protection	protects
protein	proteins
radiation therapy	radiation-therapy
radiotherapy	radiation-therapy
rat	rats
reactive oxygen species	reactive oxygen species (ros)
receptor	receptors
review	
ros generation	ros production
signal transduction	signal-transduction
signaling pathway	signaling pathways
strategy	strategies
superoxide anion	superoxide
superoxide dismutase	superoxide-dismutase
suppresses	suppression
target	targets
tissue	tissues
tnf-alpha	tnf alpha
trail	
transcription factor	transcription factors
tumor	tumors
vitamin c	vitamin-c
vitamin e	vitamin-e
brain	
chemobrain	chemo-brain
cognition	
cognitive	
clinical	
clinical trial	clinical-trial
human trial	human-trial
metaanylsis	meta-analysis
