# Supplementary material for: A Bibliometric Analysis of Cyclophosphamide, Methotrexate, and Fluorouracil Breast Cancer Treatments: Implication for the Role of Inflammation in Cognitive Dysfunction
Source: Front Mol Biosci. 2021 Aug 20;8:683389. doi: 10.3389/fmolb.2021.683389 (PMC8417522; doi:10.3389/fmolb.2021.683389)
Supplement: Supplementary file 2 [file DataSheet4.PDF]

id	label	cluster	Links	Total link strength
389	1st-line treatment	5	170	393
1173	5-fluorouracil	5	454	1798
1186	5-fu	5	130	221
1628	accumulation	4	257	568
1678	acid	4	358	1228
1830	activated protein-kinase	2	338	1083
1840	activated receptor-gamma	2	133	228
1850	activated-receptor-gamma	2	119	195
1867	activation	2	836	11237
1969	acute kidney injury	4	111	232
1977	acute lung injury	3	94	168
1981	acute lymphoblastic-leukemia	1	166	286
1987	acute myeloid leukemia	2	123	203
1989	acute myeloid-leukemia	2	267	652
2006	acute promyelocytic leukemia	2	122	221
2049	adalimumab	1	124	391
2101	adenocarcinoma	5	327	983
2117	adenosine	1	142	263
2162	adhesion	3	178	425
2184	adipocytes	3	135	286
2195	adipokines	1	144	393
2196	adiponectin	1	202	621
2226	adipose-tissue	1	315	1168
2231	adiposity	1	104	217
2245	adjuvant chemotherapy	5	285	758
2262	adjuvant-induced arthritis	1	101	199
2300	adriamycin	4	194	442
2336	adults	1	121	175
2416	aerobic exercise	1	86	214
2419	aerobic glycolysis	2	147	296
2478	age	1	144	219
2495	agent	4	155	314
2502	agents	4	273	733
2527	aging	1	142	248
2613	akt	2	310	1099
2648	albumin	5	184	490
2677	alcohol	1	121	194
3047	alzheimers-disease	4	180	332
3086	american-college	1	125	299
3177	ampk	2	209	506
3249	analogs	4	117	236
3283	anca-associated vasculitis	1	62	147
3303	androgen receptor	2	171	320
3324	anemia	1	134	221
3348	angiogenesis	3	676	4437
3444	ankylosing-spondylitis	1	99	183

3474	antagonist	3	109	163
3508	anthracyclines	4	148	295
3544	anti-cancer	4	360	1426
3601	anti-inflammation	4	161	286
3603	anti-inflammatory	4	174	331
3747	antibacterial activity	4	96	182
3766	antibodies	1	138	226
3769	antibody	5	206	373
3792	anticancer activity	4	242	698
3794	anticancer agents	4	117	234
3808	anticancer drugs	4	237	573
3879	antigen	3	116	196
4013	antioxidant activity	4	233	581
4029	antioxidant enzymes	2	204	452
4047	antioxidant status	2	151	326
4056	antioxidants	4	531	3299
4117	antitumor	4	225	528
4135	antitumor immunity	3	166	378
4140	antitumor necrosis factor	1	85	159
4149	antitumor-activity	4	390	1308
4270	apoptosis	2	860	21242
4444	aromatase	1	118	264
4448	aromatase expression	1	140	310
4468	arrest	2	176	466
4480	arsenic trioxide	2	166	392
4495	artemisinin	4	103	212
4532	arthritis	1	273	722
4574	aryl-hydrocarbon receptor	2	127	216
4648	aspirin	1	177	397
4657	assay	4	230	647
4673	association	1	555	2594
4686	asthma	3	128	194
4738	atherosclerosis	1	259	913
4786	atp	3	131	230
4890	autoantibodies	1	110	190
4928	autoimmunity	1	204	376
4963	autophagy	2	575	4136
5036	azathioprine	1	86	173
5079	b-cell lymphoma	1	95	138
5083	b-cells	3	145	244
5136	bacteria	4	126	233
5266	bax	2	162	402
5304	bcl-2	2	238	760
5310	bcl-2 family	2	116	218
5454	berberine	4	148	264
5511	beta-catenin	2	197	456
5572	bevacizumab	5	184	425

5727	bioavailability	4	134	214
5818	biological evaluation	4	169	476
5843	biologics	1	97	190
5847	biology	2	163	306
5852	biomarker	5	377	1227
5857	biomarkers	1	483	1532
5903	biosynthesis	4	114	176
5981	bladder	3	100	166
5984	bladder cancer	5	211	412
6001	bladder-cancer	5	170	297
6034	blockade	3	138	252
6041	blood	1	207	385
6079	blood-brain-barrier	3	129	217
6164	body mass index	1	129	286
6170	body-composition	1	102	245
6175	body-mass index	1	344	1475
6192	bone	1	168	305
6230	bone metastasis	3	126	241
6252	bone-marrow	3	272	612
6265	bone-marrow-transplantation	1	120	179
6287	bortezomib	2	136	242
6418	brca1	2	119	206
6437	breast	1	272	582
6519	breast neoplasms	1	132	224
6537	breast-cancer	3	952	33701
6540	breast-cancer cells	2	663	5378
6547	breast-cancer metastasis	3	187	389
6550	breast-cancer patients	1	225	405
6555	breast-cancer risk	1	196	386
6558	breast-cancer survivors	1	102	234
6790	c-myc	2	204	456
6797	c-reactive protein	1	518	3155
6888	cachexia	1	247	745
6938	calcium	2	218	444
7044	cancer	4	934	13362
7055	cancer cachexia	1	115	226
7082	cancer chemotherapy	4	145	235
7125	cancer metastasis	3	123	196
7137	cancer patients	5	122	189
7140	cancer prevention	1	152	268
7154	cancer risk	1	120	227
7161	cancer stem cell	2	158	272
7168	cancer stem-cells	2	333	1072
7190	cancer therapy	4	261	659
7203	cancer-associated fibroblasts	3	159	375
7225	cancer-cells	2	616	4281
7226	cancer-chemotherapy	4	146	271

7237	cancer-patients	5	242	484
7242	cancer-related fatigue	1	103	241
7243	cancer-related inflammation	5	188	340
7259	cancer-therapy	4	281	925
7326	capecitabine	5	132	217
7388	carbon nanotubes	4	95	180
7418	carboplatin	5	142	248
7445	carcinogenesis	2	425	1390
7453	carcinoma	5	709	5146
7461	carcinoma in-situ	3	127	203
7486	carcinoma-cells	2	383	1618
7488	carcinomas	3	118	183
7559	cardiomyocytes	4	112	227
7561	cardiomyopathy	4	111	240
7571	cardiotoxicity	4	281	873
7578	cardiovascular disease	1	197	466
7585	cardiovascular events	1	94	170
7598	cardiovascular risk	1	116	255
7607	cardiovascular-disease	1	262	815
7700	caspase activation	2	112	221
7714	caspase-3	4	203	417
7742	caspases	2	230	700
7760	catalase	2	179	376
7892	ccl2	3	131	283
8009	cd4(+) t-cells	3	127	191
8022	cd44	2	164	277
8138	celecoxib	3	146	281
8158	cell carcinoma	5	132	196
8205	cell invasion	3	131	215
8211	cell lung-cancer	5	448	1522
8227	cell migration	3	150	311
8298	cell-cycle	2	334	1234
8299	cell-cycle arrest	2	332	1381
8307	cell-death	2	484	2089
8323	cell-growth	2	175	297
8330	cell-lines	2	249	498
8337	cell-migration	3	186	383
8340	cell-proliferation	2	382	1177
8347	cell-survival	2	111	181
8355	cells	4	839	9449
8358	cells in-vitro	2	107	166
8405	cellular senescence	2	145	294
8414	cellular uptake	4	121	261
8444	central-nervous-system	3	194	382
8517	cervical cancer	5	190	381
8677	chemistry	4	112	180
8710	chemokine	3	179	338

8741	chemokine receptors	3	118	194
8744	chemokines	3	245	614
8749	chemoprevention	2	305	895
8769	chemoradiotherapy	5	163	346
8777	chemoresistance	2	322	1020
8780	chemosensitivity	2	146	283
8806	chemotherapy	5	912	15919
8827	chemotherapy resistance	2	128	212
8892	childhood	1	86	128
8914	children	1	219	485
8961	chitosan	4	84	146
9004	chloroquine	2	117	222
9011	cholangiocarcinoma	5	183	305
9021	cholesterol	1	143	252
9170	chronic inflammation	3	378	946
9188	chronic lymphocytic-leukemia	2	151	255
9281	cigarette-smoking	1	128	224
9356	circulating tumor-cells	3	137	233
9380	cisplatin	4	531	3255
9393	cisplatin resistance	2	136	268
9457	classification	1	280	710
9481	cleavage	2	114	197
9537	clinical remission	1	71	190
9555	clinical trials	1	130	182
9573	clinical-practice guidelines	5	117	224
9579	clinical-trial	1	191	319
9580	clinical-trials	1	157	262
9674	co-delivery	4	132	343
9759	cognitive impairment	1	101	174
9767	cohort	1	193	464
9801	colitis	1	156	308
9810	collagen	1	123	183
9825	collagen-induced arthritis	1	257	858
9847	colon	5	202	348
9864	colon-cancer	5	543	2516
9865	colon-cancer cells	2	120	216
9892	colony-stimulating factor	3	234	509
9920	colorectal-cancer	5	746	6371
9933	combination	4	524	2117
9944	combination therapy	4	327	928
9982	comet assay	4	125	237
10081	complexes	4	294	739
10084	complications	1	170	329
10263	constituents	4	109	225
10264	constitutive activation	2	135	253
10272	consumption	1	118	186
10321	contributes	3	212	426

10339	controlled-release	4	106	257
10340	controlled-trial	1	185	335
10394	copper	4	159	425
10508	coronary-heart-disease	1	164	406
10536	corticosteroids	1	96	179
10612	cox-2	3	279	747
10710	criteria	1	163	353
10729	crohn's disease	1	103	189
10732	crohns-disease	1	198	418
10749	cross-talk	3	157	278
10758	crp	1	144	262
10802	crystal-structure	4	108	187
10906	curative resection	5	145	536
10916	curcumin	4	373	1398
11032	cycle	2	189	461
11033	cycle arrest	2	320	1523
11061	cyclin d1	2	147	275
11097	cyclooxygenase	3	127	215
11108	cyclooxygenase-2	3	310	862
11112	cyclooxygenase-2 expression	3	152	289
11133	cyclophosphamide	1	470	1927
11143	cyclophosphamide-induced cystitis	3	97	239
11243	cystitis	3	102	247
11276	cytochrome-c	2	225	656
11278	cytochrome-c release	2	114	238
11304	cytokine	3	386	1016
11328	cytokine production	3	160	284
11352	cytokines	1	623	3023
11454	cytotoxicity	4	461	2877
11533	damage	4	397	1502
11639	death	2	543	4252
11752	degradation	2	227	532
11799	delivery	4	358	1802
11858	dendritic cells	3	412	1563
11956	depression	1	212	778
11969	derivatives	4	225	768
12005	design	4	185	517
12062	dexamethasone	1	173	331
12108	diabetes	1	173	335
12112	diabetes-mellitus	1	175	317
12134	diagnosis	1	371	1119
12209	diet	1	175	462
12310	differential expression	3	122	180
12331	differentiation	3	500	2169
12506	discovery	4	156	277
12510	disease	1	657	3443
12544	disease-activity	1	214	697

12568	diseases	1	181	289
12694	dna	4	319	877
12740	dna methylation	2	177	396
12788	dna-binding	4	167	332
12796	dna-damage	2	579	3576
12800	dna-damage response	2	155	291
12811	dna-repair	2	222	474
12832	docetaxel	5	276	645
12842	docosahexaenoic acid	1	147	299
12946	double-blind	1	468	2171
12952	double-strand breaks	2	119	220
12959	down-regulation	2	509	2412
12974	doxorubicin	4	545	3212
13066	drug discovery	4	167	299
13120	drug-delivery	4	395	2292
13156	drug-resistance	2	472	2354
13167	drugs	4	387	1305
13220	ductal adenocarcinoma	5	136	291
13285	dysfunction	1	283	621
13316	e-cadherin	2	182	461
13387	early rheumatoid-arthritis	1	98	186
13507	efficacy	1	466	1773
13539	egfr	5	223	527
13584	eicosapentaenoic acid	1	103	212
13605	elderly-patients	5	104	171
13783	emt	2	506	3143
13873	endometrial cancer	1	171	377
13912	endoplasmic-reticulum	2	157	298
13913	endoplasmic-reticulum stress	2	321	1213
13939	endothelial cells	3	140	230
13943	endothelial dysfunction	1	132	243
13949	endothelial growth-factor	3	521	2177
13972	endothelial-cells	3	375	1134
14190	enzymes	4	162	283
14242	epidemiology	1	301	787
14254	epidermal-growth-factor	2	181	334
14291	epigenetics	2	170	327
14328	epithelial ovarian-cancer	5	157	260
14345	epithelial-cells	2	402	1257
14386	epstein-barr-virus	1	147	239
14405	er stress	2	218	722
14451	erk	2	186	468
14529	escherichia-coli	4	163	282
14543	esophageal cancer	5	170	318
14588	estradiol	1	107	170
14597	estrogen	1	304	919
14634	estrogen-receptor	2	406	1203

14642	estrogen-receptor-alpha	2	217	493
14657	etanercept	1	153	451
14755	events	1	91	196
14812	exercise	1	210	689
14854	exosomes	3	140	237
14908	exposure	4	186	386
14920	expression	3	937	18627
14971	extracellular matrix	3	144	233
14992	extracellular-matrix	3	229	572
15008	extracts	4	275	907
15202	factor-alpha	1	187	356
15221	factor-i	3	166	284
15225	factor-kappa-b	2	396	1091
15247	failure	1	100	159
15270	family	2	198	490
15352	fatigue	1	247	1084
15377	fatty-acid synthase	2	114	200
15379	fatty-acids	1	173	307
15424	features	1	96	151
15518	ferroptosis	2	155	313
15599	fibroblast-like synoviocytes	1	100	192
15601	fibroblasts	3	309	879
15617	fibrosis	3	329	779
15683	fish-oil	1	89	186
15732	flavonoids	4	263	829
15801	fluorescence	4	115	195
15856	fluorouracil	5	234	616
15874	focal adhesion kinase	2	118	174
15953	follow-up	1	223	467
16124	free-radicals	2	367	1141
16534	gastric-cancer	5	497	2315
16620	gefitinib	5	137	280
16657	gemcitabine	5	322	993
16682	gene delivery	4	85	151
16731	gene-expression	2	733	4848
16776	generation	4	325	1044
16777	genes	2	541	2238
16801	genetic polymorphisms	1	106	187
16824	genetics	1	114	189
16829	genistein	2	144	287
16847	genome-wide association	1	175	294
16873	genotoxicity	4	137	307
17005	glasgow prognostic score	5	120	386
17037	glioblastoma	2	280	765
17047	glioma	2	175	457
17057	glioma-cells	2	140	272
17117	glucocorticoids	1	192	344

17148	glucose-metabolism	2	148	277
17203	glutathione	4	380	1635
17231	glutathione-peroxidase	2	148	299
17241	glutathione-s-transferase	2	151	236
17257	glycation end-products	1	149	258
17290	glycolysis	2	215	573
17372	gold nanoparticles	4	197	496
17521	graphene oxide	4	114	275
17548	green tea	2	129	219
17595	growth	2	770	7171
17617	growth-factor	3	451	1605
17622	growth-factor receptor	2	311	834
17627	growth-factor-beta	3	267	628
17631	growth-factor-i	1	185	461
17637	growth-inhibition	2	155	254
17706	guidelines	1	188	298
17735	gut microbiota	1	196	397
17960	head	5	274	665
17962	head and neck cancer	5	120	210
17973	health	1	232	562
18023	heart	1	136	213
18034	heart-disease	1	99	160
18036	heart-failure	1	210	490
18104	helicobacter-pylori	3	112	201
18187	heme oxygenase-1	2	194	411
18333	hepatocellular-carcinoma	5	594	2928
18335	hepatocellular-carcinoma cells	2	110	183
18361	hepatotoxicity	4	199	652
18377	her2	5	146	263
18451	heterogeneity	4	169	263
18498	hif-1-alpha	3	128	246
18563	high-dose chemotherapy	1	110	152
18703	histone deacetylase inhibitors	2	117	196
18794	hmgb1	3	153	345
18860	homeostasis	2	164	308
19114	human breast-cancer	2	384	1345
19207	human hepatocellular-carcinoma	2	130	207
19340	human prostate-cancer	2	139	223
19519	hydrogen-peroxide	4	434	1928
19580	hyperalgesia	3	80	143
19643	hyperthermia	4	117	228
19695	hypoxia	4	446	1645
19718	hypoxia-inducible factor-1-alpha	2	136	245
19803	identification	2	513	2081
19845	ifn-gamma	3	206	438
19939	il-1 beta	3	160	275
19965	il-17	3	128	271

20011	il-6	3	360	1153
20123	immune	3	191	325
20134	immune cells	3	193	433
20182	immune response	3	198	335
20188	immune suppression	3	123	266
20191	immune system	3	148	252
20219	immune-response	3	252	546
20221	immune-responses	3	213	434
20224	immune-system	3	214	420
20226	immunity	3	386	1182
20287	immunohistochemistry	3	173	321
20316	immunomodulation	3	178	322
20357	immunosuppression	3	239	522
20373	immunotherapy	3	454	1712
20381	impact	5	320	877
20459	in-situ	4	123	205
20471	in-vitro	4	823	8927
20495	in-vivo	4	717	4223
20510	inactivation	4	147	305
20513	inadequate response	1	115	421
20579	index	5	163	321
20610	indocyanine green	4	80	201
20657	induced apoptosis	2	512	3084
20672	induced cardiomyopathy	4	92	205
20674	induced cardiotoxicity	4	141	301
20687	induced cytotoxicity	4	104	205
20775	induced nephrotoxicity	4	136	400
20788	induced oxidative stress	4	231	560
20860	induction	2	596	3798
20878	infection	1	340	793
20881	infections	1	88	136
20899	infiltrating lymphocytes	3	107	209
20906	infiltration	3	193	431
20916	inflammasome	3	124	186
20919	inflammation	1	964	34363
20970	inflammatory bowel disease	1	171	333
20986	inflammatory cytokines	1	189	333
21030	inflammatory response	3	219	431
21032	inflammatory responses	3	131	196
21039	inflammatory-bowel-disease	1	288	676
21044	infliximab	1	196	612
21105	inhibition	2	759	7102
21147	inhibitors	2	520	2192
21181	inhibits proliferation	2	116	221
21211	injury	4	331	1079
21215	innate	3	133	234
21231	innate immunity	3	246	462

21283	insulin	1	196	500
21289	insulin resistance	1	147	359
21312	insulin-resistance	1	357	1389
21414	interferon-alpha	1	124	209
21421	interferon-gamma	3	182	329
21454	interleukin-1	3	134	240
21469	interleukin-10	3	119	198
21485	interleukin-17	3	110	231
21519	interleukin-6	1	502	1949
21534	interleukin-8	3	179	362
21585	interstitial cystitis	3	105	296
21607	intervention	1	128	240
21637	intestinal inflammation	1	119	182
21649	intestinal mucositis	5	111	299
21882	invasion	3	476	2564
21913	invasiveness	3	139	274
21933	involvement	1	373	1076
21951	ionizing radiation	2	134	238
21953	ionizing-radiation	2	256	645
21965	ipilimumab	5	106	228
22000	irinotecan	5	189	422
22009	iron	2	221	469
22056	irradiation	5	229	478
22085	ischemia-reperfusion injury	4	162	300
22305	jnk	2	197	568
22319	joint damage	1	71	157
22364	juvenile idiopathic arthritis	1	107	271
22422	kappa-b	2	419	1246
22423	kappa-b activation	3	184	309
22481	keratinocytes	4	126	218
22524	kidney	4	114	276
22560	kinase	2	380	1312
23050	leptin	1	228	699
23065	lesions	1	82	123
23090	leucovorin	5	125	302
23093	leukemia	2	318	769
23107	leukemia-cells	2	203	501
23219	life-span	2	124	201
23254	ligands	2	188	398
23345	lines	2	259	816
23409	lipid-peroxidation	4	354	1259
23443	lipopolysaccharide	3	186	394
23472	liposomes	4	196	527
23508	liver	4	289	709
23557	liver-cancer	2	122	190
23643	localization	4	130	209
23711	long-term survivors	1	99	188

23795	low-dose methotrexate	1	119	242
23900	lung	3	272	597
23931	lung metastasis	3	149	268
23946	lung-cancer	5	686	3814
24026	lymph-node metastasis	3	156	355
24035	lymphangiogenesis	3	150	368
24063	lymphocyte	5	131	293
24073	lymphocyte ratio	5	202	625
24090	lymphocytes	3	327	812
24128	lymphoma	1	218	437
24265	macrophage	3	257	606
24292	macrophage polarization	3	121	232
24305	macrophages	3	551	2452
24340	magnetic resonance imaging	1	128	249
24373	maintenance	1	133	209
24429	malignancy	1	127	237
24473	malnutrition	1	106	203
24501	mammalian target	2	152	268
24508	mammalian-cells	2	109	157
24523	mammary epithelial-cells	2	124	211
24526	mammary gland	1	129	226
24550	mammary-gland	2	119	191
24564	mammary-tumors	3	111	209
24574	management	1	388	1225
24625	map kinase	2	263	789
24689	marker	5	218	484
24694	markers	1	336	1030
24744	mast-cells	3	159	295
24810	matrix metalloproteinases	3	303	825
24874	mcf-7 cells	2	331	1333
24909	mda-mb-231 cells	2	164	374
24940	mdsc	3	120	226
24977	mechanisms	4	829	8291
25002	mediated apoptosis	2	188	457
25105	mediterranean diet	1	131	244
25165	melanoma-cells	3	411	1330
25168	melatonin	4	262	700
25190	membrane	4	135	232
25241	men	1	114	200
25297	mesenchymal stem cells	3	130	213
25301	mesenchymal stem-cells	3	231	500
25307	mesenchymal transition	2	240	628
25321	mesoporous silica nanoparticles	4	118	321
25337	messenger-rna	2	308	750
25339	messenger-rna expression	3	131	199
25352	meta-analysis	5	204	607
25354	metaanalysis	1	347	1337

25388	metabolic syndrome	1	329	1149
25402	metabolism	2	563	2611
25484	metastases	5	150	252
25487	metastasis	3	718	6936
25508	metastatic breast-cancer	2	219	408
25514	metastatic colorectal-cancer	5	133	253
25524	metastatic melanoma	3	125	203
25543	metformin	2	269	724
25572	methotrexate	1	588	5021
25609	methylation	2	172	395
25615	methylene-blue	4	69	141
25680	mice	3	572	2661
25690	micelles	4	161	479
25708	microarray	2	124	199
25729	microbiota	1	167	312
25751	microenvironment	3	430	1675
25761	microglia	3	120	210
25823	micrornas	2	329	1078
25824	microsatellite instability	5	98	151
25829	microscopic polyangiitis	1	40	96
25907	migration	3	464	2170
26066	mirna	2	142	225
26131	mitochondrial	2	582	3771
26173	mitochondrial dysfunction	2	317	879
26197	mitochondrial membrane potential	2	148	337
26211	mitochondrial pathway	2	121	224
26214	mitochondrial permeability transition	2	113	203
26290	mitomycin-c	5	88	143
26359	mmp-9	3	115	213
26380	mnsod	2	241	640
26400	model	3	475	1469
26408	models	3	149	228
26441	modifying antirheumatic drugs	1	160	515
26449	modulation	2	331	913
26477	molecular docking	4	103	178
26542	molecular-mechanisms	2	433	1236
26612	monoclonal-antibody	1	247	532
26629	monocyte chemoattractant protein-1	3	175	378
26646	monocytes	3	197	418
26702	mortality	1	375	1402
26758	mouse model	3	455	1564
26759	mouse models	3	122	201
26799	mri	1	124	219
26861	mtor	2	193	469
26935	mucositis	5	208	566
26968	multicenter	1	251	610
26994	multidrug-resistance	4	344	1433

27054	multiple myeloma	2	143	238
27064	multiple-myeloma	2	202	386
27070	multiple-sclerosis	1	160	264
27124	murine model	3	184	330
27141	muscle	1	118	198
27213	mutations	2	324	856
27257	mycophenolate-mofetil	1	99	234
27295	myeloid cells	3	216	572
27310	myeloid-derived suppressor cells	3	152	299
27346	myocardial-infarction	1	207	555
27401	n-acetylcysteine	4	130	256
27495	nadph oxidase	2	224	531
27548	nanocarriers	4	138	369
27594	nanomaterials	4	95	199
27595	nanomedicine	4	212	604
27605	nanoparticles	4	445	3180
27645	nanotechnology	4	110	192
27686	nasopharyngeal carcinoma	5	178	306
27736	natural-killer-cells	3	153	301
27740	natural-products	4	194	528
27796	neck-cancer	5	220	491
27801	necroptosis	2	164	334
27803	necrosis	2	220	610
27809	necrosis-factor	3	144	234
27815	necrosis-factor-alpha	1	512	1809
27848	negative breast-cancer	3	178	313
27876	neoadjuvant chemotherapy	5	258	715
27907	neoplasms	1	131	214
27936	nephrotoxicity	4	166	554
27947	nerve growth-factor	3	60	131
28067	neuroinflammation	3	124	225
28105	neurons	3	102	199
28112	neuropathic pain	3	151	299
28137	neurotoxicity	4	132	268
28168	neutrophil	5	281	934
28191	neutrophil to lymphocyte ratio	5	96	233
28197	neutrophil-lymphocyte ratio	5	185	654
28204	neutrophil-to-lymphocyte ratio	5	151	552
28210	neutrophil/lymphocyte ratio	5	159	607
28224	neutrophils	3	325	799
28249	nf kappa b	3	152	267
28288	nf-kappa-b	2	813	9219
28416	nitric-oxide	4	495	1871
28427	nitric-oxide synthase	3	371	996
28457	nivolumab	5	119	305
28463	nk cells	3	129	229
28484	nlr	5	121	346

28486	nlrp3 inflammasome	3	170	297
28588	non-hodgkins-lymphoma	1	91	132
28613	non-small cell lung cancer	5	264	614
28621	non-small-cell lung cancer	5	137	220
28720	nonsteroidal antiinflammatory drugs	1	353	934
28848	nrf2	2	325	1122
28873	nsaids	1	155	296
28878	nsclc	5	187	345
28926	nuclear factor-kappa b	3	136	252
29044	nutrition	1	163	328
29103	obesity	1	483	2818
29206	of-the-literature	1	149	227
29235	older-adults	1	91	170
29352	oncology	1	191	306
29412	open-label	5	184	459
29516	oral mucositis	5	140	344
29700	osteoarthritis	1	112	179
29740	osteopontin	1	129	230
29742	osteoporosis	1	137	250
29751	osteosarcoma	2	207	504
29775	outcomes	5	308	1022
29824	ovarian-cancer	2	541	2363
29843	overall survival	5	181	523
29855	overexpression	2	307	889
29861	overweight	1	97	196
29871	oxaliplatin	5	293	828
29901	oxidation	4	109	158
29911	oxidative damage	2	166	323
29916	oxidative dna-damage	2	136	230
29929	oxidative stress	2	873	14890
29989	oxygen	4	206	491
30003	oxygen species production	2	141	267
30028	p-glycoprotein	4	272	844
30071	p21	2	109	229
30107	p38 mapk	2	241	627
30131	p53	2	434	2229
30204	paclitaxel	4	394	1383
30236	pain	3	266	655
30335	pancreatic ductal adenocarcinoma	5	128	236
30349	pancreatic-cancer	5	538	2467
30553	pathogenesis	1	399	1283
30593	pathways	2	686	5927
30594	patient	1	127	205
30620	patterns	1	166	314
30672	pd-l1	5	138	222
30832	peptide	4	125	220
30880	performance	1	116	204

30985	peripheral-blood	3	217	427
31035	permeability	3	145	280
31039	permeability transition pore	2	145	313
31124	pet	1	131	198
31162	ph	4	121	248
31210	pharmacokinetics	4	271	625
31232	pharmacology	3	116	162
31272	phase-i	5	221	439
31281	phase-ii trial	5	411	1221
31283	phase-iii	5	229	456
31284	phase-iii trial	5	169	320
31327	phenotype	3	267	530
31473	phosphorylation	2	400	1603
31543	photodynamic therapy	4	403	2404
31586	photosensitizers	4	171	647
31599	photothermal therapy	4	157	651
31632	physical activity	1	133	370
31644	physical-activity	1	273	1025
31677	phytochemicals	2	139	257
31697	pi3k	2	137	262
31704	pi3k/akt	2	126	257
31795	piperlongumine	2	111	223
31840	placebo	1	128	328
31844	placebo-controlled trial	1	178	407
31899	plasma	1	227	467
31999	platelet	5	144	368
32040	platelets	5	121	202
32045	platform	4	77	172
32133	plus methotrexate	1	89	214
32187	polarization	3	122	214
32218	poly(adp-ribose) polymerase	2	130	206
32351	polymeric micelles	4	89	180
32356	polymeric nanoparticles	4	123	238
32369	polymorphisms	1	343	1050
32404	polyphenols	2	181	401
32431	polysaccharides	4	95	175
32443	polyunsaturated fatty-acids	1	209	455
32470	poor-prognosis	5	310	991
32472	population	1	236	544
32540	positron-emission-tomography	1	168	348
32583	postmenopausal women	1	276	963
32647	potent	4	101	166
32721	ppar-gamma	2	237	552
32802	prediction	1	136	226
32827	predictor	5	156	375
32829	predictors	1	137	222
32830	predicts	5	104	242

32840	predicts survival	5	94	209
32855	pregnancy	1	131	214
32906	preoperative neutrophil	5	117	316
32966	pretreatment neutrophil	5	144	518
32970	prevalence	1	239	633
32973	prevention	1	466	1705
33135	probiotics	1	96	201
33152	prodrug	4	120	276
33175	progenitor cells	3	157	298
33190	prognosis	5	561	3993
33198	prognostic factor	5	178	446
33209	prognostic marker	5	139	267
33221	prognostic score	5	122	302
33227	prognostic value	5	227	479
33229	prognostic-factor	5	194	436
33230	prognostic-factors	5	182	305
33231	prognostic-significance	5	269	612
33254	progression	3	635	4056
33274	proinflammatory cytokines	1	159	255
33293	proliferation	2	672	5135
33365	promotes	3	242	611
33454	prostaglandin e-2	3	172	334
33489	prostate	3	189	355
33506	prostate-cancer	2	699	4239
33507	prostate-cancer cells	2	218	489
33546	proteasome	2	124	213
33574	protects	4	279	940
33707	protein-kinase	2	195	454
33720	protein-kinase-c	2	151	310
33754	proteins	2	668	3629
33777	proteomics	2	174	310
33895	psoriasis	1	187	464
33900	psoriatic arthritis	1	91	247
33945	pten	2	135	249
34199	quality of life	1	120	295
34203	quality-of-life	1	349	1520
34204	quantification	1	102	154
34228	quantum dots	4	112	241
34236	quercetin	4	189	491
34341	radiation	5	361	1006
34403	radiation-therapy	5	558	2461
34450	radiographic progression	1	97	263
34476	radioresistance	2	136	283
34477	radiosensitivity	2	120	222
34536	randomized controlled-trial	1	266	747
34552	randomized-trial	1	234	458
34639	rat model	3	177	311

34669	ratio	5	125	322
34678	rats	4	452	1915
34733	reactive oxygen	2	367	1388
34738	reactive oxygen species (ros)	2	743	8325
34927	receptors	3	630	3139
34935	recognition	3	119	199
34961	recommendations	1	193	422
34972	recruitment	3	182	408
34976	rectal cancer	5	102	175
34981	recurrence	5	312	1033
35037	redox	2	165	315
35066	redox regulation	2	206	475
35121	reduction	4	157	271
35164	regeneration	1	109	175
35225	regulator	2	135	219
35247	regulatory t cells	3	132	232
35250	regulatory t-cells	3	420	1629
35282	release	4	302	1010
35296	remission	1	114	321
35349	renal-cell carcinoma	5	143	256
35362	repair	2	213	470
35444	resection	5	202	731
35462	resistance	2	648	4302
35491	resistin	1	90	230
35565	responses	3	343	792
35597	resveratrol	2	325	943
35635	retinoic acid	2	121	203
35745	rheumatoid arthritis	1	438	2830
35756	rheumatoid-arthritis	1	539	2838
35879	risk	1	643	4046
35885	risk factors	1	105	192
35901	risk-factors	1	367	1035
35904	rituximab	1	172	427
35974	roles	2	238	574
35994	ros	2	639	6424
36015	ros production	4	218	548
36231	safety	1	264	665
36347	sarcopenia	1	117	275
36470	score	5	141	376
36566	secretion	3	125	207
36649	selenium	4	216	497
36714	self-renewal	2	203	531
36740	senescence	2	233	618
36772	sensitivity	2	250	611
36815	sepsis	3	122	178
36896	serum	1	235	532
37000	serum-albumin	5	121	245

37006	serum-levels	1	182	354
37256	signal transducer	2	109	193
37269	signal-transduction	2	269	691
37293	signaling pathways	2	562	3305
37319	silica nanoparticles	4	92	165
37336	silver nanoparticles	4	126	300
37410	singlet oxygen	4	119	285
37435	sirt1	2	113	197
37478	size	4	113	201
37509	skeletal-muscle	1	204	479
37521	skin	1	227	427
37712	smooth-muscle-cells	2	199	415
37841	solid tumors	5	231	479
37923	sonodynamic therapy	4	104	290
37934	sorafenib	2	133	246
38061	sphingosine-1-phosphate	3	98	168
38086	spinal-cord	3	93	166
38181	squamous-cell carcinoma	5	363	1031
38222	stability	4	123	181
38233	stage	5	173	351
38288	staphylococcus-aureus	4	88	139
38303	stat3	2	356	1167
38339	statins	1	127	188
38344	statistics	5	311	964
38376	stem cells	2	151	229
38388	stem-cell transplantation	1	148	255
38389	stem-cells	2	503	2171
38525	strategies	4	209	443
38546	stress	2	546	2395
38589	stroma	3	133	264
38601	stromal cells	3	207	415
38605	stromal fibroblasts	3	104	241
38747	subtypes	2	144	256
38776	sulfasalazine	2	114	198
38854	superoxide	4	211	542
38867	superoxide-dismutase	2	310	911
38886	supplementation	1	193	413
38911	suppression	2	368	1143
38931	suppressor-cells	3	351	1219
38995	surgery	5	297	925
39025	survival	5	735	7781
39051	survivors	1	137	451
39054	susceptibility	1	241	608
39137	symptoms	1	140	407
39206	synovial-fluid	1	104	171
39213	synovitis	1	92	261
39265	system	4	346	896

39295	systemic inflammation	5	269	913
39300	systemic inflammatory response	5	133	400
39309	systemic lupus erythematosus	1	95	141
39323	systemic-lupus-erythematosus	1	209	423
39325	systems	4	119	237
39368	t-cell	3	178	280
39396	t-cells	3	494	2061
39487	tamoxifen	1	264	670
39553	targeted delivery	4	133	248
39582	targeted therapy	5	213	405
39628	targets	2	408	1363
39743	temozolomide	2	161	302
39794	term-follow-up	1	104	219
39913	tgf-beta	3	379	1341
39945	th17 cells	3	148	332
40052	therapeutic target	2	281	682
40063	therapeutics	4	182	330
40066	therapy	1	871	7529
40135	thioredoxin	2	151	276
40144	thioredoxin reductase	2	172	377
40215	thrombosis	1	95	179
40247	thymoquinone	4	118	204
40433	tissues	1	353	920
40459	tlr4	3	177	342
40494	tnf alpha	3	557	2353
40545	to-lymphocyte ratio	5	154	433
40546	to-mesenchymal transition	2	139	247
40561	tocilizumab	1	139	329
40573	tolerance	3	125	236
40594	toll-like receptors	3	255	588
40728	toxicity	4	476	2202
40789	traditional chinese medicine	4	146	241
40808	trail-induced apoptosis	2	109	194
40849	transcription	2	299	902
40867	transcription factor nrf2	2	104	193
40880	transcription factors	2	386	1257
40902	transcriptional regulation	2	112	177
40959	transformation	2	138	268
40985	transgenic mice	3	161	264
41008	transition	2	143	265
41058	transplantation	1	183	304
41063	transport	4	108	191
41067	transporter	2	142	298
41085	trastuzumab	5	169	308
41117	treatment	1	269	541
41175	trial	1	372	1081
41268	triple-negative breast cancer	3	184	325

41403	tuberculosis	3	96	140
41447	tumor angiogenesis	3	198	424
41502	tumor hypoxia	4	116	218
41539	tumor microenvironment	3	461	2007
41565	tumor necrosis factor-alpha	3	156	271
41581	tumor progression	3	310	892
41614	tumor stroma	3	124	258
41648	tumor-associated macrophages	3	382	1596
41669	tumor-cells	2	433	1611
41677	tumor-growth	2	503	2250
41688	tumor-infiltrating lymphocytes	5	223	581
41702	tumor-metastasis	3	121	232
41706	tumor-necrosis-factor	1	507	1921
41731	tumor-suppressor	2	237	627
41737	tumor-suppressor gene	2	105	183
41754	tumorigenesis	2	393	1250
41761	tumors	5	661	3461
41896	tyrosine kinase	2	139	234
41900	tyrosine kinase inhibitor	5	134	232
41984	ulcerative colitis	1	112	177
41986	ulcerative-colitis	1	205	472
42007	ultrasound	4	133	354
42066	unfolded protein response	2	200	516
42087	united-states	1	144	269
42117	up-conversion nanoparticles	4	68	149
42123	up-regulation	2	536	2486
42203	urinary-bladder	3	117	239
42236	urothelial carcinoma	5	131	250
42312	uveitis	1	129	359
42365	validation	1	197	422
42400	variants	1	96	181
42482	vasculitis	1	99	218
42521	vegf	3	261	689
42584	versus-host-disease	1	109	161
42746	vitamin d	1	141	254
42772	vitamin-c	4	129	234
42776	vitamin-d	1	150	224
42782	vitamin-e	4	166	347
42819	vivo	4	211	591
42955	wegeners-granulomatosis	1	69	203
42966	weight-loss	1	198	632
43109	women	1	405	1646
43133	wound healing	5	105	168
43342	zinc	4	136	238

Occurrences	Avg. pub. year	Avg. citations	Avg. norm. citations
50	2016.2	16.8	0.8237
239	2016.1055	13.1046	0.7041
28	2015.6429	13.0714	0.6247
70	2015.5143	44.3143	1.6219
162	2016.179	12.1173	0.7127
141	2014.8369	32.844	1.3271
30	2014.7333	31.0333	1.6187
30	2013.6	20.7333	0.6488
1423	2015.3289	20.5348	0.8861
32	2016.1562	25.5938	1.4516
26	2016.3462	24	1.069
46	2015.3478	18.3913	0.9442
29	2016.1379	12.4483	0.6189
104	2015.5962	36.0288	1.4463
29	2014.4138	31.9655	0.9505
55	2015.5273	18.1091	0.8808
129	2015.4341	24.5504	0.9961
36	2014.5556	16.7778	0.6956
59	2014.7966	19.3898	0.6783
29	2015.4483	25.069	1.2125
43	2015.3333	19.5814	0.9474
74	2015.7432	17.5135	1.0874
156	2015.4423	27.0128	1.1827
26	2015.6154	15.1538	0.8555
102	2015.8911	20.5784	0.8899
28	2016	11.0714	0.6048
52	2015.4423	13.3846	0.6594
26	2015.2308	16.8846	0.7349
27	2014.9259	34.3333	0.9809
37	2013.8919	47.9189	1.3778
31	2015.8065	16.9032	0.7437
41	2016.0244	16.6341	1.0108
102	2015.7353	14.8627	0.9855
33	2014.7576	31.8788	1.1555
118	2015.265	18.7542	0.8326
55	2015.537	20.2545	0.8696
26	2015.0769	11.8462	0.5224
54	2015.4815	28.1667	1.2451
46	2015.587	49.7826	1.3136
55	2016.1091	23.1273	1.069
36	2016.25	10.4167	0.6367
29	2014.5862	13.9655	0.5712
44	2015.1136	28.2045	1.1199
35	2014.7714	13.5429	0.5003
542	2015.0684	23.0351	0.9587
29	2014.5172	29.2069	0.9816

27	2014.8077	30.1481	1.0403
36	2015.25	42.5278	1.595
190	2016.4603	16.5526	1.0038
39	2015.9487	13.2051	0.8021
48	2015.4792	18.6458	0.7421
25	2017.16	17.6	1.1854
38	2014.9737	23.4737	0.8734
54	2015.2593	45.7037	1.7905
95	2016.5895	11.8737	0.8737
38	2015.6579	20.5526	0.9743
77	2015.7532	20.3506	1.3041
26	2015	23.3462	1.1092
80	2015.7875	22.3125	1.1163
49	2014.0204	20.4898	0.7168
38	2013.9737	20.4474	0.959
417	2015.6091	22.3429	0.9464
74	2015.6892	13.8378	0.693
46	2015.7609	40.4348	2.0633
25	2014.84	28.04	1.332
173	2015.4855	21.2023	0.9877
2638	2015.6905	17.2271	0.8309
34	2015.3235	13.9412	0.666
42	2015.7381	23.619	1.0911
54	2015.8704	12.7593	0.7406
44	2014.5	21.6591	0.7635
27	2015.8519	37.037	1.5448
109	2014.7103	16.1284	0.5776
31	2014.9677	31.7742	1.3137
50	2015.46	19.56	0.9881
90	2015.8764	13.1333	0.6771
350	2015.5201	16.7343	0.7953
33	2014.5758	27.4545	1.0347
128	2015.3438	25.7266	1.1038
29	2015.069	26.5517	1.1308
34	2014.7353	17.1176	0.6234
55	2014.9455	20.1455	0.8262
491	2016.3663	21.0061	1.0838
25	2015.04	8.88	0.3914
26	2015.5385	16.3077	0.8656
34	2014.6471	36.0882	1.5247
35	2015.5882	17.9143	0.8205
49	2014.9388	16.7143	0.5937
86	2014.6279	19.3488	0.7045
25	2014.96	19.52	0.8565
29	2016.8276	17.1034	1.111
61	2015.2833	27.3115	1.0938
55	2015.2727	17.2364	0.7477

28	2016.6071	25	1.1486
71	2016.7324	11.9155	0.7842
26	2015.7692	23.8462	0.9508
41	2015.3659	21.5854	0.8234
155	2015.8516	19.9935	1.0042
198	2016.1327	16.7323	0.9203
30	2015.8	29.3	1.4456
26	2014.5	14	0.4837
51	2015.6275	13.3529	0.6212
39	2015.7179	11.8718	0.6605
33	2016.3333	18.8182	1.4557
59	2015.4576	12	0.6126
33	2015.4062	25.3636	1.3119
31	2015.7419	24.4516	1.0246
32	2015.4062	34.5938	1.2745
187	2015.615	35.2781	1.5358
45	2015.3778	21.4444	0.9741
29	2014.931	36.1379	1.3692
96	2014.6562	60.1979	1.9943
36	2014.4167	23.1111	0.8271
32	2014.8125	14.25	0.6092
26	2015.0769	20.3846	0.8819
84	2014.7738	18.2143	0.747
37	2014.9189	14.5676	0.5191
4695	2015.4106	23.3129	0.9962
802	2014.9788	28.3504	1.1472
60	2015.6102	38.5833	2.0357
64	2015.2812	27.7969	1.2669
70	2015.0143	28.1143	1.0799
38	2015.8649	28.7632	1.4102
57	2014.8947	29.2632	1.3836
434	2015.261	28.03	1.2692
98	2015.6082	19.7143	0.9365
61	2015.8333	23.623	0.9839
1829	2015.5479	25.3762	1.1622
31	2015.5161	31.8387	1.364
32	2014.6562	19.5312	0.9427
29	2014.5862	35.2414	1.3242
27	2015.0769	22.8519	0.7556
31	2014.9032	39.871	1.4654
29	2015.6897	22.7931	1.0073
29	2015.6897	31.6207	1.2062
132	2016.3561	23.5985	1.3112
88	2016.1023	25.5682	1.6154
41	2015.1707	30.4634	1.1995
551	2015.5145	19.7804	0.9017
42	2014.6429	48.3571	1.7227

69	2015.3478	19.7536	0.8641
29	2015.8276	16.4138	0.8205
42	2015.8095	19.3571	0.8235
145	2016.5241	21.6966	1.7041
27	2015.2222	14.1481	0.5699
25	2015.48	36.72	1.7669
33	2015.5455	9.5758	0.4529
171	2014.7661	20.1053	0.8139
659	2015.1942	17.698	0.7776
28	2014.6429	37	1.301
197	2014.7716	33.6091	1.2665
25	2013.7917	27.84	0.784
26	2015.5769	21.1154	0.9393
31	2016.2258	21.6452	1.1739
106	2016.0755	19.783	1.0068
59	2016.0339	26.3051	1.2375
26	2015.0385	32.6154	1.3419
33	2014.9394	19	0.6927
120	2015.6417	17.7583	1.049
25	2013.96	21.2	0.7353
44	2015.8636	15.6364	0.7755
83	2014.6265	18.1205	0.662
45	2014.8864	20	0.8459
31	2015.8387	23.4516	1.0798
29	2014.0357	65.6207	1.8829
33	2015.9375	23.4545	1.3709
33	2016.0606	11.6061	0.7292
28	2015.5357	16.7143	1.3963
29	2015.4828	17	0.9393
215	2015.4419	24.5814	1.1558
39	2015.8718	15.3077	0.7841
146	2015.4521	16.0753	0.7562
167	2015.7844	23.6467	1.1946
271	2015.7786	24.9004	1.0624
37	2015.3514	27.9189	1.1306
68	2015.5441	19.0294	0.9342
49	2016.0204	29.6735	1.3363
151	2015.2649	23.8477	0.9641
25	2015.32	29.68	1.1797
1294	2015.8298	18.0595	0.9124
27	2015.8889	22.6296	1.1956
40	2015.7	25.65	1.1367
37	2015.4054	33.973	1.3174
67	2015.1343	26.5373	1.1031
47	2015.9362	14.2128	0.83
31	2016.129	14.5806	0.9555
42	2014.6667	47.2857	1.5074

27	2014.5185	25.2222	0.7463
74	2014.8919	28	0.9984
109	2014.5185	27.422	0.968
45	2015.9091	15.9556	0.8328
112	2016.1712	19.3482	0.9973
30	2015.2333	16.9	0.7399
2162	2015.9569	17.7368	0.9818
25	2016.32	18.24	1.2067
25	2014.24	16.68	0.5873
92	2015.2717	14.163	0.5703
25	2015.92	15.2	0.7498
27	2016.5556	37.8519	1.6324
39	2015.8158	14.5385	0.6901
35	2015.2286	17.8286	0.7122
126	2015.4048	36.4206	1.3155
40	2015	51.4	2.0066
29	2014.8276	13.5517	0.6064
33	2015.9394	37.8485	1.9298
398	2015.9849	16.8769	0.9727
33	2016.6364	13.5455	0.9803
110	2015.5364	18.9636	0.8091
26	2015.44	17.8846	0.6577
31	2014.6129	26.2258	0.8845
25	2015.96	47.72	1.9671
30	2016.1333	15	0.8122
46	2014.8667	24.3696	1.0483
40	2015.175	25.325	0.9391
45	2017.7778	17.1556	1.7861
25	2016.4583	16.32	0.9432
66	2015.0462	18.7121	0.7123
42	2016.3571	14.2857	1.0587
26	2015.3462	27.6923	1.1655
118	2015.2373	21.9576	0.9428
46	2015.8913	20.8478	1.1782
309	2015.288	24.8123	1.083
28	2014.5	33.4643	1.0899
78	2014.9744	41.6795	2.0631
812	2015.7219	24.447	1.1177
271	2016.3296	17.3948	1.0869
121	2016.4667	18.0744	1.0305
30	2014.9333	15.9	0.6339
114	2015.7699	17.4561	0.8188
55	2015	11.6727	0.6456
34	2014.6176	18.4706	0.6456
31	2015.5806	24.0968	1.1582
26	2014.6	13.4231	0.5174
54	2015.6604	21.9259	1.0491

38	2017.0526	25.8158	2.057
52	2014.7885	21.0385	0.939
55	2015.1818	21.6182	0.8966
65	2014.8308	39.6462	1.7036
33	2014.7273	10.6061	0.4707
87	2014.9425	14.1149	0.7874
56	2015.2143	20.0714	0.7637
26	2014.2308	17.9615	0.7615
64	2015.1562	18.6562	0.7968
33	2016.0909	23.2727	0.9224
33	2015.5938	12.1818	0.5797
37	2016.1351	31.4595	1.6848
65	2015.0794	36.7077	1.3153
159	2015.7987	25.3082	1.3495
59	2015.4746	12.8983	0.7141
183	2015.4809	18.8415	0.9265
35	2014.5714	22.2571	0.7236
26	2014.8846	32.8462	1.0596
107	2014.5421	20.1121	0.754
39	2014.8718	17.9487	0.6843
310	2015.2395	14.6677	0.7124
45	2013.7111	17.7111	0.5373
40	2014.125	12.65	0.5182
80	2014.6375	23.125	0.902
29	2014.0345	24.931	0.8605
131	2015.2672	19.8244	0.8133
37	2014.8611	23.8919	1.0103
369	2015.4755	19.5176	0.8923
391	2015.7969	16.6573	0.8118
200	2015.6162	15.33	0.6993
518	2015.0833	21.1873	0.8472
77	2015.5455	23.4935	1.0334
255	2017.004	17.3765	1.3684
226	2015.0398	35.2522	1.4332
106	2015.0952	20.934	0.8502
117	2016.3077	14.7436	0.8697
78	2017.3718	12.5	1.2598
45	2015.8222	13.4667	0.6705
41	2015.7317	21.3659	0.9855
39	2015.4872	24.4615	0.8665
201	2015.3781	13.3483	0.6472
59	2015.5085	19.4576	0.8518
30	2015.3	20	0.9256
293	2015.0515	22.0717	0.8596
41	2016	15.9268	0.9393
514	2015.0953	19.0214	0.8816
110	2014.8182	38.7273	1.153

45	2015.0444	12.5556	0.6057
126	2015.552	18.0476	0.8006
62	2015.6885	23.0806	0.847
50	2015.76	15.86	0.7354
456	2015.567	24.636	0.9789
47	2015.7447	27.6596	1.4672
64	2015.5938	16.6094	0.748
86	2015.8837	17.2093	1.0426
43	2015.1429	24.7209	0.9518
343	2015.0906	25.3965	1.04
34	2015.2059	17.3235	1.0217
299	2015.6074	24.913	1.2636
403	2016.1712	20.3623	1.097
42	2016.1667	12.1429	0.6733
332	2016.6265	20.9217	1.5238
284	2015.912	20.419	1.0768
186	2016.0486	15.2043	0.8638
39	2016.5641	26.9744	1.5198
82	2015.9146	22.8171	1.0825
59	2015.0847	33.1695	1.3336
28	2013.8214	28.6429	0.7924
262	2015.9228	12.1069	0.776
67	2015.8806	12.3582	0.7158
30	2015.3571	21.4667	0.9083
25	2015.52	20.28	1.2238
385	2015.9143	26.6494	1.3363
50	2015.56	22.52	0.9799
39	2015.4103	21.6154	0.9253
149	2015.6376	26.2685	1.2303
26	2015.2308	19.2308	0.7465
33	2016.6061	8.3939	0.5789
289	2014.7163	30.9758	1.2214
161	2014.5901	35.7826	1.5296
36	2014.3611	16.2778	0.7181
111	2015.2818	17.8198	0.7749
49	2014.8776	30.1633	0.9029
39	2015.7179	27.8462	1.1206
29	2015.7586	32.8621	1.3218
185	2014.4108	25.0973	0.8979
39	2014.9487	29.9487	1.1627
84	2016.1786	21	1.066
49	2015.102	27.7143	0.9896
49	2015.2449	24.6531	0.9628
39	2014.7692	17.359	0.761
25	2015.04	13.44	0.5273
127	2014.9449	17.1417	0.7457
155	2015.3677	21.9032	0.9778

69	2015.2899	26.2754	1.1872
61	2013.65	25.6393	0.8046
26	2016.2308	22.7308	0.8893
83	2015.2683	21.0361	0.7617
29	2017.7857	15.8276	1.6879
56	2015.3036	18.6071	0.8349
2453	2015.44	19.81	0.8584
27	2014.1111	25.1481	0.9045
82	2014.8902	33.2683	1.5476
119	2016.161	11.2857	0.6179
52	2014.3077	29.4038	1.0549
38	2015.1316	27.7895	1.0987
145	2014.6276	24.731	0.9248
25	2015.44	14.88	0.6217
66	2015.4091	17.3333	0.7263
135	2015.0074	20.1556	0.7523
25	2014.68	51.24	1.6341
45	2014.8222	28.7111	0.9983
33	2015.8485	9.4242	0.7418
34	2018.3824	16.2647	2.0284
25	2015.28	21.32	0.9412
101	2015.29	26.802	1.103
109	2015.9541	22.1835	0.8868
26	2014.5833	29.7308	0.965
103	2015.8155	16.6796	0.9374
30	2016.3	21.0667	1.0841
78	2015.0513	16.0769	0.6242
25	2014.24	61.72	1.8857
87	2014.2874	21.3908	0.756
137	2013.7518	49.2482	1.407
283	2016.0919	15.9364	0.8426
35	2015.9143	13.2857	0.6291
121	2016.3223	25.3554	1.2286
27	2016.2692	89.3333	4.2715
675	2014.5952	30.7437	1.0614
132	2015.9848	16.9167	1.0379
305	2015.3333	20.3279	0.8435
28	2014.4643	12.6786	0.5289
26	2015.2692	16.4615	0.7518
38	2014.8947	13.6053	0.6509
46	2015.1591	14.8478	0.6772
38	2016.2162	17.6842	0.9071
47	2015.4043	25.234	0.9615
95	2016.4632	12.5684	0.7289
56	2015.8929	18.1964	0.9692
37	2014.7838	25.5135	0.8429
44	2015.2273	21.5909	1.0517

38	2016.1842	12.8684	0.6589
195	2015.6719	16.2154	0.8782
36	2014.3056	21.6389	1.0314
32	2013.9688	37.375	1.1725
36	2014.8056	40.1667	1.2792
72	2015.8333	30.25	1.1261
76	2016.8289	22.0789	1.4686
38	2017.0526	21.3684	1.4373
31	2014.7742	23.5484	1.0423
901	2015.6011	18.2386	0.8727
208	2014.7596	33.4471	1.2408
123	2014.9675	37.4878	1.3273
87	2014.7356	43.9425	1.6026
69	2014.1594	41.5652	1.4163
30	2014.7333	25.1333	0.8137
46	2014.5217	24.8478	0.8055
61	2017.1	18.7213	1.2805
92	2015.8696	16.663	0.7735
28	2015.6071	14.75	0.6112
80	2015.2625	22.9	0.9299
28	2014.4286	22.1071	0.6973
27	2016.3333	24	1.5239
75	2015.8	26	1.1832
29	2014.5517	21.3793	0.7582
53	2015.8679	21.5849	0.9331
376	2015.8777	24.1356	1.0831
25	2016.32	21.36	1.1546
75	2016.36	13.9467	0.9176
31	2016.0323	17.5161	0.8796
37	2016.9189	13.4595	1.0616
29	2014.4828	46.6897	1.7361
26	2015.2308	29.4615	1.1973
27	2014.8889	23.6296	0.9641
46	2015.6522	30.8696	1.0226
41	2015.7805	20.0976	0.9789
188	2014.2234	47.383	1.5901
31	2015.2903	31.7097	1.4223
27	2013.8148	46.6667	1.7956
259	2014.7259	34.0154	1.2762
25	2014.56	19.2	0.83
30	2016.5517	7.3	0.6088
198	2016	24.6465	1.2732
29	2015	45.7931	2.0185
296	2014.9899	25.0304	0.9204
58	2014.5862	35.3448	1.2136
31	2015.6129	14.0645	0.8052
34	2015.8235	20.4118	0.8445

137	2015.7353	17.1533	0.8175
41	2016.122	25.5854	1.1007
56	2015.8393	20.9643	1.0522
46	2015.4667	31.2609	1.3527
31	2015.0968	26.8387	1.0881
28	2016.0714	18.9286	1.1249
76	2015.0533	38.6842	1.2449
65	2015.3231	23.7231	1.0912
62	2015.3226	38.129	1.36
158	2015.519	26.9557	1.2334
43	2014.3023	12.6047	0.4688
44	2015.3256	16.7045	0.7255
72	2015.0139	17.9306	0.7607
220	2016.5091	17.5545	1.2148
119	2016.2966	18.7815	0.9176
28	2015.8571	23.9643	1.4366
1210	2015.602	19.757	0.9802
606	2014.8826	34.2426	1.3397
47	2015.9783	18.0213	1.089
64	2015.5625	21.25	1.1329
42	2016.3659	21.2619	1.2029
28	2017	19.75	1.3207
378	2014.9101	21.5344	0.8694
27	2016.037	42.2593	2.0074
36	2015.7778	20.3333	0.9287
26	2015.0385	26.3462	1.067
46	2015.8696	18.1739	1.0138
76	2015.9079	24.4737	1.3321
468	2015.1303	21.0363	0.858
133	2015.3459	16.2256	0.677
25	2015.96	10.36	0.5699
27	2015.2963	31.963	1.452
53	2014.9623	27.8491	1.1112
25	2015.96	32.44	1.4303
4675	2015.4668	22.5405	0.9653
45	2014.7111	16.6222	0.6215
43	2015.4884	20.6279	1.0713
62	2015.7903	36.8226	1.5215
26	2015.5769	20.2692	0.8336
105	2014.7429	35.1524	1.2348
90	2013.7	24.0778	0.757
899	2015.5869	19.9066	0.935
295	2015.6475	17.2475	0.8476
27	2015.8889	17.6296	1.0318
143	2015.8857	13.7972	0.7351
33	2015.2424	42.3636	1.4413
65	2015.4769	30.6615	1.1036

62	2015.3226	22.0645	1.0086
42	2015.8095	17.3095	0.7779
184	2015.3587	36.6576	1.4533
28	2014.5	31.3214	1.0274
48	2015.8125	30.3542	1.0025
30	2014.9333	25.0667	1.2288
26	2015.1538	13.1923	0.4948
27	2015.7407	23.3704	0.945
235	2014.7564	26.4894	0.9622
44	2015.1591	14.6591	0.6857
51	2014.098	12.902	0.4167
31	2015.4194	19.5806	0.8634
30	2014.9667	25.3	0.9519
42	2015.8537	12.1667	0.6605
300	2015.7933	20.9267	0.9229
29	2015.5862	22.8621	0.9088
149	2015.1879	14.6711	0.6457
27	2015.4444	23.2222	1.0213
88	2015.1023	22.7273	1.1286
29	2017.4828	21.5862	1.4827
52	2015.1731	13.5577	0.6434
65	2015.9077	16.1077	0.9552
66	2014.9848	16.6515	0.8557
46	2014.8043	46.3261	1.6847
59	2014.75	20.9492	0.8616
26	2015.1154	18.1154	0.6804
43	2015.2558	14.0698	0.4948
161	2015.2919	21.4534	0.8747
50	2014.66	36.42	1.2944
31	2015.3548	16	0.7029
35	2015.4118	14.2571	0.7005
164	2015.1646	19.6829	0.8859
79	2015.7308	15.8734	0.8386
26	2014.6538	9.1538	0.3322
34	2014.4706	23.3235	0.7987
106	2015.5472	14.4811	0.7202
60	2013.8667	20.8333	0.733
30	2014.1333	32.5	1.0296
54	2015.537	13.3704	0.729
105	2015.019	16.8857	0.6794
162	2014.2236	21.8704	0.7917
51	2015.098	14.1176	0.533
71	2016.8429	15.1972	1.0413
90	2015.9111	16.6667	0.7256
25	2015.8	34.6	1.3669
31	2015.0968	19.8065	0.828
28	2014.5714	21.5714	0.7006

37	2014.5405	29.973	1.2853
80	2014.5875	24.7	0.9044
32	2014.8125	46.25	1.6317
481	2015.6715	19.4449	0.9733
43	2014.3023	33.4651	1.0823
54	2014.7593	23.9259	0.9703
34	2015.8824	33.7353	1.2118
77	2016.2763	26.4935	0.9904
105	2015.219	24.1524	1.0096
71	2014.9155	10.3944	0.5284
74	2015.7973	24.8649	1.2
28	2016.5357	26.4643	1.4436
317	2015.3659	24.4511	1.0585
40	2015.35	11.8	0.6031
31	2014.9355	28.4516	0.9904
36	2014.8056	15.25	0.614
26	2015.7692	32.8462	1.5735
31	2015.1613	23.3548	0.9482
25	2014.84	25.88	0.9392
29	2014.0345	31.6897	0.9899
28	2015.8214	9	0.4948
28	2013.7143	28.6786	0.8747
30	2014.3	37.8333	1.2983
204	2015.5813	14.7451	0.6665
92	2015.4505	16.9022	0.7367
69	2015.7536	12.6667	0.5934
135	2015.5896	23.9185	1.0109
42	2014.3095	23.2857	0.8176
101	2014.89	21.4059	0.8201
163	2015.4417	16.3129	0.6712
46	2015.7333	12.6087	0.7347
25	2016.2	17.44	0.909
1071	2015.8939	17.9412	0.8778
58	2015.1552	18.6034	0.7941
32	2016.7812	32.5312	2.094
159	2015.4403	20.1447	0.9596
77	2016	19.4935	1.1211
33	2015.4545	18.697	1.0117
31	2015.5161	19.7742	0.9
26	2016.6923	20.2308	1.3616
64	2015.7937	84.1094	3.0709
82	2015.9878	26.5488	1.2198
47	2016.9574	22.2979	1.7398
109	2014.3486	21.6055	0.767
30	2015.0667	30.2	1.1723
69	2016.7536	19.5072	1.1399
176	2016.0568	16.2727	0.8037

157	2014.9679	28.8662	1.138
357	2015.9804	19.4286	0.9287
36	2015.1143	15.1389	0.6463
835	2015.6987	21.9928	1.0193
64	2014.1875	66.9062	2.2835
34	2014.9706	15.8529	0.6875
27	2016	24.9259	1.3342
79	2016.3038	22.8354	1.1644
741	2015.248	16.3698	0.722
55	2015.9455	15.8545	0.831
25	2014.92	26.84	0.9701
382	2015.0105	19.4005	0.8446
70	2017.3	21.0143	1.7449
25	2014.4	34.2	0.996
37	2017.3243	38.5946	2.2679
205	2015.8585	25.8878	1.4106
29	2016.3793	36.6552	1.4413
135	2015.7761	28.5333	1.2133
25	2014.4	39.48	2.3527
26	2014	24.7308	0.8831
263	2015.7871	18.4449	0.8362
26	2016.0385	20.4615	1.2284
462	2015.3492	20.697	0.8852
105	2016.5143	18.2762	1.0002
44	2014.9091	17.8182	0.7013
28	2015.6071	13.8571	0.6281
29	2014.8966	35.4138	1.2913
28	2014.5714	12.7143	0.44
27	2015.4074	16.1481	0.6982
87	2014.2184	41.1839	1.4911
212	2015.3443	14.283	0.6488
31	2015.5667	17.7742	0.6841
78	2015.7662	47.4359	2.2503
109	2015.8796	13.3211	0.7374
27	2017.4074	12.4815	0.8695
160	2016.1813	18.8125	1.0215
77	2014.7792	25.1948	0.9891
51	2014.549	38.5686	1.33
52	2016.1538	22.9615	1.182
193	2015.3438	28.7202	1.1056
220	2015.2811	33.5727	1.2964
29	2015.0345	31.9655	1.5962
40	2014.975	21.025	0.8741
55	2016.0182	15.8545	0.8555
81	2015.7654	12.8519	0.6625
83	2015.9759	13.6627	0.6516
184	2016.0217	30.9728	1.5598

32	2015.1562	15.7188	0.6776
54	2014.2037	40.1852	1.4537
47	2015.6809	16.4468	0.9806
52	2014.4706	20.4231	0.8828
29	2015.931	19.069	0.8818
122	2015.5492	22.8607	0.9961
51	2014.9608	15.6471	0.5866
73	2014.9452	90.3425	3.2061
29	2014.931	46.0345	1.7169
78	2015.1818	31.7949	1.3519
34	2015	24.7941	0.8939
66	2014.5758	27.0606	1.0583
51	2017.1765	19.9804	1.826
32	2017.5938	19.4062	1.8047
79	2017.0633	18.8101	1.4532
453	2017.0774	17.1678	1.427
26	2016.1538	22.3462	1.5728
36	2015.8333	19.5556	0.8478
39	2015.6923	42.2051	2.211
68	2016.3088	12.3235	0.8514
64	2015.4375	19.625	0.892
36	2017.1944	15.2222	1.1008
75	2015.0267	21.9733	0.7812
26	2015.5385	22.7692	1.154
265	2014.4981	36.1019	1.3569
44	2016.9535	21.0909	1.5824
96	2016.2917	15.5312	0.815
32	2014.75	23.3438	0.7879
66	2016.2424	16.4091	1.1876
28	2014.1786	18.8571	0.7476
32	2017.129	10.4688	0.7155
27	2015.4815	13.0741	0.5416
49	2015.5714	23.898	1.0975
39	2016.0256	12.5641	0.7995
113	2016.3214	20.0619	0.8853
29	2015.7241	27.7241	1.1526
87	2016.4138	18.2299	0.9685
67	2016.6716	22.791	1.2696
70	2015.6286	34.9	1.4706
101	2015.4	22.1782	1.0166
30	2014.5333	99.4333	2.2232
1182	2015.1903	31.8959	1.2856
253	2015.1633	24.5138	0.9403
136	2014.8529	30.75	1.2431
42	2018.1667	10.1905	1.5552
31	2015.7097	28.871	1.3902
39	2016.2308	27.2051	1.3973

39	2017.0769	16.2564	1.5704
26	2013.6923	12.9615	0.3986
78	2016.4744	16.7179	0.847
28	2016.5714	17.1071	0.9711
124	2014.9435	42.4274	1.4204
137	2016.2482	18.6277	1.0473
34	2014.7059	30.6471	0.9424
44	2016.0227	14.9545	0.9219
30	2015	15.5	0.8294
43	2015.5349	16.2791	0.7873
347	2015.8439	17.8415	0.8489
45	2013.5455	21.1333	0.6433
26	2015.8462	28.4231	1.301
46	2015.1087	20.0652	0.7738
73	2017.3014	12.5479	1.1223
50	2015.4082	12.82	0.6174
25	2015.16	18.8	0.8369
29	2015.6897	8.8621	0.5187
37	2015	18.3514	0.6935
61	2016.0328	14.3934	0.7783
142	2016.8085	12.4437	0.756
296	2015.4983	22.5709	1.0076
64	2016.8438	17.4844	1.0951
107	2015.4245	15.0467	0.7168
26	2016.5769	21.5	1.5691
99	2015.9192	16.3434	0.8498
26	2016.5385	15.1538	1.0773
44	2015.6744	21.4545	0.932
33	2014.2727	46.7576	1.3991
1946	2015.4416	22.4928	0.9842
69	2015.5942	23.8116	1.1977
34	2016.3824	26.2353	1.7983
114	2015.2105	22.5965	1.0115
25	2015.88	20.76	0.8891
70	2015.5286	15.9857	0.9024
273	2014.952	19.5568	0.8084
174	2016.3161	15.7644	0.8276
95	2015.6421	14.5895	0.6323
29	2017.2069	11.2759	0.9454
310	2016.0645	24.8968	1.2932
188	2015.4439	20.4521	0.8968
733	2015.6762	16.3015	0.79
43	2015.2326	14.4884	0.6324
42	2016.5	19.8095	1.1595
25	2018.12	8.08	1.3251
33	2015.9697	21.0606	1.3726
29	2015.6207	24.7931	1.0504

58	2015.0862	21.4828	0.8536
41	2015.1707	17.5366	0.693
41	2015.2683	18.4146	0.7284
33	2015	16.0606	0.6767
35	2016.2857	16.1429	1.5769
94	2015.1809	16.5532	0.704
28	2016.0714	18.5714	1.0552
63	2015.4603	17.619	0.7389
173	2015.1618	24.1214	1.0177
64	2016.0469	24.4688	1.0737
44	2014.9545	68.7955	2.5834
69	2015.2647	17.9855	0.9616
206	2015.165	17.8155	0.7617
366	2016.8607	20.6421	1.6218
105	2016.6381	22.8286	1.5278
99	2017.596	20.3939	1.9598
40	2014.45	29.2	0.8604
141	2015.078	27.773	1.0969
32	2015.6562	19.4688	1.0307
30	2015.8667	19.8	0.9764
28	2016.7857	12.0357	0.8238
25	2017.04	13.48	0.9301
49	2015.0612	21.8163	0.8368
67	2014.9701	36.1045	1.411
65	2015.3385	11.3692	0.4665
45	2017.0889	17.1111	0.9549
29	2015.4483	22.6552	1.0262
33	2018	23.4848	2.5581
34	2014.3529	21.4118	0.6346
25	2016.92	14.08	1.0978
32	2015.1562	15.9062	0.6657
27	2017.1852	19.1111	2.2983
34	2017.3529	16.9706	1.6577
142	2014.7958	12.0352	0.4483
51	2015.4902	18.3529	0.9915
26	2016.2692	17.4615	0.8828
60	2015.3276	23.4333	0.9232
126	2015.808	30.3889	1.3474
75	2015	17.36	0.6967
67	2014.5522	22.5522	0.7898
138	2014.913	28.5217	1.0123
27	2016.0385	11.5185	0.733
70	2016.058	23.7	1.2889
29	2015.3103	28.069	1.2554
47	2016.4894	16.8723	0.9121
34	2015.2353	26.3235	0.8491
27	2017.1852	18	1.0507

27	2016.037	49	1.8831
32	2014.2812	20.75	0.653
38	2015.5789	29.3158	1.1616
62	2017.0323	16.4355	1.0765
93	2015.2473	16.7097	0.686
228	2015.2193	16.8904	0.7255
28	2017.0769	17.1071	1.6354
40	2017.75	17.375	2.147
43	2014.3953	99.0465	2.7963
491	2016.0103	17.6171	0.8385
54	2016.1852	21.2407	1.0307
34	2015.2059	39.1176	1.3846
40	2014.925	47.4	1.2864
64	2016.381	15.2969	0.7539
54	2015.1852	23.7222	1.0912
46	2015.3043	17.1522	0.7311
86	2015.6628	21.3605	1.0556
513	2015.5809	23.4172	1.0083
34	2014.8529	25.2647	0.942
629	2015.8328	16.9603	0.7954
73	2016.3973	15.9863	0.8806
40	2015.4	47.35	1.2869
45	2015.1778	23.6	1.3239
555	2014.8574	32.3658	1.2111
62	2014.6613	27.9839	1.016
27	2015.4074	25.3704	1.4363
118	2016.2522	14.9407	0.7993
62	2014.9839	34.5968	1.2222
45	2013.9778	77.2667	1.9265
504	2015.1567	18.2996	0.7601
42	2014.9286	19.619	0.7125
67	2015.5224	16.7313	0.7275
30	2015.3	20.0667	0.7079
29	2016.4828	12.3793	0.6441
35	2014.6286	29.5143	1.0732
218	2015.2857	27.1376	1.0947
25	2016.12	13.64	0.8141
39	2016.3846	25.8462	1.6537
55	2015.5636	15.9455	0.8535
133	2015.5909	17.0376	0.836
357	2015.8034	16.112	0.8575
39	2014.4103	26.3333	0.8699
31	2015.7742	24.0645	1.1945
25	2014.88	15.2	0.5601
108	2015.1204	40.8796	1.6647
76	2014.56	19.6184	0.8044
43	2015.6512	16.5116	0.8114

41	2017.0976	11.8049	0.8008
267	2015.1504	11.3633	0.5691
183	2014.9508	35.8579	1.2671
1019	2015.5419	18.368	0.9137
422	2015.2625	19.4123	0.8301
29	2015.2759	20.7586	0.9813
64	2015.9531	21.7812	1.0813
52	2015.1923	42.3462	1.7632
26	2015.76	11.1154	0.6087
134	2015.8045	21.0224	0.9795
36	2015.6944	26.2778	1.1847
59	2014.9123	24.4237	1.2485
39	2015.641	30.5385	2.5558
27	2014.037	23.963	0.8577
29	2015.5	18.2414	0.7591
30	2016.2	14	0.7496
219	2014.9863	38.2785	1.539
145	2016.4444	18.0759	1.3423
47	2014.8085	18.3404	0.7028
36	2015.0556	32.6667	1.5012
70	2014.8116	18.0571	0.5822
97	2015.3438	26.3093	1.0062
536	2016.0879	20.2537	1.0246
26	2015.3846	22.9231	1.2728
109	2015.9541	21.8899	0.9933
112	2015.5495	22.625	0.9626
26	2013.8077	30	0.8938
407	2015.3243	14.226	0.7079
425	2014.9012	25.2188	1.0543
563	2015.5819	16.6128	0.8451
26	2015.0385	51.3077	2.2498
171	2015.2824	23.3099	1.013
76	2014.9079	14.6316	0.63
75	2015.6933	25.6933	1.1693
770	2015.8592	17.3922	0.8875
68	2016.5224	12.8382	0.9132
99	2015.3438	12.3232	0.6142
34	2016.5882	26.2059	1.4427
47	2016.383	19.4043	0.9661
31	2015.2903	17.8065	0.8482
59	2015.7966	17.7119	0.9351
73	2015.6438	29.9178	1.3325
78	2015.4211	25.6923	0.9953
73	2015.6667	21.3836	1.161
28	2013.8519	15.5714	0.4943
79	2015.2692	16.6076	0.6892
31	2016.4194	13.3548	0.8475

45	2015.1556	16.8	0.693
27	2015.4074	25.9259	1.0681
85	2014.6071	24.6588	0.9739
415	2015.9565	21.7831	1.1097
27	2016.963	20.3704	2.0946
44	2017.0455	18.1136	1.3688
51	2015.9412	22.6078	1.2202
26	2015.8	15.2308	0.7932
29	2016.4828	15.9655	0.8859
65	2015.5077	22.2923	1.0659
73	2014.9722	19.5753	0.7509
67	2014.4478	39.3433	1.5762
64	2016.0469	19.8906	1.1798
40	2017.1538	13.225	1.1241
32	2016.5	16.1562	0.8883
26	2015.5	15.5769	0.8314
33	2014.0303	25.9394	0.9618
153	2015.4052	28.9673	1.2332
27	2016.1538	17.1481	0.9429
42	2015.7381	25.4286	1.0086
25	2015.52	14.88	0.7721
137	2015.6642	24.1971	1.0858
26	2015.3077	32.9615	1.6069
115	2017.2957	15.9739	1.1003
26	2014.8846	31.3846	0.9765
49	2015.0612	19.0816	0.8139
290	2016.0315	22.869	1.1753
64	2016.5781	20.0938	1.3416
306	2015.6459	20.4706	0.8902
31	2014.8387	33.2258	1.2703
52	2015.5192	23.5577	1.1559
27	2014.6667	47.1481	1.6105
36	2016.5556	20.8333	1.0921
26	2015.7692	12.0385	0.7295
77	2014.5789	17.7922	0.812
117	2014.1709	22.7692	0.7837
53	2015.8077	14.7547	0.6952
140	2015.9353	20.25	0.9868
162	2015.679	54.284	2.0603
150	2015.4362	17.8067	0.7949
998	2015.8763	20.3607	0.9456
57	2016.1404	17.193	0.7051
92	2015.1196	15.1196	0.6771
52	2015.9231	17.5	0.7611
27	2014.2963	30.3704	0.9875
42	2015.1905	19.3333	0.797
133	2015.7895	17.1729	0.9349

123	2015.6992	28.1626	1.1078
53	2015.1731	26.1698	0.9205
26	2015.1538	13.1538	0.4955
76	2014.36	27.8947	0.96
34	2016.7353	14.2941	1.3995
39	2015.359	16.0513	0.9234
284	2015.257	42.0775	1.6948
91	2015.1667	18.2857	0.7409
35	2015.9714	55.4857	2.2435
54	2015.9259	19.2778	1.2012
176	2015.7886	18.6932	0.8355
34	2016.3235	19.8235	1.0581
45	2014.0444	27.7778	0.9993
174	2015.2414	29.7989	1.3157
41	2015.2683	25.6098	1.0268
92	2016.0217	18.9674	1.1673
43	2015.6047	26.6744	0.9597
1054	2015.4534	18.8805	0.8616
33	2014.9091	14.5455	0.6028
50	2015.82	19.54	1.1481
32	2015.5312	8.5	0.6269
25	2015.32	20.12	0.7948
134	2015.4662	18.5597	0.836
43	2015.0465	27.1163	0.9732
301	2015.0569	22.186	0.8304
54	2017.1481	13.5	1.0289
29	2017.0714	17.069	1.2802
46	2015.913	15.1522	0.7886
32	2014.9062	33.625	1.1792
77	2015.0263	28.7273	1.2315
307	2016.1148	16.7622	0.8709
33	2016.3333	15.7273	0.8672
26	2015.0385	46.6923	2.1551
119	2015.2627	25.0756	1.0122
29	2015.4138	37.0345	1.4338
162	2014.8395	41.1728	1.3923
25	2014.2	32.12	1.1976
35	2014.8571	47.5714	1.4465
40	2013.4	39.5	1.067
35	2016.5429	31.6	1.5339
50	2015	16.22	0.7158
28	2016.25	12.8571	0.8637
42	2016.619	8.6905	0.6447
40	2015.975	21.5	0.9657
88	2014.9659	28.4432	1.2299
157	2015.2293	24.3185	1.2763
37	2017.25	11.1892	1.0741

26	2015.3077	7.5769	0.3797
58	2014.3793	29.7759	1.0579
27	2016.963	21.4444	1.8132
239	2016.2941	23.1213	1.3324
31	2014.6452	21.2903	0.7079
120	2014.9667	29.6	1.1327
27	2012.4815	58.8889	1.406
195	2015.4308	38.8769	1.6676
208	2014.7788	26.5	1.0213
298	2015.0604	27.8121	1.1603
75	2016.32	22.2667	1.2349
33	2015.4688	20.9091	0.9139
263	2014.4183	41.3536	1.3273
89	2015.4944	31.1124	1.2388
29	2014.1379	91	2.7122
155	2015.0774	35.7806	1.2958
461	2015.6486	24.5445	1.1687
35	2014.8286	21.5143	0.9694
37	2015.1622	21.7297	0.9319
26	2015.3846	10.1923	0.5047
69	2014.6912	24.5942	0.9515
54	2016.463	14.2593	0.9114
64	2015.25	28.7031	1.2
41	2015.1707	22.7317	0.9218
30	2017.8333	17.5	1.8327
319	2015.261	20.0846	0.937
40	2014.625	17.925	0.5902
28	2016.2143	12.2143	0.5841
68	2015.0294	12.1324	0.4957
66	2015.2121	31.0606	1.161
26	2014.6538	14.3846	0.4944
55	2013.9455	20.7273	0.556
82	2014.8049	23.5122	0.8452
28	2015.5357	21.75	1.1856
30	2015.5333	15.6333	0.6848
32	2016.5625	15.3438	0.9267
30	2015.2	18.5667	0.7372
44	2015	19.4773	0.8797
67	2015.8485	14.9851	0.8621
44	2014.1136	23.9091	0.8605
83	2015.0482	33.2048	1.2629
229	2015.559	17.5633	0.7675
25	2014.96	21.72	0.7542
29	2015.069	42.5862	1.4311
