# Supplementary material for: A Bibliometric Analysis of Cyclophosphamide, Methotrexate, and Fluorouracil Breast Cancer Treatments: Implication for the Role of Inflammation in Cognitive Dysfunction
Source: Front Mol Biosci. 2021 Aug 20;8:683389. doi: 10.3389/fmolb.2021.683389 (PMC8417522; doi:10.3389/fmolb.2021.683389)
Supplement: Supplementary file 3 [file DataSheet6.PDF]

id	label	x	y	cluster	Links
134	activation	0.5877	-0.2037	1	95
212	adipose-tissue	-0.3985	-0.5688	1	55
388	angiogenesis	-0.1665	-0.9504	1	78
672	autophagy	0.8162	-0.5177	1	46
984	breast-cancer	-0.2284	-0.1701	1	114
985	breast-cancer cells	0.6291	-0.7092	1	44
1298	cells	0.089	-0.3114	1	104
2013	differentiation	0.4898	-0.0334	1	53
2119	down-regulation	0.3513	-0.919	1	45
2257	emt	0.1128	-1.0636	1	62
2480	expression	0.2399	-0.2442	1	114
2789	gene-expression	0.3141	-0.3759	1	71
2793	genes	-0.064	-0.798	1	59
2950	growth	0.3945	-0.7341	1	80
3881	invasion	0.2161	-0.915	1	56
4346	macrophages	-0.0708	-0.4492	1	81
4561	metastasis	-0.0107	-0.936	1	87
4629	microenvironment	0.263	-0.6259	1	53
4651	micrornas	0.1029	-0.8219	1	51
5008	necrosis-factor-alpha	-0.0807	-0.0369	1	47
5103	neutrophils	-0.2259	-0.4518	1	44
5119	nf-kappa-b	0.7069	-0.4139	1	91
5380	ovarian-cancer	-0.5604	-0.5674	1	47
5497	pathways	0.5721	-0.4308	1	74
5997	progression	-0.0809	-0.3052	1	80
6004	proliferation	0.5393	-0.8359	1	69
6076	proteins	0.138	-0.4744	1	69
6271	receptors	0.2359	0.1223	1	82
6384	resistance	0.2461	-0.7691	1	66
6646	signaling pathways	0.4745	-0.5524	1	55
6821	stat3	0.0625	-0.6633	1	50
6839	stem-cells	0.4313	-0.3806	1	51
7023	t-cells	-0.0012	-0.1653	1	66
7203	tnf alpha	0.2639	-0.0613	1	74
7382	tumor microenvironment	-0.4912	-0.7203	1	74
7403	tumor-associated macrophages	-0.2338	-0.8259	1	60
7472	up-regulation	0.7976	-0.6483	1	50
618	association	-0.7228	0.2702	2	82
843	biomarkers	-0.4287	0.154	2	69
898	body-mass index	-0.8087	-0.3714	2	57
1023	c-reactive protein	-0.6929	0.4277	2	80
1844	cytokines	0.0468	0.5634	2	87
1937	depression	-0.3282	0.9936	2	47
1968	diagnosis	-0.4047	0.3381	2	56
2042	disease	-0.053	0.4787	2	73
2115	double-blind	-0.2031	0.5765	2	45

2552	fatigue	-0.4909	0.9061	2	44
3026	health	-0.1331	0.7882	2	48
3493	immunity	-0.1093	0.1968	2	63
3538	impact	-0.5502	0.2715	2	66
3583	index	-0.95	0.5319	2	41
3660	inflammation	-0.0269	0.0739	2	117
3724	inhibitors	0.083	0.3189	2	67
3755	insulin-resistance	-0.5325	0.0295	2	50
3800	interleukin-6	-0.31	0.2618	2	61
4402	management	-0.2944	0.7006	2	50
4536	meta-analysis	-1.0182	0.3138	2	46
4537	metaanalysis	-0.7083	0.6966	2	55
4805	mortality	-0.7388	0.5778	2	54
5245	obesity	-0.647	-0.3631	2	83
5910	prevalence	-0.2658	0.8673	2	51
5912	prevention	-0.4275	0.749	2	53
6157	quality-of-life	-0.5698	0.6595	2	49
6445	risk	-0.4423	0.4867	2	92
6453	risk-factors	-0.6294	0.1163	2	37
6869	stress	-0.0384	0.8875	2	50
7640	women	-0.5846	0.477	2	69
52	5-fluorouracil	0.731	0.3676	3	54
106	acid	1.0088	0.2916	3	47
520	antioxidants	0.973	0.5572	3	43
559	apoptosis	0.9024	-0.1901	3	96
1489	cisplatin	0.4007	0.1473	3	58
1605	combination	0.5557	0.2341	3	50
1805	cyclophosphamide	0.5781	0.5667	3	64
1869	damage	1.1304	0.374	3	43
2121	doxorubicin	1.0341	-0.0143	3	49
2151	drug-delivery	0.6286	0.12	3	38
2211	efficacy	0.1761	0.6061	3	58
2980	gut microbiota	0.2727	0.549	3	42
3560	in-vitro	0.7508	-0.271	3	73
3565	in-vivo	0.3825	-0.15	3	54
3720	inhibition	0.6982	-0.0507	3	82
3734	injury	1.1201	0.553	3	35
4466	mechanisms	0.2865	0.2921	3	103
4546	metabolism	0.4423	0.358	3	60
4586	methotrexate	0.4154	0.6894	3	79
4604	mice	0.805	0.5272	3	54
4741	model	0.5983	0.4338	3	62
4834	mucositis	0.7043	0.7835	3	39
4958	nanoparticles	0.7761	0.1785	3	41
5133	nitric-oxide	1.1181	0.2036	3	38
5409	oxidative stress	0.9083	0.136	3	90
6232	rats	0.9619	0.7209	3	38

6428	rheumatoid arthritis	0.1391	0.7186	3	69
6431	rheumatoid-arthritis	0.3249	0.8052	3	45
7251	toxicity	0.693	0.6614	3	55
220	adjuvant chemotherapy	-1.0772	0.1413	4	47
842	biomarker	-0.402	-0.2262	4	57
1062	cancer	0.0992	-0.0449	4	116
1146	carcinoma	-0.3442	-0.3621	4	66
1385	chemotherapy	0.0518	0.1806	4	113
1603	colorectal-cancer	-0.4099	-0.0767	4	85
2742	gastric-cancer	-0.5155	-0.3916	4	66
3536	immunotherapy	-0.657	-0.2294	4	65
4277	lung-cancer	-0.7386	-0.5246	4	60
5021	neoadjuvant chemotherapy	-0.8348	-0.179	4	53
5077	neutrophil	-0.9559	-0.0328	4	43
5097	neutrophil-to-lymphocyte ratio	-1.1074	-0.1387	4	40
5367	outcomes	-0.878	0.338	4	58
5458	pancreatic-cancer	-0.3976	-0.8129	4	58
5960	prognosis	-0.9397	-0.3166	4	81
6041	prostate-cancer	-0.129	-0.6319	4	61
6183	radiation-therapy	-0.5299	-0.1472	4	59
6378	resection	-1.1157	-0.01	4	44
6935	surgery	-0.9258	0.1372	4	42
6941	survival	-0.7709	-0.0281	4	97
7132	therapy	-0.1504	0.3694	4	101
7429	tumors	-0.2698	-0.663	4	54

Total link strength	Occurrences	sAvg. pub. year	Avg. citations
460	97	2020.3053	2.268
136	27	2020.08	4.2963
306	57	2020.3455	3.386
114	22	2020.2273	4.5
1784	444	2020.3468	2.714
89	22	2020.2273	3.9545
665	139	2020.3066	2.4388
102	25	2020.36	3.2
102	22	2020.3182	4.5909
217	48	2020.25	6.5417
1101	234	2020.3231	2.9274
221	49	2020.3125	3.6735
131	29	2020.1724	2
354	71	2020.3768	2.2817
164	27	2020.2593	2.5185
242	58	2020.3333	3.3103
458	87	2020.2381	4.0805
130	25	2020.1667	3.4
112	22	2020.4091	2.2727
91	20	2020.5	1.05
93	22	2020.2381	3.0455
402	88	2020.3182	5.0455
93	22	2020.3636	2.4091
249	52	2020.1569	3.6538
235	45	2020.1333	3.1556
225	45	2020.2558	2.9333
167	36	2020.3143	2.2222
206	44	2020.2093	2.4545
198	40	2020.2564	2.55
130	30	2020.4667	2.8333
110	25	2020.5417	1.68
113	25	2020.4	5.16
161	41	2020.35	3.6585
175	34	2020.2941	2.8235
244	48	2020.2826	3.0417
166	34	2020.3636	1.8529
127	24	2020.2727	3.0833
295	60	2020.1667	1.9333
171	33	2020.2188	2.5758
150	29	2020.25	5.6207
262	60	2020.3571	1.7833
310	69	2020.4127	1.6522
136	24	2020.55	1.7083
112	27	2020.36	1.6296
195	53	2020.2308	2.2264
82	28	2020.1786	3.7857

143	25	2020.5	1.68
115	27	2020.24	2.3333
132	30	2020.2759	2.4667
146	35	2020.3235	2.3429
98	20	2020.6111	1.05
3144	753	2020.3305	2.3971
153	34	2020.303	2.3235
91	20	2020.3158	3.65
156	30	2020.5185	1.4333
104	32	2020.3571	2.1875
106	23	2020.1739	2.087
133	28	2020.16	2.3571
141	31	2020.1667	2.3226
396	87	2020.3537	2.7011
107	23	2020.3684	1.0435
113	23	2020.25	1.6087
122	27	2020.1538	2.3704
382	92	2020.3146	2.1087
70	21	2020.25	2.2381
113	26	2020.4583	1.9615
206	43	2020.381	2.1395
120	32	2020.2903	1.6875
108	21	2020.3684	3
112	26	2020.4348	2.0385
631	124	2020.3475	2.5
154	35	2020.2941	2.8
102	22	2020.3333	2
199	58	2020.2692	1.6207
114	22	2020.5	1.7273
137	35	2020.3871	2.8857
67	20	2020.4211	3.7
132	33	2020.303	1.6364
95	29	2020.4815	2.069
222	58	2020.3393	2.9483
105	24	2020.4545	2.2917
242	50	2020.2553	2.2
110	23	2020.4737	1.7826
437	93	2020.2273	3
142	31	2020.4828	2.1935
412	110	2020.3069	1.8727
131	34	2020.303	2.2647
132	32	2020.4	1.5
88	23	2020.3478	1.6522
110	27	2020.3333	2.2963
90	22	2020.45	1.7727
574	134	2020.3607	2.1866
89	20	2020.3889	1.55

200	53	2020.36	0.9057
134	37	2020.3143	3.3784
137	31	2020.1786	2.6129
121	26	2020.1923	1.9231
137	31	2020.2333	2.5484
728	180	2020.2727	3.3667
198	44	2020.1628	2.1818
1037	254	2020.3251	2.4803
288	66	2020.2923	3.6061
186	35	2020.4286	1.5143
219	62	2020.3833	4.0806
174	40	2020.2821	3.1
135	34	2020.3333	3.4118
86	21	2020.35	2.4762
94	22	2020.1905	2.5909
153	32	2020.2258	3.25
147	35	2020.3143	3.0571
451	103	2020.2222	2.6019
161	33	2020.2667	2.9394
131	31	2020.3	2.0968
107	21	2020.15	1.8571
98	23	2020.2273	1.6522
672	159	2020.268	2.4969
393	103	2020.3131	1.835
145	35	2020.3714	2.0857

Avg. norm. citations
1.4212
1.9413
2.1773
2.4015
1.5941
2.0501
1.4151
1.5809
2.7915
3.1325
1.6098
2.0511
1.0368
1.2341
1.1775
2.088
2.0787
1.7697
2.2634
1.4806
1.8935
2.7821
1.6201
1.7102
1.3559
1.5463
1.1335
1.3374
1.2887
2.0753
1.3395
2.6289
2.0114
1.4403
1.5214
1.326
3.1479
0.8736
1.2497
2.8588
1.3024
1.2569
0.8413
1.2403
1.0538
1.8105

1.3395
1.3679
1.2028
1.0556
0.8157
1.4851
1.4525
2.4261
0.9875
1.2213
1.0086
0.9668
1.164
1.8755
0.6722
1.1388
1.1138
1.1299
1.9012
1.0408
1.2186
1.1272
1.5953
1.4698
1.7453
1.4237
0.9763
0.9626
1.5999
2.0488
1.9759
0.8591
1.3918
2.3613
2.1643
1.3056
1.1388
1.6672
1.3438
1.0971
1.5787
0.8858
1.1575
1.9399
1.1127
1.4613
1.1163

0.6805
1.7298
1.2472
1.0243
1.4685
1.8016
0.9862
1.5842
2.1279
1.6066
2.6251
2.7475
1.9958
2.5346
1.1132
1.9594
1.5342
1.4928
1.6399
1.233
1.0431
1.232
1.3723
0.9134
1.1168
