# Supplementary material for: A Bibliometric Analysis of Cyclophosphamide, Methotrexate, and Fluorouracil Breast Cancer Treatments: Implication for the Role of Inflammation in Cognitive Dysfunction
Source: Front Mol Biosci. 2021 Aug 20;8:683389. doi: 10.3389/fmolb.2021.683389 (PMC8417522; doi:10.3389/fmolb.2021.683389)
Supplement: Supplementary file 4 [file DataSheet3.PDF]

id	label	x	y	cluster	Links
19	5-fluorouracil	-0.0274	-0.9808	6	14
33	accumulation	0.4798	0.6501	4	23
36	acid	-0.2912	-0.1462	1	17
47	activation	-0.3278	0.6665	5	35
70	adenosine	0.5506	0.9017	4	30
95	adriamycin	-1.1704	-0.2692	1	20
108	agents	-0.0995	-0.7276	6	13
110	aica-riboside	0.7297	0.9184	4	21
210	antigen	-0.802	0.4943	3	12
227	antioxidants	-0.5581	-0.0876	1	27
240	apoptosis	-0.9543	-0.0568	1	31
242	arachidonic-acid	0.5841	0.7668	4	15
249	arthritis	0.2971	0.9113	4	21
267	asthma	0.0934	0.6351	4	19
288	azathioprine	0.9601	-0.6743	2	20
311	bcl-2	-0.8018	0.0069	1	18
363	bone	0.3395	-0.3315	2	15
369	bone-marrow transplantation	-0.1036	-0.2966	3	13
392	breast	-0.9838	-0.1736	1	9
400	breast-cancer	-0.5613	-0.186	1	53
403	breast-cancer-cells	-1.0657	-0.2794	1	16
410	bronchoalveolar lavage	0.557	0.0823	2	21
440	cancer	-0.2902	-0.311	1	44
463	carcinogenesis	-0.9219	0.028	1	18
465	carcinoma	-0.3747	-0.6922	1	18
509	cells	-0.1446	0.0656	4	61
514	central-nervous-system	-0.6957	0.7515	3	14
537	chemotherapy	-0.0414	-0.3512	3	73
543	children	0.1143	-0.8821	6	8
577	cisplatin	-0.4812	-0.4854	1	16
578	classification	0.873	-0.3437	2	14
623	colony-stimulating factor	-0.1396	-0.0529	3	23
628	combination	0.3786	-0.7475	2	17
641	complications	0.1571	-0.6518	6	24
649	controlled clinical-trial	0.9618	-0.0832	2	20
652	controlled trial	0.7015	-0.6167	2	18
702	cyclophosphamide	0.1269	0.1317	5	66
708	cyclosporine	0.8106	-0.7532	2	20
711	cyclosporine-a	0.7256	0.3273	2	21
713	cystitis	-0.2364	0.9276	5	13
720	cytokine	-0.0775	0.6329	5	24
723	cytokines	0.3797	0.3689	3	40
735	damage	0.111	-0.3753	6	20
742	death	-0.8487	-0.3068	1	19
776	diagnosis	0.5361	-0.5583	2	12
798	disability	0.9688	-0.4075	2	24

799	disease	0.4927	-0.1819	2	69
813	dna	-0.7586	-0.6109	1	20
822	dna-damage	-1.0702	-0.0267	1	19
831	double-blind	0.9888	-0.3106	2	39
844	drugs	0.6038	-0.3386	2	23
856	efficacy	1.1072	-0.5783	2	13
887	endothelial-cells	0.1165	0.4418	4	48
890	endotoxin	-0.293	0.4917	3	16
928	escherichia-coli	-0.0613	0.0385	3	21
959	expression	-0.3835	0.1298	3	81
982	factor-alpha	-0.0943	0.3524	3	29
1046	fluorouracil	0.0701	-0.2043	3	9
1050	follow-up	0.7728	-0.2259	2	21
1059	free-radicals	-0.6702	-0.2757	1	43
1093	gene-expression	-0.532	0.2844	1	16
1096	generation	-0.1831	0.3256	4	36
1097	genes	-0.9011	-0.4908	1	22
1125	glutathione	-0.9391	-0.3784	1	25
1138	gm-csf	-0.1475	-0.2093	3	14
1155	growth	-0.5131	-0.2822	1	35
1254	human breast-cancer	-0.9186	0.1397	1	18
1271	human polymorphonuclear leukocytes	0.966	0.6287	4	18
1277	human-b	0.7739	0.8411	4	21
1290	human-neutrophils	0.6778	0.5824	4	25
1298	hydrogen-peroxide	-1.0335	-0.3727	1	30
1322	identification	-0.7658	-0.4659	1	15
1375	immunosuppression	0.3807	0.0446	3	14
1386	in-vitro	-0.6788	-0.402	1	20
1388	in-vivo	-0.0842	0.8124	5	26
1398	indomethacin	0.3683	0.7365	5	20
1421	induction	-0.7013	0.1718	1	45
1426	infection	-0.2771	0.3988	3	28
1431	inflammation	0.2468	0.258	5	99
1434	inflammatory bowel-disease	1.0343	-0.128	2	14
1450	inhibition	0.2137	0.5304	4	43
1456	injury	0.2154	0.4163	5	26
1470	interferon-gamma	-0.5807	0.5473	3	18
1478	interleukin-1	0.1143	0.7218	4	41
1490	interleukin-6	0.3032	-0.137	3	15
1528	invitro	0.1718	-0.0121	4	51
1529	invivo	0.1488	0.2979	3	26
1531	involvement	0.6193	0.3302	5	17
1612	leukemia	0.3177	-0.4678	2	10
1645	liposomes	0.268	0.1141	4	14
1648	liver	0.3345	-0.6707	2	12
1664	low-dose methotrexate	0.9428	0.0663	2	40
1673	lung	0.4116	-0.2795	2	25

1710	macrophages	0.1837	0.8332	4	22
1747	mechanisms	-0.1709	0.5854	5	37
1760	melanoma-cells	-0.6952	-0.7388	1	13
1784	messenger-rna	-0.3993	0.2455	1	14
1788	metabolism	-0.2089	0.1428	4	42
1792	metastasis	-0.5354	-0.6478	1	15
1796	methotrexate	0.6639	0.1124	4	76
1804	mice	-0.0225	0.4565	3	40
1835	molecular-cloning	-0.455	0.026	3	14
1841	monoclonal-antibody	-0.8497	0.3719	3	16
1860	mouse model	-0.2614	0.2397	3	12
1931	natural-history	0.7797	-0.3836	2	12
1934	necrosis-factor-alpha	0.423	0.4909	4	24
1960	neutropenia	-0.0177	0.1432	3	18
1968	neutrophils	0.5102	0.307	5	20
1975	nitric-oxide synthase	-0.5049	0.7088	3	17
1984	nod mice	-0.6689	0.5929	3	12
2074	oxidative stress	-0.7319	-0.21	1	28
2149	pharmacokinetics	-0.2682	-0.6191	1	11
2168	placebo	0.874	-0.4579	2	21
2169	placebo-controlled trial	1.1469	-0.1672	2	16
2204	polymorphonuclear leukocytes	0.3977	0.5858	5	28
2213	postmenopausal women	-0.6898	-0.1006	1	10
2237	prevention	-0.602	0.1681	3	17
2244	primary biliary-cirrhosis	0.6912	-0.4437	2	13
2253	prognostic factors	0.1442	-0.7323	2	14
2292	proteins	-0.3632	0.3187	5	27
2316	pulse methotrexate	0.7868	-0.5567	2	20
2347	radiation-therapy	0.0148	-0.6045	2	16
2389	rats	-0.4411	0.3982	5	39
2393	reactive oxygen species (ros)	-1.0693	-0.1724	1	37
2402	receptors	-0.09	0.5473	4	46
2435	release	0.051	-0.0117	4	32
2445	resistance	-0.8699	-0.2322	1	24
2453	responses	-0.2326	0.73	5	22
2462	retinoic acid	-0.7368	0.392	1	11
2469	rheumatoid arthritis	0.5922	-0.0812	2	50
2470	rheumatoid-arthritis	0.7899	0.1889	4	62
2527	serum	0.2808	-0.02	3	21
2631	sulfasalazine	0.7725	-0.0608	2	21
2636	superoxide	-0.8353	0.2468	1	21
2637	superoxide anion generation	0.9121	0.7963	4	18
2639	superoxide-dismutase	-0.8631	-0.1069	1	23
2650	surgery	-0.0292	-1.1229	6	8
2651	survival	0.2629	-0.3912	2	18
2680	systemic lupus-erythematosus	0.7702	0.4559	4	25
2691	t-cells	-0.4983	0.57	3	22

2728	therapy	0.5045	-0.4443	2	58
2744	tissues	-0.647	0.2734	1	16
2760	toxicity	-0.184	-0.5255	1	39
2761	trabeculectomy	0.0233	-1.1668	6	3
2797	trial	1.0083	-0.5993	2	18
2812	tuberculosis	-0.0227	-0.7957	6	12
2818	tumor necrosis factor	-0.0955	0.1967	3	27
2824	tumor-cells	-1.132	-0.4818	1	17
2827	tumor-necrosis-factor	0.0288	0.2851	3	61
2842	ulcerative-colitis	1.1181	-0.0474	2	11
2851	urinary bladder	-0.4545	0.8922	5	12
2867	uveitis	0.2119	-0.9758	6	13
2880	vascular-permeability	0.2736	0.6812	5	22
2882	vasculitis	0.9189	-0.1764	2	8

Total link strength	Occurrences	Avg. pub. year	Avg. citations	Avg. norm. citations
22	15	1995.0667	41.9333	0.9377
29	5	1996.6	72.8	1.7113
19	7	1994.1429	66	1.1116
44	11	1996.2727	45.2727	1.0262
46	6	1996.1667	140	3.441
28	7	1996.4286	73.4286	1.4296
15	5	1995	34	0.6634
36	5	1995.4	196.2	4.6235
17	5	1995.8	53.2	1.1922
35	9	1997.1111	39.3333	0.8697
47	14	1998.1429	189.8571	3.0535
18	6	1995	67.5	2.0607
29	6	1996.8333	46.8333	1.1581
23	8	1995.75	42.75	0.9722
36	9	1995.8889	27	0.6565
21	5	1998.6	68.6	1.6024
15	5	1996.4	43.8	1.0445
16	7	1997.2857	126.2857	2.7994
9	5	1997.6	187.4	2.8378
94	33	1997.0606	94.8485	1.9093
23	7	1996.1429	57.1429	1.3251
24	6	1993.8333	74.8333	1.3306
58	21	1995.3333	48	1.0871
21	6	1995.8333	105	1.7149
26	10	1996	45.8	0.991
99	28	1996	69.6786	1.5745
15	7	1996.8571	51.2857	1.1956
120	46	1995.5435	38.2391	0.8385
9	6	1996.6667	29.8333	0.6907
16	7	1997.1429	61.8571	1.3277
21	7	1996.5714	112.7143	1.7324
28	10	1994.6	68.5	1.664
21	5	1997.2	38.8	0.9693
27	9	1996.5556	48.1111	1.1475
23	6	1995.8333	141.3333	2.5487
22	7	1997.1429	40.7143	0.9052
119	38	1995.3158	47.5263	1.1502
31	8	1996	40.5	0.8716
27	7	1997.2857	44.4286	1.07
25	6	1996.3333	32	0.6316
27	6	1995.6667	94.5	2.2759
54	11	1996.4545	99.2727	2.3253
22	7	1997	54.7143	1.1941
33	9	1998.1111	37.4444	0.8407
14	8	1996	32	0.6079
29	5	1997.2	117.4	1.8632

115	35	1996.2571	59.6	1.1067
21	5	1996.8	197.6	3.0298
25	5	1998.4	100.4	2.2607
76	18	1997.1111	49.8889	1.1127
30	10	1994.7	70.9	1.1621
17	6	1996.3333	102.3333	1.5774
74	16	1996	93.8125	2.3943
17	5	1994.6	38.6	0.8418
24	5	1995.4	24.2	0.6593
148	39	1996.5128	83.4615	1.8446
42	10	1995.9	55.8	1.3812
10	5	1996.4	30	0.7098
23	6	1997.6667	50.8333	1.0475
70	16	1996.75	64.625	1.4473
19	10	1997.1	121	2.7141
45	8	1996.25	58.875	1.027
24	7	1997.4286	79.4286	1.8093
31	6	1998.5	59.1667	1.3113
16	5	1993.6	34.4	0.8476
46	14	1997.1429	46.2143	1.0636
21	7	1998.7143	131	2.893
26	5	1994.8	154	2.7068
36	5	1995.4	196.2	4.6235
30	6	1994	122	3.2016
40	13	1995.7692	110.2308	1.8201
17	6	1996.3333	92.5	1.6845
16	5	1996.6	48.6	1.1885
22	9	1998.5556	58.4444	1.3041
40	11	1997.5455	59.4545	1.4233
27	6	1994.3333	27	0.7334
62	16	1995.25	60.5	1.3422
33	8	1995.875	35.25	0.7718
252	73	1995.4521	66.4384	1.4802
22	7	1997	44	0.9192
71	16	1995.875	61.625	1.6573
32	8	1993.875	35.25	0.8791
30	7	1996.4286	40.5714	1.0565
58	9	1995.1111	26.5556	0.5909
18	5	1996.6	36	0.919
78	18	1995.9444	39.9444	0.736
31	10	1993.1	35.9	0.8203
21	7	1997.7143	70.7143	1.4557
14	5	1995.2	28.8	0.4196
17	5	1995.6	25.2	0.5238
13	5	1995.4	52	1.0298
75	16	1996.9375	57.875	1.3657
27	10	1994.9	53.1	1.1358

28	7	1996.2857	47.2857	0.9423
52	9	1996.6667	44.3333	0.8095
15	5	1997	103	2.3528
14	8	1996.375	69.5	1.6295
51	12	1995.4167	74.5833	1.8727
19	6	1996.3333	63.3333	1.5128
208	49	1996.8776	68.3878	1.4735
61	18	1995.7778	60.1667	1.4248
15	8	1996.375	92.875	2.2206
17	7	1996	48.5714	1.0674
14	5	1996.2	76.4	2.1927
14	5	1997.2	58.4	1.1944
27	9	1997	90	2.0619
24	5	1995.6	31.4	0.6007
23	7	1994.4286	82.1429	2.0832
21	5	1998	53.6	1.2281
20	5	1997.2	66.4	1.5976
36	10	1998.2	59.9	1.3632
13	5	1997.8	47	1.0078
29	6	1995.8333	49.6667	0.9019
29	8	1997.125	31.75	0.7724
36	8	1993.25	96.875	2.4156
16	5	1997.4	62.8	1.4791
21	5	1997.2	50	1.1675
15	6	1995.6667	51	0.9978
17	6	1995.5	42.8333	0.8586
30	8	1995.875	46.5	1.1637
29	6	1996.3333	43.3333	0.8887
23	9	1995.7778	48.1111	1.1527
64	20	1997.05	39.9	0.9091
52	13	1997.7692	123.3846	2.298
66	16	1995.75	54.8125	1.2113
42	8	1995.5	53.125	1.164
28	7	1996.7143	176	2.9852
29	8	1995.375	31.75	0.7162
13	5	1995	58.4	1.4458
94	21	1996.7143	51.7619	1.1591
138	33	1996.303	66.0303	1.5975
26	7	1994.4286	27.7143	0.6645
29	5	1998	92	2.0911
25	5	1996	48.6	1.089
33	6	1994.3333	251.6667	5.265
27	8	1996.5	53.5	1.2386
11	8	1995.5	35.25	0.8613
20	6	1997.3333	66	1.3659
34	8	1994.75	101	2.2106
32	12	1996.8333	77.75	1.9552

111	36	1996.5556	59.9444	1.3559
16	7	1996.7143	57.2857	1.4504
48	12	1996.6667	55.8333	1.2228
7	7	1996.1429	34.7143	0.8315
25	7	1996.4286	24.8571	0.537
12	6	1995.8333	40.3333	0.8934
35	9	1995.1111	38.6667	0.9984
26	7	1997.1429	58.8571	1.3063
105	28	1996.7143	85.6429	1.9758
18	6	1997.3333	38.8333	0.9384
23	7	1997.5714	41.8571	0.9824
13	5	1995.4	53.4	1.2991
29	6	1995.1667	49.1667	1.1643
9	7	1993.8571	66.7143	1.4151
