# Supplementary material for: A Bibliometric Analysis of Cyclophosphamide, Methotrexate, and Fluorouracil Breast Cancer Treatments: Implication for the Role of Inflammation in Cognitive Dysfunction
Source: Front Mol Biosci. 2021 Aug 20;8:683389. doi: 10.3389/fmolb.2021.683389 (PMC8417522; doi:10.3389/fmolb.2021.683389)
Supplement: Supplementary file 5 [file DataSheet1.PDF]

Materials and Methods

Data source and search strategy

This bibliometric study analyzed research articles related to inflammation, chemobrain, cyclophosphamide, methotrexate, and fluorouracil published from January 1990 through December 2019. The study period was subdivided into three decades (1990s, 2000s, and 2010s). The Web of Science online database (Clarivate Analytics, Philadelphia, PA) was accessed April 2020. Two sets of separate topic searches (TS) were performed under the advanced search option. For methodology, please refer to Mcelroy et al and Simmons et al.

The search term included:

Set 1: TS = ("inflammation")

Set 2: TS= ("chemobrain" or "chemotherapy" or "breast cancer" or "methotrexate" or "cyclophosphamide" or "fluorouracil")

Data analysis and presentation

For bibliometric analysis, data were imported into VOSviewer version 1.6.14 (Centre for Science and Technology Studies, Leiden University, Leiden, The Netherlands). Supplemental **Table S1** for the final thesaurus used.

The software performs keyword analysis by identifying keywords in the titles and abstracts of publications and relating them to documents in which they occur together (co-occurrence analysis). A threshold was placed by the user to sort terms by the minimum times the word occurred in titles and abstracts of the publications. The minimum threshold of keywords for each of the time periods was set as followed: 1990s = 5x, 2000s = 10x, 2010s = 25x, 2020-2021=20x. For further details, see Mcelroy et al and Simmons et al [14-15].

For 1990-2019, data from journals and countries' publication and citation data were extracted on April 6, 2020. For citation analysis, the 29,222 citations that occurred without self-citation were used.

1. McElroy T, Allen AR. A Bibliometric Review of Publications on Oxidative Stress and Chemobrain: 1990-2019. Antioxidants (Basel). 2020;9(5):439. Published 2020 May 18. doi:10.3390/antiox9050439

2. Simmons P, McElroy T, Allen AR. A Bibliometric Review of Artificial Extracellular Matrices Based on Tissue Engineering Technology Literature: 1990 through 2019. Materials (Basel). 2020;13(13):2891. Published 2020 Jun 27. doi:10.3390/ma13132891
