# Supplementary material for: A Bibliometric Analysis of Cyclophosphamide, Methotrexate, and Fluorouracil Breast Cancer Treatments: Implication for the Role of Inflammation in Cognitive Dysfunction
Source: Front Mol Biosci. 2021 Aug 20;8:683389. doi: 10.3389/fmolb.2021.683389 (PMC8417522; doi:10.3389/fmolb.2021.683389)
Supplement: Supplementary file 6 [file DataSheet5.PDF]

id	label	cluster	Links	Total link strength
30	1,25-dihydroxyvitamin d-3	2	49	83
48	15-deoxy-delta(12,14)-prostaglandin j(2)	3	50	93
155	5-fluorouracil	6	127	235
224	abatacept	1	52	89
259	accumulation	1	54	65
281	acid	2	140	210
343	activated protein-kinase	3	121	222
344	activated protein-kinases	2	65	85
348	activated receptor-gamma	3	63	102
353	activated-receptor-gamma	3	56	91
357	activation	2	388	1691
409	acute lymphoblastic-leukemia	2	67	87
415	acute myeloid-leukemia	3	57	74
425	acute promyelocytic leukemia	2	84	156
439	acute-phase response	6	52	84
445	adalimumab	1	62	125
467	adenocarcinoma	2	83	122
477	adenosine	5	68	116
484	adenosine release	1	53	90
496	adhesion	4	53	77
503	adhesion molecules	4	80	118
519	adipose-tissue	3	66	104
524	adjuvant arthritis	1	58	103
525	adjuvant chemotherapy	4	53	66
532	adjuvant-induced arthritis	1	44	62
551	adriamycin	2	117	229
602	afferent neurons	5	34	65
627	agents	2	89	111
634	aging	3	62	78
664	airway inflammation	5	39	45
673	akt	3	52	73
679	albumin	6	38	60
760	alpha monoclonal-antibody	1	74	182
803	alpha-tocopherol	2	59	69
832	alveolar macrophages	1	81	129
838	alzheimers-disease	3	63	79
891	anakinra	1	70	134
895	analogs	2	37	45
909	anca	1	34	57
927	angiogenesis	4	286	1006
963	ankylosing spondylitis	1	41	73
964	ankylosing-spondylitis	1	68	134
977	antagonist	4	46	62
981	antagonists	5	49	55
990	anthracyclines	2	61	101
1079	antibiotics	1	34	42

1080	antibodies	1	97	127
1081	antibody	1	87	125
1098	anticancer drugs	2	79	120
1178	antineutrophil cytoplasmic antibodies	1	24	41
1185	antioxidant activity	2	54	69
1191	antioxidant enzymes	2	105	218
1200	antioxidant status	2	61	110
1204	antioxidants	2	243	787
1244	antitumor necrosis factor	1	59	112
1248	antitumor-activity	2	124	198
1272	ap-1	2	45	75
1297	apoptosis	2	439	3151
1309	apoptotic cells	4	44	64
1361	arsenic trioxide	2	106	217
1384	arthritis	1	182	361
1433	aspirin	3	65	89
1434	aspirin use	3	39	81
1435	assay	2	51	64
1439	association	3	175	372
1443	asthma	1	97	134
1457	atherosclerosis	3	122	237
1502	autoantibodies	1	53	66
1522	autoimmunity	1	86	124
1530	autophagy	2	50	67
1550	azathioprine	1	62	102
1572	b-cells	1	39	43
1636	base excision-repair	3	41	54
1654	bax	2	63	104
1667	bcl-2	2	122	320
1669	bcl-2 family	2	67	96
1707	behcets-disease	1	40	62
1741	beta-carotene	2	61	90
1743	beta-catenin	3	58	77
1810	binding-sites	3	45	58
1860	biomarker	6	65	94
1863	biomarkers	1	67	104
1871	biopsy	1	50	59
1900	bladder	5	95	250
1919	bleomycin	1	62	84
1926	blood	1	85	112
1940	blood-brain-barrier	5	52	57
1978	bone	1	70	96
2009	bone-marrow	4	85	116
2018	bone-marrow transplantation	1	43	58
2021	bone-marrow-transplantation	1	99	166
2071	breast	6	66	83
2103	breast-cancer	3	555	5016

2105	breast-cancer cells	2	309	1165
2108	breast-cancer metastasis	4	42	60
2109	breast-cancer patients	3	57	63
2111	breast-cancer risk	3	90	144
2115	breast-cancer-cells	2	54	76
2139	bronchoalveolar lavage	1	44	55
2170	buthionine sulfoximine	2	55	83
2198	c-fos	5	38	74
2217	c-reactive protein	6	184	463
2301	cancer	3	483	2235
2343	cancer-cells	2	207	509
2344	cancer-chemotherapy	2	52	62
2347	cancer-patients	1	68	110
2355	cancer-therapy	2	68	97
2383	capsaicin	5	59	103
2411	carcinogenesis	3	179	405
2415	carcinoma	4	288	856
2431	carcinoma-cells	2	163	408
2432	carcinomas	4	78	129
2452	cardiomyopathy	2	51	71
2457	cardiotoxicity	2	87	154
2460	cardiovascular disease	3	59	89
2473	cardiovascular-disease	3	86	169
2506	caspase activation	2	69	107
2511	caspase-3	2	66	108
2522	caspases	2	124	289
2531	catalase	2	77	127
2670	celecoxib	3	77	114
2680	cell carcinoma	3	34	44
2697	cell lung-cancer	6	120	212
2726	cell-cycle	2	103	182
2727	cell-cycle arrest	2	55	91
2731	cell-death	2	158	349
2737	cell-growth	2	49	63
2740	cell-lines	2	132	214
2747	cell-proliferation	3	92	146
2753	cells	4	370	1304
2789	central-nervous-system	3	95	136
2792	ceramide	2	42	64
2866	chemiluminescence	2	59	73
2876	chemokine	4	62	102
2892	chemokine receptors	4	50	77
2894	chemokines	4	90	160
2896	chemoprevention	3	127	294
2923	chemotherapy	6	434	1940
2940	childhood	1	47	67
2951	children	1	115	251

2952	chimeric monoclonal-antibody	1	56	108
3062	chronic inflammation	4	99	149
3072	chronic lymphocytic-leukemia	2	78	130
3141	cisplatin	2	163	383
3167	classification	1	99	149
3205	clinical trials	1	69	82
3217	clinical-trial	1	66	94
3219	clinical-trials	1	61	94
3272	cohort	1	67	99
3277	colitis	1	82	115
3289	collagen-induced arthritis	1	159	436
3299	colon	5	38	47
3307	colon-cancer	3	163	341
3308	colon-cancer cells	3	66	97
3324	colony-stimulating factor	4	141	229
3337	colorectal-cancer	3	262	759
3345	combination	1	102	165
3348	combination therapy	1	86	211
3403	complications	1	85	130
3422	concomitant methotrexate	1	59	125
3475	constitutive activation	3	54	83
3513	controlled trial	1	75	140
3516	controlled-trial	1	59	116
3534	copper	2	77	125
3569	coronary-heart-disease	3	80	151
3584	corticosteroids	1	108	171
3613	cox-2	3	113	220
3648	criteria	1	38	54
3654	crohn's disease	1	68	134
3656	crohns-disease	1	126	292
3688	crystal-structure	3	53	67
3695	ct	1	34	45
3729	curcumin	2	72	114
3775	cyclase-activating polypeptide	5	35	85
3777	cycle arrest	2	80	118
3806	cyclooxygenase	3	69	152
3810	cyclooxygenase-2	3	118	263
3814	cyclooxygenase-2 expression	3	91	157
3823	cyclopentenone prostaglandins	3	48	89
3826	cyclophosphamide	1	291	894
3830	cyclophosphamide cystitis	5	45	140
3834	cyclophosphamide-induced cystitis	5	38	65
3840	cyclosporine	1	81	142
3843	cyclosporine-a	1	95	144
3869	cystitis	5	78	254
3893	cytochrome-c	2	118	292
3894	cytochrome-c release	2	105	263

3911	cytokine	4	181	356
3924	cytokine production	1	54	79
3932	cytokines	1	307	850
3980	cytotoxicity	2	122	280
4013	damage	2	184	387
4041	death	2	210	732
4077	degradation	6	52	63
4120	dendritic cells	4	122	230
4160	depression	1	51	81
4184	destruction	1	56	85
4203	dexamethasone	6	83	133
4215	diabetes	3	56	82
4230	diagnosis	1	153	263
4266	diet	3	44	58
4308	differential expression	3	58	83
4316	differentiation	2	159	334
4360	disability	1	44	78
4364	disease	1	329	983
4381	disease-activity	1	104	219
4394	diseases	1	76	111
4432	dna	2	132	212
4466	dna-binding	4	56	86
4470	dna-damage	2	202	545
4476	dna-repair	3	58	89
4483	docetaxel	6	50	60
4484	docosahexaenoic acid	3	57	86
4507	dorsal-root ganglia	5	30	75
4533	double-blind	1	257	1080
4537	down-regulation	2	150	313
4539	doxorubicin	2	152	365
4578	drug-delivery	6	60	77
4580	drug-induced apoptosis	2	65	95
4585	drug-resistance	2	120	240
4589	drugs	1	117	161
4628	dysfunction	2	57	67
4643	e-cadherin	3	37	55
4646	e-selectin	4	68	107
4665	early rheumatoid-arthritis	1	53	87
4702	efficacy	1	173	500
4730	eicosapentaenoic acid	3	59	79
4808	emt	3	48	70
4864	endothelial cells	3	67	92
4867	endothelial growth-factor	4	242	655
4879	endothelial-cells	4	192	394
4888	endothelium	4	78	116
4958	enzymes	2	55	81
4972	epidemiology	1	94	135

4983	epidermal-growth-factor	3	88	143
5007	epithelial-cells	3	194	459
5081	escherichia-coli	1	56	60
5104	estradiol	3	67	107
5112	estrogen	3	137	240
5126	estrogen-receptor	3	126	213
5141	etanercept	1	131	479
5206	experience	1	41	56
5212	experimental autoimmune encephalomyelitis	3	38	46
5235	expression	4	514	2871
5265	extracellular-matrix	4	77	110
5297	f-18-fdg pet	1	29	62
5337	factor receptor	4	46	59
5356	factor-alpha	1	197	458
5373	factor-i	4	50	63
5377	factor-kappa-b	2	184	317
5390	familial adenomatous polyposis	3	56	137
5395	family	2	75	104
5427	fatigue	1	54	86
5443	fatty-acids	3	35	55
5458	fdg-pet	1	42	100
5463	features	1	50	69
5505	fever	1	40	57
5532	fibroblasts	6	93	124
5543	fibrosis	1	55	64
5564	fish-oil	3	37	52
5585	flavonoids	2	97	164
5635	fluorouracil	6	100	185
5666	follow-up	1	154	294
5724	free-radicals	2	224	621
5775	fusion protein	1	76	172
5872	gastric-cancer	3	159	290
5932	gene polymorphisms	1	53	68
5938	gene therapy	1	55	67
5944	gene-expression	3	393	1396
5957	gene-related peptide	5	35	70
5964	generation	2	101	161
5966	genes	2	213	489
5976	genetic polymorphisms	3	71	123
5988	genetics	1	55	68
5989	genistein	3	56	77
6031	giant-cell arteritis	1	41	73
6073	glomerulonephritis	1	47	64
6090	glucocorticoids	1	73	102
6092	glucose	2	44	61
6115	glutathione	2	189	482
6131	glutathione-peroxidase	2	116	231

6237	green tea	3	68	93
6257	growth	2	278	884
6275	growth-factor	4	188	378
6277	growth-factor receptor	3	85	139
6283	growth-factor-beta	2	111	159
6286	growth-factor-i	3	50	79
6291	growth-inhibition	3	85	133
6310	guidelines	1	45	54
6386	head	1	42	54
6389	head and neck cancer	1	50	70
6420	heart-disease	3	48	70
6421	heart-failure	1	70	94
6549	hepatocellular-carcinoma	3	129	231
6616	high-dose chemotherapy	1	49	72
6702	hl-60 cells	2	66	108
6728	hodgkins-disease	1	51	70
6759	hormone replacement therapy	3	74	119
6830	human breast-cancer	3	281	778
6841	human colon-cancer	3	60	123
6948	human prostate-cancer	3	68	108
6977	human-breast-cancer	3	45	80
6981	human-immunodeficiency-virus	1	29	34
7031	hydrogen-peroxide	2	249	838
7051	hyperalgesia	5	43	68
7095	hypoxia	4	100	152
7142	identification	6	151	246
7162	ifn-gamma	4	73	110
7201	il-10	4	78	99
7221	il-6	4	93	163
7290	immune-response	4	81	116
7292	immune-responses	4	59	87
7294	immune-system	4	67	87
7295	immunity	4	67	96
7325	immunohistochemistry	5	71	103
7333	immunomodulation	1	56	66
7350	immunosuppression	1	104	160
7355	immunosuppressive therapy	1	37	45
7364	immunotherapy	4	113	164
7366	impact	1	41	61
7372	improvement	1	53	82
7388	in-situ	4	51	71
7393	in-vitro	2	388	1390
7401	in-vivo	4	356	1031
7430	increased risk	3	60	86
7463	induced apoptosis	2	248	851
7477	induced cytotoxicity	2	54	70
7495	induced hemorrhagic cystitis	5	70	160

7513	induced oxidative stress	2	71	103
7551	induction	2	278	842
7566	infection	1	182	346
7569	infections	1	49	61
7576	infiltration	1	43	56
7579	inflammation	4	610	5321
7594	inflammatory bowel disease	1	50	81
7603	inflammatory cytokines	2	47	58
7620	inflammatory-bowel-disease	1	118	194
7623	infliximab	1	189	759
7639	inhibition	2	325	1059
7648	inhibitors	4	171	331
7673	injury	2	116	191
7680	innate immunity	4	50	71
7714	insulin-resistance	3	82	124
7745	intercellular-adhesion molecule-1	4	51	75
7756	interferon-alpha	1	72	99
7761	interferon-gamma	4	112	179
7778	interleukin-1	1	64	90
7784	interleukin-1 receptor antagonist	1	114	307
7808	interleukin-6	1	177	387
7816	interleukin-8	1	89	127
7840	interstitial cystitis	5	70	241
7995	invasion	4	124	262
8018	involvement	5	164	339
8025	ionizing-radiation	2	109	174
8030	irinotecan	6	36	55
8036	iron	2	87	139
8053	irradiation	1	83	113
8064	ischemia-reperfusion injury	2	59	74
8134	jnk	2	63	99
8139	joint damage	1	49	71
8140	joint destruction	1	57	87
8143	joint inflammation	1	59	100
8154	juvenile idiopathic arthritis	1	68	158
8158	juvenile rheumatoid-arthritis	1	37	54
8178	kappa-b	2	110	166
8179	kappa-b activation	6	63	81
8216	kinase	2	137	291
8366	leflunomide	1	67	149
8387	leptin	6	51	75
8392	lesions	1	62	90
8405	leucovorin	6	49	85
8406	leukemia	2	156	271
8412	leukemia-cells	2	90	150
8425	leukocytes	1	70	88
8473	ligands	3	100	155

8499	lines	2	126	246
8532	lipid-peroxidation	2	193	529
8565	liver	2	90	151
8610	localization	2	52	67
8623	long-term	1	81	127
8656	low-density-lipoprotein	3	61	88
8660	low-dose cyclosporine	1	39	58
8661	low-dose methotrexate	1	153	313
8698	lung	1	84	118
8710	lung injury	1	44	69
8720	lung-cancer	3	223	454
8748	lymph-node metastasis	4	50	69
8754	lymphangiogenesis	4	34	64
8792	lymphocytes	4	97	124
8805	lymphoma	1	74	92
8862	macrophage	4	65	94
8880	macrophages	4	181	396
8929	malignancy	1	67	92
8951	malignant-melanoma	2	52	60
8954	malondialdehyde	2	73	111
8982	mammary epithelial-cells	3	62	95
8995	mammary-gland	3	55	69
9000	mammary-tumors	4	48	73
9005	management	1	133	262
9016	manifestations	1	50	73
9023	map kinase	3	82	109
9034	marker	6	43	57
9037	markers	1	63	88
9054	mass-spectrometry	6	36	51
9062	mast-cells	4	38	60
9085	matrix metalloproteinases	4	108	215
9106	mcf-7 cells	2	109	209
9143	mechanisms	2	326	916
9151	mediated apoptosis	2	102	179
9206	melanoma-cells	2	133	207
9207	melatonin	2	64	120
9216	membrane	2	63	75
9275	messenger-rna	5	171	303
9291	metabolism	2	155	319
9313	metastases	1	63	78
9314	metastasis	4	240	802
9319	metastatic breast-cancer	6	60	81
9321	metastatic colorectal-cancer	6	44	65
9339	methotrexate	1	381	2352
9350	methotrexate therapy	1	51	95
9381	methylprednisolone	1	47	79
9402	mice	5	242	612

9410	microarray	6	59	77
9423	microenvironment	4	61	114
9441	microsatellite instability	3	40	59
9446	microscopic polyangiitis	1	32	50
9473	migration	4	111	211
9509	mitochondrial	2	203	716
9530	mitochondrial membrane potential	2	69	106
9534	mitochondrial permeability transition	2	73	116
9540	mitochondrial targeting sequence	3	41	76
9555	mitomycin-c	6	47	61
9582	mnsod	2	142	371
9598	model	5	138	266
9609	modifying antirheumatic drugs	1	62	123
9611	modulation	1	82	122
9641	molecular-cloning	4	42	49
9666	monoclonal-antibody	1	169	476
9676	monocyte chemoattractant protein-1	4	75	109
9681	monocytes	1	74	106
9717	mortality	1	126	276
9737	mouse model	5	148	249
9757	mri	1	48	73
9806	mucositis	1	75	136
9815	multicenter	1	59	95
9821	multidrug-resistance	2	121	246
9846	multiple-myeloma	4	101	145
9848	multiple-sclerosis	1	49	62
9904	mutations	3	78	142
9916	mycobacterium-tuberculosis	1	45	59
9922	mycophenolate-mofetil	1	68	136
9954	myeloperoxidase	3	58	92
9970	myocardial-infarction	3	67	95
10004	n-acetylcysteine	2	77	105
10012	n-terminal kinase	2	81	144
10032	nadph oxidase	2	75	101
10087	necrosis	2	101	167
10092	necrosis-factor	4	74	97
10097	necrosis-factor-alpha	1	330	1055
10124	neoplasia	6	42	52
10140	nephrotoxicity	2	50	71
10145	nerve growth-factor	5	56	208
10176	neuroblastoma	2	69	95
10245	neutropenia	1	49	74
10252	neutrophil	1	60	76
10268	neutrophils	1	94	142
10280	nf-kappa-b	3	391	1799
10322	nitric-oxide	2	297	860
10327	nitric-oxide production	3	48	75

10330	nitric-oxide synthase	5	255	622
10389	non-hodgkins-lymphoma	1	77	103
10435	nonsteroidal antiinflammatory drugs	3	205	512
10560	obesity	3	66	115
10715	oral mucositis	1	55	86
10775	osteoarthritis	6	47	71
10827	ovarian-cancer	6	140	250
10841	overexpression	2	131	246
10866	oxidative damage	2	56	77
10869	oxidative dna-damage	3	81	138
10876	oxidative stress	2	422	2542
10896	oxygen	2	63	76
10914	p-glycoprotein	2	108	199
10937	p38 mapk	2	64	89
10947	p53	2	178	396
10972	paclitaxel	2	64	104
10983	pain	5	101	191
11019	pancreatic-cancer	3	129	228
11100	pathogenesis	1	156	323
11110	pathways	5	173	377
11111	patient	1	41	51
11179	peptide	5	36	48
11202	performance status	6	32	67
11251	peripheral-blood	1	91	125
11275	permeability transition pore	2	79	150
11292	peroxynitrite	2	78	148
11305	pet	1	41	71
11328	pharmacokinetics	1	82	116
11348	phase-i	6	82	118
11349	phase-i trial	3	57	72
11351	phase-ii trial	6	118	205
11360	phenotype	3	55	71
11381	phosphatidylinositol 3-kinase	3	48	62
11403	phosphorylation	2	142	254
11414	photodynamic therapy	2	95	141
11436	physical-activity	3	52	82
11506	placebo	1	79	228
11508	placebo-controlled trial	1	147	398
11520	plasma	1	138	234
11563	plasminogen-activator	4	58	82
11643	poly(adp-ribose) polymerase	2	66	85
11681	polymerase-chain-reaction	1	34	43
11690	polymorphisms	3	144	310
11714	polyunsaturated fatty-acids	3	63	80
11720	population	1	42	69
11745	positron emission tomography	1	34	45
11747	positron-emission-tomography	1	76	174

11771	postmenopausal women	3	117	209
11827	ppar-gamma	3	76	180
11896	prevalence	1	59	84
11899	prevention	3	193	357
11997	prognosis	4	139	266
12002	prognostic factors	1	47	59
12014	prognostic-significance	3	69	92
12017	programmed cell-death	2	66	86
12019	progression	4	239	605
12039	proliferation	2	204	441
12060	promoter	6	53	66
12112	prostaglandin e-2	3	57	83
12130	prostaglandins	3	67	90
12134	prostate	5	84	114
12139	prostate-cancer	3	260	696
12140	prostate-cancer cells	2	87	134
12168	protects	2	66	89
12211	protein-kinase	2	122	205
12219	protein-kinase-c	3	105	199
12237	proteins	2	245	610
12251	proteomics	6	62	83
12296	psoriasis	1	88	139
12298	psoriatic arthritis	1	63	124
12300	psoriatic-arthritis	1	56	99
12343	pulmonary fibrosis	1	55	70
12369	pulse cyclophosphamide	1	46	74
12414	quality of life	1	55	74
12415	quality-of-life	1	121	224
12432	quercetin	2	61	88
12460	radiation	1	144	248
12478	radiation-therapy	1	173	369
12503	radiographic progression	1	57	96
12539	randomized controlled-trial	3	94	137
12541	randomized trial	1	52	71
12543	randomized-trial	1	119	176
12602	rat urinary-bladder	5	34	80
12607	rat-liver	2	49	59
12613	rats	5	236	539
12633	reactive oxygen	2	151	323
12637	reactive oxygen species (ros)	2	365	1614
12712	receptors	4	239	592
12751	recurrence	1	58	76
12781	redox regulation	2	50	67
12850	regulatory t-cells	4	81	126
12872	release	1	112	178
12878	remission	1	47	79
12906	renal-cell carcinoma	3	60	88

12917	repair	3	58	76
12951	resistance	2	144	262
12983	respiratory-distress-syndrome	1	40	49
12995	responses	4	77	110
13006	resveratrol	2	66	114
13023	retinoic acid	3	61	92
13070	rheumatoid arthritis	1	268	1215
13074	rheumatoid-arthritis	1	326	1334
13123	risk	3	216	571
13125	risk factor	1	45	53
13129	risk-factors	1	78	118
13130	rituximab	1	71	112
13157	ros	2	199	570
13221	safety	1	101	246
13253	sarcoidosis	1	34	48
13425	sensitivity	2	86	137
13436	sensory neurons	5	49	92
13442	sepsis	1	47	66
13483	serum	6	118	188
13522	serum-levels	4	61	104
13605	signal-regulated kinase	2	73	101
13610	signal-transduction	3	141	263
13618	signaling pathways	2	136	275
13685	skeletal-muscle	3	48	59
13686	skin	1	54	68
13749	smoking	3	51	87
13757	smooth-muscle cells	3	44	52
13760	smooth-muscle-cells	3	97	143
13809	solid tumors	6	59	84
13910	spinal-cord	5	58	127
13954	squamous-cell carcinoma	3	142	248
14018	stem-cell transplantation	1	65	92
14019	stem-cells	4	48	61
14081	stress	2	152	255
14098	stromal cells	4	50	70
14153	substance-p	5	57	81
14174	sulfasalazine	1	85	167
14199	superoxide	2	104	193
14207	superoxide-dismutase	2	182	461
14226	suppression	4	106	166
14247	surgery	6	97	159
14261	survival	6	235	616
14269	susceptibility	3	122	210
14326	synovial tissue	1	46	67
14330	synovial-fluid	1	68	124
14333	synovitis	1	40	67
14334	synthase	1	67	90

14346	system	4	73	99
14357	systemic inflammatory response	6	39	65
14362	systemic lupus erythematosus	1	54	66
14368	systemic vasculitis	1	36	70
14369	systemic-lupus-erythematosus	1	107	176
14398	t-cells	1	216	471
14403	t-lymphocytes	4	76	110
14425	tamoxifen	2	128	211
14533	terminal differentiation	3	50	73
14567	tgf-beta	4	88	118
14610	therapy	1	406	1429
14637	thioredoxin	2	62	90
14661	thrombosis	1	37	45
14753	tissues	1	148	261
14759	tnf	1	67	103
14760	tnf alpha	1	301	902
14856	toxicity	1	203	404
14899	transcription	3	112	202
14909	transcription factors	2	167	405
14912	transcriptional activation	4	45	56
14913	transcriptional activity	4	44	65
14942	transformation	2	54	67
14957	transgenic mice	4	119	191
14994	transplantation	1	83	120
15011	treatment	1	144	264
15023	trial	1	184	383
15094	tuberculosis	1	87	136
15112	tumor angiogenesis	4	91	147
15143	tumor necrosis factor	1	103	171
15151	tumor necrosis factor-alpha	1	96	148
15155	tumor progression	3	89	154
15178	tumor-associated macrophages	4	99	184
15187	tumor-cells	2	154	333
15193	tumor-growth	4	130	252
15202	tumor-necrosis-factor	1	349	1108
15217	tumorigenesis	3	95	145
15219	tumors	4	219	534
15269	tyrosine phosphorylation	3	63	90
15298	ulcerative colitis	1	56	78
15300	ulcerative-colitis	1	74	131
15303	ultrasonography	1	36	49
15332	united-states	3	63	88
15340	up-regulation	5	170	361
15364	urinary bladder	5	46	89
15374	urinary-bladder	5	70	180
15402	urothelium	5	46	83
15427	uveitis	1	94	194

15498	vasculitis	1	73	156
15520	vegf	4	79	123
15527	vegf-c	4	30	58
15553	versus-host-disease	1	56	86
15596	visceral pain	5	37	105
15625	vitamin-c	2	58	78
15633	vitamin-e	2	94	166
15700	wegener's granulomatosis	1	46	92
15703	wegeners-granulomatosis	1	42	58
15707	weight-loss	6	40	71
15739	women	1	131	243
15824	zinc	2	51	75

Occurrences	Avg. pub. year	Avg. citations	Avg. norm. citations
12	2004.8333	33.4167	0.5403
14	2005.6429	106.2143	1.5147
45	2005.2444	40.8667	0.571
10	2006.9	25.8	0.3719
12	2004.5833	39.6667	0.5788
30	2005.6	55.1333	0.7737
32	2005.9062	162.4375	2.4159
12	2005.9167	119.6667	1.7451
14	2006.7857	101.1429	1.4467
13	2007	46.0769	0.6963
248	2006.0202	70.3065	1.0609
17	2006.7059	55.3529	0.8477
13	2006.0769	167.6923	2.4811
22	2004.2727	62.1818	0.8284
16	2005.875	123.25	1.9063
13	2006.1538	35.7692	0.5425
18	2005.9444	41.8333	0.6465
16	2004.75	34.5625	0.5186
13	2003.2308	38.6154	0.5799
10	2005.3	35.8	0.5711
20	2003.55	65.25	0.9147
17	2006.6471	75.2353	1.1221
16	2004.75	34.625	0.5233
14	2006.8571	49.7857	0.7282
10	2005.2	59.9	1.0239
29	2005.5517	37.4828	0.6118
10	2006.3	38.1	0.5794
15	2005.2	35	0.4814
10	2006.7	93.9	1.4013
10	2006.2	49.7	0.8119
10	2007.1	48.2	0.7128
10	2005.1	91.1	1.3256
24	2004.5	76.5833	1.1638
10	2005.3	45	0.7048
20	2004	35.8	0.5658
14	2005	132.6429	1.8016
15	2005.8	59.8	0.8665
10	2006.4	16.8	0.2721
10	2003.8	24.5	0.4124
143	2006.1678	98.958	1.4759
10	2005.9	31.6	0.4974
19	2006.1053	38.8421	0.5687
11	2005.2727	185.5455	2.6013
10	2007.2	39.3	0.6043
14	2007	26	0.425
12	2005.75	26.75	0.4062

21	2004.8095	37.381	0.5296
19	2006.1053	73.4737	1.0577
15	2005.6	45.4667	0.708
10	2003.8	22.2	0.3477
10	2004.1	92.1	1.1766
27	2005.963	54.1481	0.8135
14	2006.7857	73.2143	1.1573
110	2005.4364	76.4727	1.1098
15	2006.6	99.4	1.4897
31	2006.2903	55.1935	0.81
10	2004.8	99.6	1.3438
425	2005.9176	65.3035	0.9703
10	2007.6	67.2	1.0495
27	2006.037	84.963	1.291
54	2005.1296	52.7593	0.8269
12	2006.25	63.5	1.0347
10	2003.2	58.9	0.8003
12	2005.25	41.5	0.6066
60	2006.4333	56.6167	0.8369
23	2006.1304	34.2174	0.4988
36	2005	108.5556	1.5214
12	2004.8333	46.25	0.6699
21	2005.7143	31.8571	0.4279
10	2008	146.2	2.2354
17	2005.0588	54.7059	0.7448
10	2005.2	80	1.2137
10	2005.3	111.7	1.6928
13	2005.4615	72.7692	1.1569
39	2005.0513	63.641	0.9847
12	2005.4167	90.6667	1.2513
12	2003.8333	56.5	0.825
13	2004.9231	120.2308	1.6024
13	2006.9231	82.3846	1.3151
10	2004.9	49.5	0.7775
16	2007.625	74.8125	1.0407
16	2007.625	23.5	0.372
12	2006.3333	40.1667	0.5717
39	2005.3333	37	0.5515
12	2005.3333	23.9167	0.3611
17	2005.3529	25.5294	0.3469
10	2005.3	138.8	1.8853
19	2004.8421	70.5789	0.9615
23	2005.8696	35.5652	0.5035
14	2003.2143	68.1429	0.9259
29	2005.6897	30.7931	0.4659
16	2006.375	33.125	0.5043
846	2006.3251	75.9326	1.1333

203	2006.1429	101.2709	1.4578
13	2007	75.6923	1.0893
13	2005.6154	44	0.6427
24	2005.5	89.2917	1.2109
13	2005.5385	36.6154	0.5564
11	2003.3636	38.1818	0.4783
10	2005.4	59.3	0.8255
12	2003.75	37	0.5243
77	2006.5584	85.9221	1.2879
348	2005.9109	87.5747	1.2806
73	2006.3014	55.5479	0.8486
11	2005.1818	35.5455	0.4752
19	2005.8421	50.5263	0.6813
16	2006.1875	35.3125	0.5098
16	2005.25	59.9375	0.9245
60	2005.5	92	1.3853
133	2005.8496	59.0526	0.8931
57	2006.3158	63.5439	0.9713
19	2006.5263	72.4737	1.0733
12	2005.75	71.5	0.9121
21	2006.5238	36	0.532
12	2005.6667	184.75	2.8173
25	2005.04	115.08	1.5334
14	2004.1429	79.6429	1.144
12	2006.3333	59.5	0.9091
34	2005.0294	49.1176	0.7206
14	2006.0714	37.8571	0.5597
13	2005.3846	38.8462	0.5568
10	2005.1	66.9	0.8255
36	2005.7778	113	1.6251
28	2005.3929	101.7143	1.3615
13	2007.3846	77.6154	1.189
44	2006.2273	59.1818	0.9099
10	2006.1	140.3	2.1865
34	2004.2941	46.4118	0.6323
27	2005.1481	112.5185	1.5286
206	2005.4175	49.5243	0.7521
28	2005.8571	74.0357	1.1245
10	2006.5	45.7	0.6975
10	2003.7	40.5	0.6094
14	2006.2857	73.2857	1.0161
12	2007.5	34.3333	0.5375
21	2006.0952	67	1.0438
40	2006.35	73.925	1.0829
322	2005.6832	45.705	0.669
13	2006.6154	23.8462	0.3696
52	2005.5769	40.2885	0.5734

16	2002.6875	57.375	0.9018
23	2006.4348	83.3478	1.3141
18	2005	89.7222	1.33
57	2005.4737	57.6491	0.8345
28	2005.3571	55.8929	0.8378
10	2004.8	84.3	1.2612
15	2005.4667	52.9333	0.8229
15	2005	76.4	0.9569
16	2007.5	64	1.0188
20	2004.65	43.35	0.6467
63	2004.6667	67.5873	1.0031
10	2004.7	79.9	1.2998
54	2006.0926	85.2778	1.289
12	2005.1667	47.5	0.7146
37	2005.2703	68.1351	1.0585
114	2006.4737	89.3596	1.3514
25	2006.28	36.08	0.5331
29	2005.2759	50.1724	0.7367
27	2004.7407	33.5556	0.4432
17	2006.3529	106.7647	1.6493
11	2006.2727	77	1.1518
23	2003.3043	43.4348	0.6459
19	2006.3684	46.7895	0.7216
13	2005.5385	63.2308	1
24	2005.5417	128.6667	1.8773
36	2005.6944	37.7222	0.5786
28	2006.5714	55.1429	0.7998
12	2006.4167	33.6667	0.5005
20	2003.75	36.85	0.5196
44	2005.5455	61.7955	0.968
12	2007.0833	76.6667	1.175
11	2005.1818	22.6364	0.3341
14	2006.7143	170	2.6413
11	2007.3636	22.6364	0.333
16	2007.1875	94.125	1.3113
19	2004.7895	69.9474	0.9968
33	2006.1515	54.3333	0.8794
21	2006.5714	91.8095	1.303
13	2005.3846	73.1538	1.0629
156	2004.8654	37.1538	0.5477
22	2003.9545	59.3182	0.8297
11	2006.2727	43.4545	0.5897
26	2003.6923	34	0.4885
24	2004.4167	36.5417	0.5579
36	2004.7778	39.9444	0.5682
39	2004.7692	71.5385	1.026
35	2005.3714	87.4286	1.3556

49	2005.4898	50.8776	0.7449
12	2005.9167	69.5833	1.029
118	2005.8305	70.6271	1.0637
41	2006.0976	50.2195	0.7547
57	2006.2632	68.3509	0.9577
99	2005.8586	56.5051	0.8485
11	2005	39.0909	0.5693
40	2006.9	71.9	1.1102
11	2007.9091	209.6364	3.3359
13	2005.1538	31.6923	0.5166
21	2004.9524	24.6667	0.3569
13	2005.7692	330.5385	4.6036
61	2006.9836	34.5082	0.5225
10	2006.1	44	0.6305
13	2005	133.8462	1.6584
57	2005.0351	44.1053	0.6539
12	2005.0833	40.6667	0.6382
185	2005.5027	51.6432	0.7669
32	2004.9062	116.625	1.704
19	2006.2105	26.9474	0.435
31	2005.8065	43.9355	0.6414
13	2005.5385	78.7692	1.2022
79	2005.7089	51.3038	0.7979
16	2005.75	110.75	1.7108
11	2005.9091	37.6364	0.5605
11	2005.5455	133.9091	1.736
11	2005.8182	45	0.6574
169	2005.3373	60.9527	0.872
48	2006.8542	74.625	1.1326
46	2006.4348	45.3261	0.668
16	2007.6875	63.6875	1.0106
13	2004.2308	99.8462	1.4565
36	2006.5833	84.6389	1.2108
24	2005	39.375	0.5444
11	2005.7273	73.4545	0.9522
10	2007.4	112.4	1.7894
15	2005.2	50.3333	0.6961
13	2005.5385	51.8462	0.6997
72	2005.3472	43.5556	0.6538
11	2005.0909	298.5455	4.2459
12	2007.6667	110	1.672
11	2004.0909	68.1818	0.937
98	2006.4286	109.5918	1.6654
59	2005.339	73.4576	1.1017
17	2004.2353	82.8235	1.072
13	2006.3846	36.5385	0.5871
26	2006	81.5385	1.1496

25	2005.76	101.56	1.4374
70	2005.8286	95.0143	1.3455
11	2003.4545	62.3636	0.9037
18	2005.1667	45.0556	0.6494
35	2005.4286	52.9143	0.79
32	2005.5	67.2188	0.9198
60	2005.5833	75.2167	1.1016
14	2004.8571	33.0714	0.4898
11	2004.2727	129.0909	1.7894
433	2006.127	65.9908	0.9914
22	2005.7727	98.7727	1.5093
10	2007.4	43.2	0.6477
11	2005.2727	158.8182	2.4218
62	2005.0323	87.9032	1.3352
11	2007	209.2727	2.9463
42	2006.7857	117.4524	1.7634
16	2003.5625	77.75	1.0863
16	2005.0625	50.3125	0.7305
11	2007.7273	69.8182	1.1386
10	2004.3	116.8	1.4948
20	2006.2	43.85	0.6328
17	2005.5294	27.6471	0.3874
10	2005.6	43.7	0.6418
21	2005.8095	54.5714	0.858
14	2006.0714	48.6429	0.7054
10	2004.9	484.6	6.29
21	2006.7143	110.3333	1.6641
35	2005.0571	89.3143	1.2882
54	2004.963	45.463	0.6441
85	2005.1529	59.9529	0.8951
23	2002.8696	41	0.6622
45	2006.2889	60.3111	0.8584
10	2006.9	66.3	1.0194
11	2004.2727	21	0.3077
220	2006.1182	83.2273	1.2242
14	2004	86.8571	1.2722
22	2006.3182	40.2727	0.644
79	2006.038	56.6329	0.8565
18	2006.3333	103.8333	1.497
10	2006.6	24.2	0.3893
11	2006.6364	59.4545	0.9419
10	2006.6	34.7	0.5016
12	2003.9167	59.9167	0.8641
15	2006.4	35.8667	0.5288
10	2006.5	35.8	0.5436
68	2005.2206	51.5735	0.7836
28	2005.6071	53.8571	0.7475

15	2006.4	65	0.9175
126	2005.8889	56.3254	0.8246
59	2006.1864	81.5593	1.1805
23	2006.6087	86.8261	1.2984
25	2004.76	65.24	1.0032
17	2007.2353	212	3.0264
19	2005.3684	60.1579	0.8693
10	2005.1	45.1	0.7117
11	2004.8182	25.5455	0.3709
10	2006.2	41.4	0.6397
10	2004.1	178.8	2.275
12	2005.8333	97.3333	1.4137
37	2005.8919	54.8919	0.7658
14	2005	30	0.4342
15	2006.2	60.9333	0.9645
13	2005.1538	48.0769	0.7001
17	2005.6471	92.0588	1.3327
120	2005.0167	134.8583	1.9012
16	2003.5	125	1.5947
12	2005.4167	158	2.2124
13	2006.6154	78.6923	1.0695
10	2004	125	1.8372
116	2005.1638	75.0259	1.1191
11	2004.7273	60.5455	0.9206
19	2006.4211	39.9474	0.6048
46	2006.6304	44.5	0.6893
18	2005.8333	86.8889	1.3233
12	2006.8333	56.8333	0.8159
21	2006.619	100.2381	1.5341
23	2005.3043	104.0435	1.4215
14	2004	160.5	2.1981
12	2006.75	64.6667	0.9682
15	2006.2667	58.5333	0.9039
17	2005	21.0588	0.3416
11	2005.0909	55.1818	0.8145
29	2004.6897	37.4483	0.5607
13	2005.3846	50.7692	0.7398
27	2005.963	62.5556	0.9268
11	2007.6364	36.8182	0.5583
10	2004.5	72.5	1.119
11	2006.0909	42.7273	0.6667
222	2005.7748	74.6036	1.089
170	2005.6882	104.3824	1.5367
12	2006.9167	63.25	0.8795
120	2006.05	74.0917	1.0898
10	2006.1	48.1	0.7363
21	2005.9048	30.5238	0.4609

15	2007	54.5333	0.8357
118	2005.8051	52.3559	0.8079
66	2005.1364	35.5606	0.5313
12	2005.6667	32.1667	0.4584
10	2006.4	49	0.7904
850	2006.1541	76.8588	1.146
15	2005.2667	46.7333	0.7188
10	2006.3	47.1	0.6662
32	2005.9688	62.4375	0.982
99	2005.5556	57.3636	0.8621
150	2005.5733	67.1733	1.0038
47	2005.4043	44.6383	0.6545
30	2005.8667	37.8333	0.5741
11	2006.9091	98.2727	1.4604
20	2006.9	109.55	1.6728
11	2004.5455	110.6364	1.6069
19	2005.6316	44.2632	0.725
29	2004.6897	48.4483	0.6658
14	2005.1429	83	1.2831
40	2004.725	109.725	1.6448
52	2005.9615	92.5385	1.3626
16	2006.0625	47.6875	0.7074
35	2006.4571	40.2286	0.5851
36	2006.9444	46.8056	0.7405
54	2005.4074	63.6852	0.8926
26	2005.7308	55.6538	0.8048
10	2007.4	60.2	0.8799
20	2006.35	60.1	0.8647
17	2004.2941	40.4118	0.5628
13	2006.6923	56.6923	0.8599
12	2006	71.6667	0.9853
13	2005.4615	115.2308	1.6683
12	2006.0833	48.25	0.7251
13	2005.0769	36.5385	0.5936
22	2006.8636	36.2727	0.5574
13	2003.3846	80.0769	1.3301
24	2006.5	109.625	1.6759
14	2006.5	78.2857	1.217
41	2006.4878	70.439	1.0714
16	2003.625	23.6875	0.313
12	2007.1667	64.1667	0.8707
21	2005.5714	33.4762	0.5301
16	2005.9375	108.1875	1.6213
39	2004.8205	35.8462	0.5197
19	2005.2632	63.6842	0.9725
12	2007.1667	22.5	0.3397
26	2006.1923	67.3846	0.9555

34	2005.8529	51.1471	0.7845
71	2005.9437	60.2254	0.9234
26	2006.0769	41.7308	0.6117
12	2003.3333	39.25	0.5866
20	2004.4	50.25	0.7285
14	2004.8571	183.7143	2.633
10	2003.9	37.1	0.5171
48	2005.2708	59.4583	0.8951
24	2005.625	39.7083	0.6418
12	2003.1667	43.5833	0.6214
75	2006.4533	71.6133	1.0459
11	2006.7273	234.8182	3.341
11	2006.8182	34.7273	0.5311
17	2005.1765	31.4706	0.5158
21	2006.5714	31.7143	0.4862
14	2005.9286	43.0714	0.6947
56	2006.0536	72.9286	1.0935
16	2005.75	36.5	0.5301
11	2005.0909	56.8182	0.7924
14	2006.3571	21.4286	0.3073
14	2006.7857	103.1429	1.5469
11	2007	72.8182	1.0874
10	2007.4	130.8	1.9372
59	2005.661	46.7458	0.6956
18	2005.2778	36.0556	0.5873
18	2006.0556	38.1667	0.5315
10	2005.9	33.5	0.469
15	2005.4667	27.6667	0.3763
10	2006	38.5	0.6106
12	2005.4167	131.9167	1.9685
30	2005.4333	97.5	1.4523
27	2006	59.7037	0.9085
136	2006.4118	62.5368	0.9594
22	2005.3636	49.4091	0.7199
35	2005.8286	58.0571	0.887
15	2005.2	41.5333	0.6359
12	2004.8333	72.0833	1.074
49	2005.4082	69.6735	1.0238
49	2005.7551	57.3265	0.8103
13	2005.3846	29.0769	0.4239
115	2006.2087	86.5565	1.2701
15	2006.5333	73.2667	1.1489
11	2007.4545	69.5455	1.0147
370	2005.1919	53.5919	0.7897
14	2005	57.2857	0.9465
16	2005.3125	40.6875	0.5815
100	2005.59	47.62	0.7256

12	2007.1667	27.0833	0.4198
14	2007.2857	182.5	2.6548
11	2007.1818	74.3636	1.1178
10	2005.2	16.7	0.2669
32	2006.7188	41.0312	0.6297
91	2005.967	66.7473	0.9971
12	2006.6667	34.5833	0.5338
16	2005.375	69.9375	0.952
11	2006	56	0.8596
16	2004.625	34.25	0.4816
49	2005.4694	72.9184	1.0317
43	2005.4884	33.5349	0.5178
15	2004.7333	65.6667	0.9964
18	2004.0556	38.4444	0.5693
11	2004.2727	66.0909	0.9273
73	2005.4658	78.8493	1.1757
17	2005.6471	103.5882	1.4919
14	2005.7143	54.6429	0.8891
42	2006.1667	91.9048	1.3551
41	2006.561	79.8049	1.2324
17	2006	32.6471	0.5304
21	2006.0476	36	0.5365
14	2006.2143	60.2857	0.9735
36	2005.8889	59.5278	0.9228
24	2006.125	96.4583	1.4132
10	2007.8	43.3	0.6661
25	2005.96	60.32	0.9006
10	2005.8	31.7	0.4413
24	2005.7917	28.9167	0.4519
15	2005.0667	33.2667	0.4569
16	2005.8125	129.25	1.8515
15	2005.9333	63	0.8788
18	2005.1667	65.2222	0.9363
15	2005.4	96.6	1.3826
23	2005.8696	45.1739	0.669
15	2006.4667	88.3333	1.262
167	2005.1497	108.503	1.5435
11	2005.3636	32.6364	0.447
11	2005.1818	123.5455	1.7964
34	2005.2353	50.8235	0.7284
11	2006.0909	54.8182	0.8125
14	2005.4286	29.7143	0.4402
11	2004.9091	25.8182	0.4038
25	2004.44	42.16	0.6139
259	2006.3745	115.749	1.7112
122	2005.8607	71.377	1.0842
12	2004.5833	87.8333	1.175

93	2005.914	88.8495	1.3206
23	2005.6087	69.6087	1.0655
69	2005.2754	138.5072	1.9269
18	2007.3889	66.3889	1.0742
11	2007.7273	26.5455	0.4159
10	2006.1	70.6	0.942
39	2006.2821	66.6154	1.015
35	2005.7429	49.3143	0.7412
10	2005.4	41.5	0.6374
24	2006.7917	67.5417	1.0242
369	2006.4173	64.3333	0.9622
12	2005.5	95.4167	1.4668
33	2005.9697	62.3333	0.9616
11	2006.8182	45	0.6494
55	2005.4	49.6909	0.7516
15	2005.8667	76.3333	1.1255
29	2005.1724	47.9655	0.7223
35	2006.5429	151.1143	2.1211
52	2006.0769	57.6923	0.8664
54	2006.9074	65.3889	0.9733
13	2005.2308	44.6154	0.6434
10	2005.6	30.5	0.4232
10	2006.6	90.4	1.2889
20	2005.3	119.4	1.8035
18	2005.3333	111.4444	1.7738
17	2004.7059	56.1176	0.9132
12	2006	29.6667	0.4316
21	2006.2381	26.8571	0.4025
21	2005.5238	119	1.8384
13	2006.2308	82.0769	1.2006
37	2006.0811	94.7027	1.4442
11	2006.4545	46	0.6834
11	2006.2727	59.0909	0.9104
36	2006.4444	60.9167	0.9129
25	2005.76	66.4	0.9689
13	2006.9231	235.4615	3.2581
34	2004.3824	36.1176	0.5378
63	2006.0635	52.3016	0.79
37	2005.7297	36.3514	0.5153
14	2005.5	102.6429	1.5158
11	2005.7273	58.9091	0.9221
13	2004.0769	69	0.9742
46	2006.9783	46.2391	0.7035
11	2006.9091	55	0.8328
12	2007.0833	61.75	0.8813
11	2003.8182	51.3636	0.795
34	2005.9118	62.9118	0.933

36	2005.3611	83.4444	1.1088
26	2005.7692	65.2308	0.9256
19	2006.1579	90.5789	1.2729
56	2005.8571	58.2679	0.8391
41	2006.2683	65.2927	0.971
10	2002.5	55.7	0.7753
18	2005.5556	52.3333	0.8124
12	2004.0833	87.3333	1.4064
90	2006.4444	103.2	1.5389
61	2005.918	78.6721	1.1631
11	2005.3636	32.0909	0.4797
12	2005.5833	94.3333	1.3528
13	2004.3077	85.3077	1.1547
15	2005.8667	71.2667	0.9527
109	2006.9725	113.7248	1.6456
19	2006.5789	95.9474	1.5368
14	2007.5	62.2857	0.9038
27	2006.7407	39.6667	0.6122
31	2004.3871	59.4516	0.896
104	2005.7885	66.5673	0.9903
12	2007.1667	32.0833	0.4744
20	2005.25	88.8	1.3758
17	2005.6471	81.8824	1.2638
14	2005.4286	57.7857	0.8437
11	2005.1818	27.0909	0.383
15	2004.8	46.8667	0.5759
11	2007.2727	59.8182	0.9204
35	2006.1143	55.2286	0.8478
10	2006.5	53.8	0.7848
36	2005.9167	39.2778	0.5803
61	2005.6885	47.8033	0.7014
13	2005.3077	57.7692	0.8197
24	2007.2083	64.4583	0.9654
11	2003	137.6364	1.6339
32	2007.1875	54	0.8077
12	2004.9167	55.9167	0.9093
11	2006.5455	72	1.1183
89	2005.2135	48.3933	0.7336
42	2006.1905	95.4762	1.4911
219	2005.6027	62.1872	0.9147
91	2005.8462	52.0989	0.7488
16	2004.625	45.0625	0.6507
11	2005.2727	41.5455	0.6276
22	2007.7273	127.9545	1.9743
25	2004.44	47.2	0.727
11	2006	21.5455	0.3463
15	2006.3333	291.9333	4.04

16	2005.0625	97.875	1.3549
41	2006.3902	48.7317	0.7783
12	2004.4167	70	0.9364
19	2005.3158	46.2105	0.7398
15	2007.5333	91.8667	1.3552
14	2005.5714	38	0.5913
174	2005.4943	46.0747	0.6783
213	2005.2958	55.1972	0.8272
90	2006.1556	56.1556	0.7975
10	2005.2	115.1	1.5957
21	2005.9048	96.381	1.3463
16	2007.625	45.6875	0.7168
74	2006.4595	60.9865	0.908
33	2005.8182	43.1818	0.6468
10	2005.7	44.6	0.6922
18	2005.5556	54.8889	0.8394
14	2005.5	73.6429	1.019
13	2007.1538	47.2308	0.7375
27	2006.1481	37.7037	0.5352
14	2006.7857	46.0714	0.696
12	2006.0833	96	1.4389
37	2005.3514	77.973	1.1202
38	2006.8947	74.1316	1.0916
10	2006.4	82.6	1.2782
17	2005.2941	26.2941	0.3851
13	2007	24.9231	0.3746
10	2002.3	270.3	3.3857
24	2006.3333	100.0417	1.388
15	2007.2667	53.1333	0.8149
21	2006	53.5238	0.7982
43	2006.6279	83	1.2596
20	2005.8	36.1	0.5535
11	2007.4545	69	1.0282
39	2006.4103	108.5897	1.6213
11	2006.8182	111.4545	1.7185
14	2004.2857	77.7143	1.1926
24	2004.625	58.5417	0.8727
28	2005.1786	56.6429	0.9211
61	2005.4426	81.6393	1.2716
22	2005.4545	46.1818	0.6655
35	2005.6857	37.3714	0.5639
93	2006.5054	67.6559	0.9944
34	2006.3824	38.7353	0.5821
10	2004.7	71.6	1.0374
20	2004.7	108.75	1.5171
13	2005.3846	77	1.1867
12	2004.6667	55.4167	0.7509

17	2006.1765	55.4118	0.8068
10	2006.8	66.8	0.9172
14	2006.5	38	0.5236
14	2005.0714	33.7143	0.49
30	2006.5333	77.2	1.141
76	2005.6974	67.1184	0.977
18	2004.6667	48.5556	0.6852
32	2005.4375	49.3438	0.7257
10	2004.8	76	1.0145
18	2007.4444	96.8333	1.4799
246	2005.7967	50.7886	0.7479
13	2005.3846	69.5385	1.0453
10	2005.1	85.5	1.2002
43	2005.3256	42.8372	0.6289
14	2005.3571	67.3571	1.0412
127	2005.2677	68.6142	1.0313
60	2006.05	57.8167	0.9021
29	2006.1379	48.3448	0.7429
56	2005.8929	84.5179	1.1861
10	2005.7	117	1.5194
10	2006.1	232.5	3.2563
13	2005.3077	101	1.4631
31	2005.0323	80.9355	1.0967
21	2005.9048	27	0.3905
43	2005.0233	43.4651	0.661
63	2005.0476	64.2857	0.9929
28	2005.1071	27.75	0.4109
22	2006.5909	74.9545	1.1342
23	2004.6087	140.4348	2.0825
18	2005.1667	55.1667	0.7439
27	2007.2593	72.037	1.1044
27	2007.2963	165.6667	2.438
48	2005.625	86.7083	1.2637
44	2006.9091	118.7955	1.8406
162	2005.037	103.9938	1.53
22	2006.5455	136.9545	2.0533
90	2005.9889	49.0556	0.7196
15	2005	109.8	1.5621
11	2005.7273	53.4545	0.8866
25	2005.48	34.68	0.5033
11	2006.4545	85.0909	1.3011
14	2007	76.5714	1.0636
48	2006.875	52.0417	0.7733
13	2004.8462	34.4615	0.4996
27	2004.8519	40.7407	0.6043
10	2006.3	34.5	0.5206
41	2005.5366	36.4634	0.5629

34	2005.5882	28.6176	0.4441
16	2007.3125	76.5625	1.1434
11	2006.0909	69.5455	0.9836
13	2005.6154	15.4615	0.246
16	2004.625	37.0625	0.5591
10	2005.8	30.5	0.4589
22	2005.2273	97.3182	1.4526
19	2004.7368	21.7895	0.3462
13	2005.1538	30.7692	0.4272
11	2006.8182	92.3636	1.314
37	2006.7838	31.7027	0.473
10	2004.8	46.2	0.6333
